# Supplementary material for: At the frontlines of digitisation: a qualitative study on the challenges and opportunities in maintaining accurate, complete and timely digital health records in India’s government health system
Source: BMJ Open. 2022 Feb 9;12(2):e051193. doi: 10.1136/bmjopen-2021-051193 (PMC8830249; doi:10.1136/bmjopen-2021-051193)
Supplement: Supplementary data [file bmjopen-2021-051193supp002.pdf]

Annex 1. Acronyms and key terms used

ASHA	Accredited Social Health Activist (ASHA Sahyogini in Rajasthan), a female community health worker
AWW or Anganwadi	Anganwadi Worker, a female community nutrition and preschool functionary
ANM	Auxiliary nurse midwife, a female frontline health worker who provides outreach and some clinical maternal and child health care
DEO	Data entry operator
FGD	Focus group discussion
FLHW	Frontline health workers
HIS	Health information systems
ICT	Information communication technologies
IDI	In-depth interview
MCP card	Mother Child Protection card, a health tracking and information card filled out by the frontline worker and kept by the pregnant woman
MCTS	Maternal and Child Health Tracking System / Reproductive and Child Health system. MCTS is an initiative of Ministry of Health & Family Welfare to leverage information technology for ensuring delivery of full spectrum of healthcare and immunization services to pregnant women and children up to 5 years of age. It facilitates and monitors service delivery and also establishes a two way communication between the service providers and beneficiaries (Ref: Digital India, MoHFW, http://nrhm-mcts.nic.in/Home.aspx [accessed 3 Dec 2021])
mHealth	Mobile health, use of mobile and wireless technologies to support the achievement of health objectives
MO	Medical Officer (a doctor)
PCTS	Pregnancy, Child Tracking, and Health Services Management System (Rajasthan's version of MCTS, which also syncs with MCTS)
RCH portal	Reproductive and Child Health portal. RCH (an upgraded version of MCTS) has been designed for early identification and tracking of the individual beneficiary throughout the reproductive lifecycle. While MCTS tracks every pregnancy, RCH portal tracks a woman throughout her reproductive life cycle, thus making available information on obstetric history. (Ref: RCH Portal & Allied Initiatives of MoHFW, 2018, https://icds-wcd.nic.in/nnm/Events/TechThon/ExistingTechnologyPlatform_for_HealthSchemes_MoHFW-28-06-2018.pdf [accessed 3 Dec 2021])
