# Supplementary material for: At the frontlines of digitisation: a qualitative study on the challenges and opportunities in maintaining accurate, complete and timely digital health records in India’s government health system
Source: BMJ Open. 2022 Feb 9;12(2):e051193. doi: 10.1136/bmjopen-2021-051193 (PMC8830249; doi:10.1136/bmjopen-2021-051193)
Supplement: Supplementary data [file bmjopen-2021-051193supp001.pdf]

At the frontlines of digitization: a qualitative study on the challenges and opportunities in maintaining accurate, complete, and timely digital health records in India's government health system

Supplementary file 1: Sample IDI guide and FGD guide

INTERVIEW GUIDE WITH FRONTLINE PROVIDERS
साक्षात्कार निर्देशिका

Respondent criteria:
जिनका साक्षात्कार लेना है, उनके मानदंड:

- Frontline provider (ANM, ASHA, AWW, nurse, medical officer)
  अगुआ प्रदाता (एएनएम, आशा, एडब्लूडब्लू, नर्स, मेडिकल ऑफिसर)
- Collects MCTS/RCH data from pregnant or newly delivered women
  गर्भवती और नयी प्रसूति महिलाओं से एमसीटीएस/आरसीएच डेटा जमा करें
- Has been in the position for over five years
  अगुआ प्रदाता को कम से कम पांच साल का अनुभव हो

Preamble
प्रस्तावना

Thank you for agreeing to this interview. We're interested in understanding more about the maternal child health tracking system / reproductive child health tracking system, particularly the completeness of mobile phone registration. Please feel free to speak openly about how you feel, in terms of both good things and areas for improvement. There are no right or wrong answers.

इस बातचीत के लिए आप तैयार हुए/हुयीं, इसके लिए आपको धन्यवाद. मैटरनल चाइल्ड हेल्थ ट्रैकिंग सिस्टम/ रिप्रोडक्टिव चाइल्ड हेल्थ ट्रैकिंग सिस्टम को हम ज्यादा से ज्यादा समझने के इच्छुक हैं, विशेषकर मोबाइल फ़ोन पंजीकरण की पूर्णता (कम्पलीटनेस) के बारे में. इस संबंध में आपकी राय क्या है, इसे खुले तरीके से रखने के लिए आप स्वतंत्र हैं. हम दोनों तरह की जानकारियों की आपसे उम्मीद रखती/रखते हैं, वे चीजें जो अच्छी हैं और वैसे हिस्से भी जहाँ सुधार की गुंजाइश है. कोई भी उत्तर सही या गलत नहीं होता है.

1. **Introduction**
   परिचय

Before we discuss the main topics, I would like to learn a bit about you.
मुख्य विषय पर आने से पहले, मैं आपके बारे में कुछ जानना चाहती/चाहता हूँ.

- Could you tell me about yourself – where you're from and how you have come to this position?
  क्या आप अपने बारे में मुझे कुछ बता सकते/सकती हैं? जैसे, आप कहाँ से हैं और इस पद तक पहुँचने की यात्रा कैसे संभव हुयी?

2. **Late ANC registration**
   प्रसवपूर्व जांच के लिए देर से पंजीकरण

One issue we’re interested in is late ANC registration.

लेट एएनसी रजिस्ट्रेशन (प्रसवपूर्व जांच के लिए देर से पंजीकरण) एक ऐसा मुद्दा है, जिसके बारे में हम जानने को इच्छुक हैं.

- When do women here typically register their pregnancies?
  यहाँ की महिलाएं अपने गर्भवस्था को आमतौर पर कब पंजीकृत करवाती हैं?
- Could you tell me about women’s ANC options in terms of private or public care?
  क्या आप महिलाओं के एएनसी के विकल्पों, जैसे निजी या सरकारी, के बारे में मुझे बता सकते/सकती हैं?
- Which government health care providers might women interact with for their pregnancy registration?
  वे कौन-सी सरकारी स्वास्थ्य सेवा संस्थाएं हो सकती हैं, जहाँ महिलाएं अपने गर्भधारण के पंजीकरण के लिए संपर्क कर सकती हैं.
- What are some reasons for late ANC presentation?
  लेट एएनसी प्रेजेंटेशन के लिए कौन-से कारण जिम्मेवार हैं?

3. **Asking women from their mobile phone numbers**
   महिलाओं से उनके मोबाइल फोन नंबर पूछना

During ANC or the registration of a newborn, you often collect the woman’s mobile phone number.
एएनसी या नवजात शिशु के पंजीकरण के दौरान, कर्मचारी महिला का मोबाइल फोन नंबर लेते हैं.

- Could you tell me about how this data is collected?
  ये आंकड़े कैसे जमा किये जाते हैं, क्या इसके बारे में आप मुझे बता सकते हैं?
- What questions do you ask women? How many times do you ask them?
  आप किस तरह के सवाल महिलायों से पूछते हैं? कितनी बार पूछती हैं?
- What are some challenges you have faced in registering a mobile number for each pregnant woman?
  हर गर्भवती महिला का मोबाइल फोन नंबर पंजीकृत करने में आपको कौन-सी चुनौतियों का सामना करना पड़ता है.

[If the respondent says that many women do not have mobiles, probe for other reasons]
जिनसे आप साक्षात्कार कर रहे हैं और वे कहते/कहती हैं कि बहुत सारी महिलायों के पास मोबाइल फ़ोन नहीं है, तब अन्य कारणों की जांच करें

- Among women who do have access to a mobile phone, what are some reasons they may not register it?

वैसी महिलाएं जिनके पास मोबाइल फ़ोन है, फिर भी शायद वे अपने मोबाइल फोन नंबर को पंजीकृत नहीं करवाती हैं, इसके क्या कारण हो सकते हैं?

- What about women not knowing their mobile number? How do health workers manage this?
  कुछ महिलायें ऐसी भी हैं जिनके पास मोबाइल फ़ोन नंबर तो है लेकिन उन्हें नंबर याद नहीं रहता, ऐसी स्थिति में स्वास्थ्यकर्मी इसे कैसे मैनेज करते/करती हैं?
- What about women not wanting to give their mobile numbers? What could be some reasons for this? How do health workers manage it?
  वैसी महिलाएं जो अपना मोबाइल फ़ोन नंबर देना नहीं चाहती हैं, इसके क्या कारण हो सकते हैं? ऐसी स्थिति में स्वास्थ्यकर्मी इसे कैसे मैनेज करते/करती हैं?

- Which women might be resistant or unable to register their mobile number?

  वे कौन-सी महिलाएं हैं जिन्हें अपने मोबाइल नंबर को पंजीकृत करने बाधाएं आती हैं या पंजीकृत करवा पाने में अक्षम हैं?

4. **Gender considerations**
   लिंग आधारित सोच

In a family, men and women often have different access to and use of mobile phones.
एक परिवार में स्त्री और पुरुष के मोबाइल रखने और उसे इस्तेमाल करने के मामले में भेद-भाव अक्सर ही दीखता है.

- Could you tell me about any differences you have noticed between pregnant women versus the men in their families in terms of access to mobile phones?
  मोबाइल फ़ोन इस्तेमाल करने के मामले में परिवार के भीतर गर्भवती महिला और पुरुष के बीच किसी तरह के अंतर को आपने किया है?
- How about in terms of knowledge of and comfort with using mobile phones – how is this different between pregnant women versus men in their families?
  परिवार के भीतर गर्भवती महिला और पुरुष के बीच मोबाइल फोन इस्तेमाल करने की सहजता और जानकारी अलग-अलग कैसे है?
- Which families or communities might have more differences between male and female access to phones? Which families might have less of a difference? Why?

मोबाइल फ़ोन इस्तेमाल करने के मामले में स्त्री-पुरुष के बीच सबसे ज्यादा अंतर किन परिवारों और समुदायों में है? किन परिवारों/समुदायों में यह अंतर कम है? ऐसा क्यों?

- When seeking mobile phone numbers in order to register women in MCTS/RCH, in what ways do you involve male family members? [E.g. the ANM might send the woman to ask her husband for the mobile number in order to use it]
  एमसीटीएस/आरसीएच में महिलायों के पंजीकरण के दौरान मोबाइल फ़ोन नंबर जानने के लिए स्वास्थ्य कर्मचारी परिवार के पुरुष सदस्यों को किस तरह शामिल (इन्वॉल्व) करते हैं? [जैसे, एएनएम किसी महिला को अपने पति के पास भेज यह पूछने के लिए भेज सकती है कि मोबाइल फ़ोन नंबर क्या है, जिसका इस्तेमाल किया जा सके]

5. **Data flow**
   आंकड़ों की यात्रा

Could you walk me through the process from when a woman gives her mobile number until it is registered in the electronic record system?
जब कोई महिला अपना मोबाइल नंबर देती है, और वह नंबर इलेक्ट्रॉनिक रिकॉर्ड सिस्टम में पंजीकृत होता है, तब तक की पूरी प्रक्रिया पर क्या आप कुछ रौशनी डाल सकते/सकती हैं?
Probe on each step:
प्रत्येक चरण की जांच करें:

- E.g. So first you write the number down.
  जैसे, सबसे पहले जब एएनएम लिखा जाता है.
  - Where is it written? Is it written in more than one place?
    यह कहाँ लिखा जाता है?
  - How do you feel about this process?
    इस प्रक्रिया के बारे में आप क्या सोचते/सोचती हैं?
  - What are some things that may go wrong at this stage?
    वे कौन-सी चीजें हैं जो शायद इस चरण में गलत हो सकती हैं?
  - How could this process be improved?
    इस प्रक्रिया में सुधार कैसे लाया जा सकता है?

- Then what do you do with the record?
  तब एएनएम रिकॉर्ड के साथ क्या करता है?
  - How do you feel about this process?
    इस प्रक्रिया के बारे में आप क्या सोचते/सोचती हैं?
  - What are some things that may go wrong at this stage?
    वैसी कौन-सी चीजें हैं जो इस चरण में गलत हो सकती हैं?
  - How could this process be improved?

इस प्रक्रिया में सुधार कैसे लाया जा सकता है?

- Then how is the record is entered into the electronic system?
  रिकॉर्ड को इलेक्ट्रॉनिक सिस्टम में फीड कैसे किया जाता है?
  - What is the procedure?
    इसकी प्रक्रिया क्या है?
  - Who is involved?
    इस प्रक्रिया में कौन लोग शामिल होते/होती हैं?
  - How do you feel about this process?
    इस प्रक्रिया के बारे में आप क्या सोचते/सोचती हैं?
  - What are some things that may go wrong at this stage?
    वे कौन-सी चीजें हैं जो इस चरण में गलत हो सकती हैं?
  - How could this process be improved?
    इस प्रक्रिया में सुधार कैसे लाया जा सकता है?

6. **How MCTS/RCH has changed the work environment**
   एमसीटीएस/आरसीएच ने काम के माहौल को किस तरह/कैसे परिवर्तित किया है

MCTS/RCH are new systems and there are often challenges associated with introducing new systems.
एमसीटीएस/आरसीएच नयी प्रणाली है और नयी प्रणालियों के सामने आने से जुडी चुनौतियां भी वहां होती हैं.

- Can you tell me about how MCTS/RCH has changed pregnancy and birth documentation and registration?
  क्या आप मुझे बता सकते हैं कि एमसीटीएस/आरसीएच ने गर्भधारण और जन्म से सम्बंधित दस्तावेजीकरण और पंजीकरण को कैसे परिवर्तित कर दिया है?
- How has MCTS/RCH changed your work?
  मसीटीएस/आरसीएच ने आप के कामकाज को कैसे परिवर्तित किया है?
- How has MCTS/RCH changed the work done my others in the health system?
  स्वास्थ्य विभाग के अन्दर कार्यरत अन्य बहुत सारे लोगों के कामकाज को एमसीटीएस/आरसीएच को कैसे परिवर्तित किया है?
- How were things before?
  इसके पहले चीजें कैसी थीं?
- How do you feel about these new systems?
  इस नयी व्यवस्था-प्रणाली के बारे में आप क्या सोचते/सोचती हैं?

7. **Training and supervision for data quality**
   प्रशिक्षण और आंकड़े की गुणवत्ता की देख-रेख

- What kind of training or information have you received about MCTS/RCH?

एमसीटीएस/आरसीएच संबंधी किस तरह की सूचना और प्रशिक्षण आपने पायी?

- How do you feel about the training and information given to you and others? What could make it better?
  एमसीटीएस/आरसीएच संबंधी किस तरह की सूचना और प्रशिक्षण अन्य लोगों ने पायी? इसे और बेहतर कैसे बनाया जा सकता है?
- What are some ways that the accuracy and completeness of data in MCTS/RCH, including mobile phone numbers, can be assured?
  वे कौन से तरीके हैं जिससे मोबाइल नंबर सहित एमसीटीएस /आरसीएच के डेटा को सटीक और पूर्ण रूप से बताया जा सकता है.
  - How do you feel about the quality assurance processes?
    गुणवत्ता जांचने वाली प्रक्रियाओं के बारे में आप की क्या राय है?
  - In an ideal world, what would happen to ensure accurate and complete data is registered in MCTS/RCH?
    एक आदर्श वातावरण में, एमसीटीएस/आरसीएच में सटीक और पूर्ण डेटा पंजीकृत करने के लिए क्या-क्या करना होगा?

8. **Use of mobile phone number**
   मोबाइल फ़ोन नंबर का इस्तेमाल

So now I'd like to ask you about the benefit of MCTS/RCH.
अब मैं आपसे एमसीटीएस/आरसीएच के फायदे के बारे में पूछना चाहती/चाहता हूँ.

- What are some reasons why the government wants to register women's phone numbers?
  वे कौन-से कारण हैं कि सरकार महिलायों के फ़ोन नंबर को पंजीकृत करना चाहती है?
- What are some benefits for you if the woman's phone number is registered?
  यदि महिलायों के फ़ोन नंबर पंजीकृत होते हैं तो इसमें आपके लिए फायदेमंद क्या है?
- What are some benefits for the woman?
  पंजीकृत होने वाली महिलायों के लिए कौन-से फायदे हैं?

9. **Corrections and updates to registered mobile numbers**
   पंजीकृत मोबाइल नंबरों को संशोधित कर उसे अपडेट करना/करवाना

Women may change their numbers, may acquire a phone, or may find out that their mobile number has been entered incorrectly in the system.
महिलाएं शायद अपना नंबर बदल ली हों, शायद एक मोबाइल ली हो या उसे पता चला हो कि सिस्टम में जो नंबर उसके नाम के साथ दर्ज है, वह सही नहीं है.

- What is the procedure for updating or correcting records? Probe on each step and what the challenges might be.
  रिकॉर्ड को अपडेट करने या सही करने की प्रक्रिया क्या है? हर चरण की जांच करें कि और क्या परेशानियां हो सकती हैं.
- How do you feel about this process?
  आप इस प्रक्रिया के बारे में क्या सोचते हैं?
- What are some reasons women may not correct or update the record?
  वे कौन-से कारण हैं शायद जिसके चलते महिलाएं रिकॉर्ड को सही या अपडेट नहीं करती हैं?
- What could make the process easier for women?
  वे कौन-से उपाय हैं जो महिलायों के लिए इस प्रक्रिया को आसान बना सकते हैं?
- Why do you think the process isn’t like this now?
  आप ऐसा क्यों सोचते हैं कि पुराना वाला तरीका अब बदल गया है?

10. Closing
समाप्ति

Thank you for your time and for telling me so much about MCTS/RCH.
आपने मुझे अपना बहुमूल्य समय दिया और एमसीटीएस/आरसीएच के बारे में इतनी बातें बताई, इसके लिए आपके आभारी हैं.

- Would you like to add anything else about the accuracy and completeness of mobile phone registration in MCTS/RCH?
  एमसीटीएस / आरसीएच में मोबाइल फोन के पंजीकरण की सटीकता और पूर्णता के सन्दर्भ में कुछ और जोड़ना चाहेंगे/चाहेंगी?

FOCUS GROUP DISCUSSION (BENEFICIARIES) GUIDE

समूह केन्द्रित बातचीत - निर्देशिका

Participants:
भागीदार:

- Have a mobile phone number

जिनके पास मोबाइल फ़ोन नंबर हो

- Are pregnant or recently delivered

जो गर्भवती हों या जिन्हें अभी-अभी बच्चा हुआ है

- Recently interacted with an ANM, government nurse or doctor

जिन्होंने अभी-अभी एएनएम, सरकारी नर्स या डॉक्टर से संपर्क किया हो

Thank you for coming to this discussion. We're interested in understanding more about how pregnant women interact with government health care workers. This means mostly ANM and ASHA but also maybe the doctor, nurse and anganwadi worker. Please feel free to speak openly about how you feel
आप इस बातचीत के लिए आयीं, इसके लिए आपको बहुत-बहुत धन्यवाद. हम इस बात को अच्छे तरीके से समझना चाहते हैं कि गर्भवती महिलाएं सरकारी स्वास्थ्य-सेवा कर्मियों से किस तरह संपर्क करती हैं. स्वास्थ्य-सेवा कर्मियों से यहाँ मतलब, ज्यादातर, एएनएम और आशा कर्मियों से है, लेकिन शायद वे डॉक्टर, नर्स और आंगनवाड़ी कर्मी भी हो सकती हैं. आप अपनी बात खुल कर कहने के लिए आज़ाद हैं कि आपका अनुभव कैसा रहा.

11. Introduction
परिचय

क्या सभी लोग अपनी उम्र बता सकती हैं और यह भी कि आपको खाने में क्या पसंद है!

12. Late ANC presentation
लेट एएनसी प्रेजेंटेशन

- Around here, when do women generally first meet with ANM didi or ASHA didi about their pregnancy?

अपने गर्भवती होने के सिलसिले में यहाँ आस-पास की महिलाएं पहली बार साधारणतया एएनएम दीदी और आशा दीदी से कब मिलती हैं?

- How do they decide when to meet with ASHA didi? With ANM didi?

एएनएम दीदी और आशा दीदी से मिलना है, इस निश्चय का फैसला वे कैसे करती हैं?

Some women may come at the very beginning of their pregnancy, right when they think they are pregnant. Maybe you did this or someone you know.

कुछ औरतें शायद अपने गर्भधारण के शुरुआत में ही, जैसे ही वे सोचती हैं कि वे गर्भवती हैं, एएनएम दीदी और आशा दीदी से मिलने चली आती हैं. शायद आपने ऐसा किया है या किसी को जानती हैं, जिसने ऐसा किया है.

- What are some reasons that women might come early? Which women might meet ANM didi early?

वैसे कौन-से कारण हैं जिनके चलते महिलाएं जल्द ही एएनएम दीदी से मिलने आ सकती हैं? वे कौन-सी महिलाएं हैं जो एएनएम दीदी से जल्द ही मिल सकती हैं?

Some women may come later, after they have been pregnant for a while.
कुछ महिलाएं शायद बाद में एएनएम दीदी से मिलने आती हैं, जब गर्भधारण किये उन्हें कुछ समय बीत जाता है.

- What are some reasons that women might come late? Which women might meet ANM didi late?

वैसे कौन-से कारण हैं जिनके चलते महिलाएं बाद में एएनएम दीदी से मिलने आ सकती हैं? वे कौन-सी महिलाएं हैं जो एएनएम दीदी से बाद में मिल सकती हैं?

Some women might not meet ANM didi at all.
कुछ महिलाएं एएनएम दीदी से एकदम ही नहीं मिलती हैं.

- What are some reasons that women might not ever meet ANM did? Which women might not meet ANM didi at all?

वैसे कौन-से कारण हैं जिनके चलते महिलाएं एएनएम दीदी से मिलने एकदम ही नहीं आ सकती हैं? वे कौन-सी महिलाएं हैं जो एएनएम दीदी से मिलने एकदम ही नहीं आ सकती हैं?

In some places they say it's better not to tell people about a pregnancy, to avoid bad influences coming.
कुछ जगहों पर लोग कहते हैं कि गर्भवती होने की सूचना लोगों को नहीं देना अच्छा है, ऐसा करके बुरे प्रभावों से बचा जा सकता है.

- Can you tell me about what people say around here? How do people keep safe during pregnancy?

क्या आप मुझे बता सकती हैं कि यहाँ आस-पास के लोग क्या कहते हैं? लोग गर्भावस्था दौरान खुद को कैसे सुरक्षित रखती हैं?

13. Written records

लिखित दस्तावेज

When you visit with ASHA or ANM didi, or even when you visit with the nurse or doctor at the health centre, they ask some questions and write down some things. जब आप आशा दीदी या एएनएम दीदी से मिलने जाती हैं, यहाँ तक कि जब स्वास्थ्य-केंद्र के नर्स या डॉक्टर से मिलने जाती हैं, तब वे कुछ सवाल पूछते हैं और उत्तर सुनते हुए कुछ लिखते रहते हैं.

- Can you recall about what things they ask you?

क्या आप याद करके पता सकती हैं कि उन्होंने ने क्या-क्या पूछा?

If they can't recall, probe or jog memory: Sometimes women might be asked about their last menstrual period or their age. [Show the state's ANC registration card, e.g. the Rajasthan Mamta Card]
यदि वे याद नहीं कर सकती हैं, तब उनके दिमाग की थाह लेने या दिमाग पर जोर डालने की कोशिश करें: कभी-कभी महिलाओं से उनकी उम्र या अंतिम माहवारी कब आई जैसे सवाल पूछे जाते हैं. [राज्य का एएनसी पंजीकरण कार्ड दिखायें. जैसे राजस्थान ममता कार्ड]

- How do women feel when ANM didi/doctor sir asks questions?

एएनएम दीदी/डॉक्टर जब पूछते हैं, तब महिलाएं कैसा महसूस करती हैं?

Sometimes questions can be asked in a nice way and other times they are asked in a way that makes women feel bad, rushed, or unsure.
कभी-कभी सवाल पूछने का उनका तरिका बहुत अच्छा होता लेकिन कभी ऐसा भी होता है कि सवाल पूछने का उनका तरीका महिलाओं पसंद नहीं आता, या ऐसा लगता है कि सवाल पूछने में जल्दबाजी की जा रही, या सवाल स्पष्ट नहीं हो पाता है.

- Can you pretend to be a nurse, ANM or doctor and show me how they ask questions nicely?

क्या आप थोड़े समय के लिए एएनएम दीदी या डॉक्टर बनकर यह बता सकती हैं कि सवाल पूछने का अच्छा तरीका क्या होता है?

- Can you pretend to be a nurse, ANM or doctor and show me how they can ask questions in a bad or not nice way?

क्या आप थोड़े समय के लिए एएनएम दीदी या डॉक्टर बनकर यह बता सकती हैं कि सवाल पूछने का तरीका बुरा है या यह ढंग अच्छा नहीं है.

- Which questions are nice and easy to answer? What are some reasons for this?

वे कौन-से सवाल हैं जो अच्छे हैं और जिसका उत्तर देना आसान है? इसके कारण क्या हैं?

- Which questions might some women find difficult to answer or not nice?

वैसे कौन-से सवाल हैं जिसके उत्तर देने में कुछ महिलाओं को दिक्कत हो सकती है या उत्तर देना अच्छा नहीं है?

- What are some reasons for this?

इसके क्या कारण हैं?

- Which women might struggle a lot to answer questions?

वे कौन-सी महिलाएं जिन्हें सवालों के उत्तर देने में बहुत संघर्ष करना पड़ सकता है?

14. Mobile phone number

मोबाइल फ़ोन नंबर

One thing that women might be asked is for their mobile phone number.
एक बात यह भी है कि महिलाओं से उनके मोबाइल फ़ोन नंबर के बारे पूछा जा सकता है.

- How might women feel when they are asked by ANM didi for their mobile numbers?

एएनएम दीदी द्वारा जब उनके मोबाइल फ़ोन नंबर पूछे जाते हैं तब महिलायों को कैसा लगता है?

- What are some reasons why ANM didi might ask for a mobile number?

वे कौन-से कारण हैं, जिसके चलते एएनएम दीदी मोबाइल फ़ोन नंबर पूछ सकती हैं?

- If she did not have a mobile number, how might she feel? What might she do?

यदि उनके पास मोबाइल फ़ोन नंबर नहीं है तब उन्हें कैसा महसूस होता है? ऐसी स्थिति में वे क्या कर सकती हैं?

Some women might feel happy to give their mobile number, but others might not want to give it.
कुछ महिलाएं एएनएम दीदी को मोबाइल फ़ोन नंबर देने में ख़ुशी महसूस कर सकती हैं, लेकिन कुछ ऐसी भी हो सकती हैं जो मोबाइल फ़ोन नंबर देना नहीं चाहती हों.

- What might be some reasons why a woman would not want to give her mobile number?

वैसे कौन-से कारण हो सकते हैं कि एक महिला मोबाइल फ़ोन नंबर देना नहीं चाहती.

Sometimes a woman wants to give her mobile number but she does not recall what it is.
कभी-कभी कोई महिला अपना मोबाइल फ़ोन नंबर एएनएम दीदी को देना चाहती है लेकिन वह इस बारे में स्पष्ट नहीं होती है कि इससे क्या होगा.

- Could this happen? Tell me about how she would feel. Tell me about what might happen next.

क्या ऐसा हुआ है? यदि हाँ, तो वह यह जानकार कैसा महसूस करेगी, इसके बारे में बताएं. इसके बारे में भी बताएं कि इसके बाद क्या हो सकता है.

15. Corrections and updates to registered mobile numbers

पंजीकृत मोबाइल नंबरों को संशोधित कर उसे अपडेट करना/करवाना

Sometimes a person will get a new SIM so the mobile number will change. Sometimes they realize the mobile number recorded with the government is not correct. Either way, this means that the government has got the wrong number.

कभी-कभी कोई नया सिम लेगा, ऐसी स्थिति में मोबाइल नंबर चेंज हो जाएगा. कभी-कभी उन्हें लगता है कि सरकारी दस्तावेजों में दर्ज उनका मोबाइल नंबर सही नहीं है. अन्य तरीके से कहें तो इसका मतलब यह है कि सरकार को जो नंबर उपलब्ध है, वह गलत है.

- How might a woman feel if this happened? E.g. if she realized the government recorded a different number - an old number or a wrong number?

यदि ऐसा हुआ है, तब एक महिला के लिया यह अनुभव कैसा होता है? जैसे, यदि उसे इसका भान हो गया कि सरकार के यहाँ दर्ज उसका मोबाइल नंबर पुराना या गलत है?

- What could she do? Who might she speak to? What might happen if she tried to change it? Where would she go?

ऐसी स्थिति में वह क्या कर सकती है? वह कौन हो सकता है जिससे वह इसके सम्बन्ध में बात कर सकती है? क्या हो सकता है, यदि उसने मोबाइल नंबर चेंज करवाने की कोशिश की? वह कहाँ जायेगी?

- Some women might not try to change or add their number, if they realized it was wrong. Why might this be?

कुछ औरतें अपने मोबाइल नंबर को बदलवाने या जोड़वाने की कोशिश नहीं कर सकती हैं, बाद उन्हें पता लगा कि यह गलत था. ऐसा क्यों हुआ?
