# Supplementary material for: At the frontlines of digitisation: a qualitative study on the challenges and opportunities in maintaining accurate, complete and timely digital health records in India’s government health system
Source: BMJ Open. 2022 Feb 9;12(2):e051193. doi: 10.1136/bmjopen-2021-051193 (PMC8830249; doi:10.1136/bmjopen-2021-051193)
Supplement: Author's manuscript [file bmjopen-2021-051193.draft_revisions.pdf]

BMJ Open is committed to open peer review. As part of this commitment we make the peer review history of every article we publish publicly available.

When an article is published we post the peer reviewers' comments and the authors' responses online. We also post the versions of the paper that were used during peer review. These are the versions that the peer review comments apply to.

The versions of the paper that follow are the versions that were submitted during the peer review process. They are not the versions of record or the final published versions. They should not be cited or distributed as the published version of this manuscript.

BMJ Open is an open access journal and the full, final, typeset and author-corrected version of record of the manuscript is available on our site with no access controls, subscription charges or pay-per-view fees (http://bmjopen.bmj.com).

If you have any questions on BMJ Open's open peer review process please email
info.bmjopen@bmj.com

BMJ Open

At the frontlines of digitization: The challenges and opportunities in maintaining accurate, complete, and timely digital health records in India's government health system

Journal:	*BMJ Open*
Manuscript ID	bmjopen-2021-051193
Article Type:	Original research
Date Submitted by the Author:	16-Mar-2021
Complete List of Authors:	Scott, Kerry; Johns Hopkins University Bloomberg School of Public Health<br>Ummer, Osama; Oxford Policy Management; BBC Media Action, Research and Learning<br>Chamberlain, Sara; BBC Media Action, BBC Media Action, India; BBC Media Action, Asia<br>Sharma, Manjula<br>Gharai, Dipanwita; Oxford Policy Management<br>Mishra, Bibha; Oxford Policy Management<br>Choudhury, Namrata; Jawaharlal Nehru University<br>Mohan, Diwakar; Johns Hopkins University Bloomberg School of Public Health<br>LeFevre, Amnesty; University of Cape Town
Keywords:	Public health < INFECTIOUS DISEASES, Health informatics < BIOTECHNOLOGY & BIOINFORMATICS, Information management < BIOTECHNOLOGY & BIOINFORMATICS, Information technology < BIOTECHNOLOGY & BIOINFORMATICS, HEALTH SERVICES ADMINISTRATION & MANAGEMENT

SCHOLARONE™
Manuscripts

I, the Submitting Author has the right to grant and does grant on behalf of all authors of the Work (as defined in the below author licence), an exclusive licence and/or a non-exclusive licence for contributions from authors who are: i) UK Crown employees; ii) where BMJ has agreed a CC-BY licence shall apply, and/or iii) in accordance with the terms applicable for US Federal Government officers or employees acting as part of their official duties; on a worldwide, perpetual, irrevocable, royalty-free basis to BMJ Publishing Group Ltd ("BMJ") its licensees and where the relevant Journal is co-owned by BMJ to the co-owners of the Journal, to publish the Work in this journal and any other BMJ products and to exploit all rights, as set out in our licence.

The Submitting Author accepts and understands that any supply made under these terms is made by BMJ to the Submitting Author unless you are acting as an employee on behalf of your employer or a postgraduate student of an affiliated institution which is paying any applicable article publishing charge ("APC") for Open Access articles. Where the Submitting Author wishes to make the Work available on an Open Access basis (and intends to pay the relevant APC), the terms of reuse of such Open Access shall be governed by a Creative Commons licence – details of these licences and which Creative Commons licence will apply to this Work are set out in our licence referred to above.

Other than as permitted in any relevant BMJ Author's Self Archiving Policies, I confirm this Work has not been accepted for publication elsewhere, is not being considered for publication elsewhere and does not duplicate material already published. I confirm all authors consent to publication of this Work and authorise the granting of this licence.

For peer review only

At the frontlines of digitization: The challenges and opportunities in maintaining accurate, complete, and timely digital health records in India's government health system

Authors: Kerry Scott[1], Osama Ummer[2], Sara Chamberlain[3], Manjula Sharma[4], Dipanwita Gharai[2], Bibha Mishra[2], Namrata Choudhury[5], Diwakar Mohan[1] and Amnesty LeFevre[1,6]

[1] Department of International Health, Johns Hopkins Bloomberg School of Public Health, 615 N. Wolfe St, Baltimore, Maryland, USA
[2] Oxford Policy Management-Delhi, 4/6 First Floor, Siri Fort Institutional Area, New Delhi, Delhi 110049, India
[3] BBC Media Action, India, Old Fort Saket District Mall, Saket District Centre, Sector 6, Pushp Vihar, New Delhi, Delhi 110017, India
[4] Independent researcher, New Delhi, India
[5] Centre for the Study of Law and Governance, Jawaharlal Nehru University
[6] School of Public Health and Family Medicine, University of Cape Town, Cape Town, South Africa

Corresponding author: Kerry Scott, 615 N. Wolfe St, Baltimore, MD, 21205, kerry.e.scott@gmail.com, 647-675-7956

For peer review only

Abstract

Objectives: To understand factors underpinning the quality of health information systems (HIS) data by examining the creation of government health records for pregnant and postpartum women in India.

Setting: Madhya Pradesh and Rajasthan states in India at the community, clinical and administrative levels of the health system.

Participants: Interviews (n=59) with frontline health workers (FLHWs), data entry operators, and higher level officials. Focus group discussions (n=12) with pregnant women to discuss experiences with sharing data in the health system. Observations (n=9) of interactions between stakeholders for data collection and the process of data digitization.

Primary and secondary outcome measures: Thematic analysis identified how key actors experienced the data collection and digitization process, reasons for late or inaccurate data, and mechanisms that can bolster timeliness and accuracy.

Results: Health system actors noted challenges with the digital system but valued the broader project of digitization. Pregnant women were comfortable sharing mobile numbers

with health workers but many were unaware that their data moved beyond their FLHW. FLHWs valued knowing up-to-date beneficiary mobile numbers but felt little incentive to ensure accuracy in the digital records system. Delays in registering pregnant women in the online portal were attributed to slow movement of paper records into the digital system and difficulties in gathering required documents from beneficiaries. Mobile numbers were hand written and copied multiple times by beneficiaries and health workers with variable literacy. Supervision tended to focus on completeness rather than accuracy.

Conclusions: Frontline health workers have adjusted to a digital HIS despite implementation challenges and are enthusiastic about moving digital data entry from the facility to the field-based worker. However, government health systems may consider increasing focus on data processes, training and supervision that prioritizes accuracy and timeliness.

For peer review only

Strengths and limitations of this study

- This study was strengthened by the use of multiple research methods (interviews, focus groups and observation) and engagement of multiple frontline stakeholders: the women whose data is entered into the system, the frontline health workers who care for them and create the initial (paper) records, the data entry operators who digitize these records, and the managers, administrators and leaders who supervise these processes.
- Frontline health workers and data entry operators were aware that their work was being observed and thus may have minimized or hidden unauthorized behavior, such as shortcuts to speed up data entry or unsanctioned task-shifting.
- Data collection occurred just prior to the launch of a new digitization strategy in some (but not all) regions, wherein frontline health workers would be given tablets and asked to directly digitize patient health data; thus some of the challenges identified in this study may have self-resolved due to this new (shorter) dataflow pathway, and new challenges to accuracy, timeliness and completeness may have arisen.

Introduction

Health information systems (HIS) capture data on patients and their contacts with routine health services. HIS support responsive policymaking, resource allocation, routine service delivery, and health systems accountability.[1] The achievement of these functions is largely dependent on the accuracy, completeness and timeliness of HIS data.[2] Information communication technologies (ICTs) are increasingly being used to improve the core functions of HIS through digitization.[3–5] While ICTs for HIS have great potential to strengthen

For peer review only - http://bmjopen.bmj.com/site/about/guidelines.xhtml

health systems[6,7], the use of digital tools can place a high burden on health care providers who require device and software training, technical support, and reliable electricity, internet, and servers.[8]

The digitization of HIS has occurred concurrently with increasing mobile phone penetration at a population level and, in turn, an increase in mobile health (mHealth) initiatives. mHealth programs can transmit health-related information, send reminders, or solicit patient feedback on services.[9] To attain high population level coverage at scale, mHealth programs often rely on beneficiary mobile numbers and other data that have been collected through government HIS. The accuracy, completeness, and timeliness of records in government systems can directly affect the capacity of mHealth programs to reach target populations at the right timepoints.

The Government of India's Kilkari program is the world's largest maternal mobile messaging initiative. It delivers maternal, child, and reproductive health information content through up to 72 once-weekly outgoing pre-recorded calls. Pregnant or postpartum women are subscribed to Kilkari based on the mobile number captured in governmental HIS registries called, depending on the state, the Maternal and Child Health Tracking System (MCTS) or Reproductive and Child Health (RCH) system. The gestational age estimate or child's date of birth is used to determine the appropriate Kilkari messages to send based on the stage in pregnancy or child's age. Missing, incorrect or late data limits the reach and appropriateness of program timing.

Quantitative research has identified gaps in MCTS/RCH data. A 2015 study[10] found that one-third of women's profiles in Rajasthan and two-thirds in Uttar Pradesh were incomplete. In addition, one quarter of the unique mobile numbers registered for Kilkari are never reached due to inaccurate or out of date mobile numbers or the phone being switched off or out of network. Data delays impede overall exposure to the program. Delays in registering pregnant women in the online portals averaged 72 days between service delivery and data capture in Bihar's MCTS.[11] Kilkari exposure can begin in second trimester of pregnancy, however only 31% of subscribers answered their first call during this period.[12] Over half (58%) answered their first call after they give birth, which means they missed at least one-third of Kilkari's content.[12]

This analysis aims to identify barriers to the accurate, complete and timely creation of MCTS and RCH HIS records in India. We explore beneficiary perceptions of providing mobile phone numbers to health care providers, and frontline health worker (FLHW) experiences with the digital health record system. Study findings will inform health systems in India and beyond as they move towards ICT-enabled strategies to bolster the quality of health information systems.

Methods

Study setting

This qualitative study took place in Madhya Pradesh and Rajasthan, two large Hindi speaking states in central and western India, respectively. These states have high burdens of

For peer review only

maternal and child mortality, significant gender gaps around technology access and literacy, and sub-optimal maternal healthcare services (table 1).

Table 1. Social and health indicators, Rajasthan and Madhya Pradesh

Indicator	Rajasthan	Madhya Pradesh
Population	77 million	82 million
Maternal mortality ratio (deaths per 100,000)[13]	164	173
Under five mortality (deaths per 1000 live births) [14,15]	51	65
Literacy[14,15]		
• Female	57%	59%
• Male	85%	82%
Mobile phone access[14,15]		
• Household ownership	94%	84%
• Female access	41%	29%
Maternal health care[14,15]		
• Pregnant women attended ANC in first trimester	63%	53%
• Received recommended four ANC visits	39%	36%
• Gave birth in a health facility	84%	81%
• Received postnatal health check within 2 days of birth	64%	55%
• Registered pregnancies for which the mother received Mother and Child Protection (MCP) card	92%	92%

Both Madhya Pradesh and Rajasthan moved to digital health records in the late 2000s, however there are important differences between the states, in terms of the digital programs and processes implemented (Table 2). In 2016, MP transitioned from MCTS to RCH. RCH added urban coverage, an initial registration of all "eligible couples" (married couples of reproductive age) who would then be linked to pregnancy tracking when a pregnancy occurred, and the creation of village profiles.[16] Furthermore, RCH expanded the data elements collected from 111 (in MCTS) to 247 to include abortion tracking, beneficiary bank account and identification details (including Aadhaar number), additional details about the pregnant woman's antenatal care, infant feeding practice, and the child's immunization records for his or her first five years.[16] Mobile phone numbers were collected in MCTS and continued to be in RCH. In contrast to MP, the Rajasthan state government did not adopt RCH but instead retained its state-level version of MCTS, called the Pregnancy, Child Tracking, and Health Services Management System (PCTS), which syncs with MCTS.

Table 2. Comparing Rajasthan and Madhya Pradesh's digital HIS

Parameter	Madhya Pradesh	Rajasthan
HIS system currently in use	Reproductive and Child Health (RCH) (an expanded and upgraded version of MCTS)	Pregnancy Child Tracking and Health Services Management System (PCTS)
Timeline	Adopted MCTS in 2009 and RCH in 2016	Adopted PCTS in 2008 (first state in India to launch an electronic health records system)
System through	Through separate portals, women get JSY (Rs. 1400) for all institutional	Online JSY and eShubh Laxmi Payment and Management (OJSPM): Through the

For peer review only - http://bmjopen.bmj.com/site/about/guidelines.xhtml

Parameter	Madhya Pradesh	Rajasthan
which pregnant and postpartum women receive financial support	deliveries, Mukya Mantri Shramik Seva (Prasuti Sahayata) Yojna (Rs. 16,000) for labourers, and Pradhan Mantri Matritva Vandanada Yojina (PMMVY) (Rs. 5000) for first pregnancy	Bhamashah program, women get JSY payment (Rs. 1400) and Shubh Laxmi (Rs. 7300) payment for girl child
State-level ID number	Samagra, mandatory unique ID for families and individuals	Bhamashah, ID for the state health insurance program
Who collects HIS data at the frontline?	ASHA & ANM	ASHA Sahyogini, Anganwadi worker & ANM
Feedback mechanisms built into the digital record system	Creates a workplan for the ANM telling her who is due for ANC, delivery, and PNC, as well as listing drop outs; sends SMS alerts to beneficiaries and also to health functionaries at different levels	Creates a workplan for the ANM telling her who is due for ANC, delivery, PNC as well as listing drop outs; sends SMS alerts to beneficiaries (Swasthya Sandesh Sewa)
Paper forms involved	***1) RCH form***: One form per woman, filled by ASHA/ANM and given to DEO. Meant to travel back and forth between ANM and DEO as the woman proceeds through her ANC visits and delivery.<br>***2) Mother Child Protection (MCP) card***: Card filled by ANM/ASHA; stays with the pregnant woman<br>***3) RCH register:*** Book kept with the ANM or at the health center; all pregnancies under the ANM are in this book. It is passed to the DEO for entry.<br>***4) Personal ANM diary***: Unofficial paper book that ANMs fill (because RCH register is sometimes too big to bring to the village or is with the DEO)<br>***5) Personal ASHA diary***: Official paper book that the ASHA fills	***1) Pregnant women's ANC registry form***: Paper where up to five new pregnancies can be listed with details of first ANC. This form is filled in by the ASHA/ANM and passed to the DEO for entry into PCTS.<br>***2) Mamta card***: Card filled by ANM/ASHA; stays with the pregnant woman<br>***3) RCH register***: Book kept with the ANM or at the health center; all pregnancies under the ANM are in this book. It is passed to the DEO for entry.<br>***4) Personal ANM diary***: Unofficial paper book that ANMs fill (because RCH register is sometimes too big to bring to the village or is with the DEO)<br>***5) Personal ASHA diary***: Official paper book that the ASHA fills

The states thus provide a natural comparative study between a more typical case (Madhya Pradesh), which, like most large Indian states, recently moved from MCTS to RCH, and an outlier case (Rajasthan) which has retained a tailor-made electronic record system since the beginning of digitization.

Data collection

Five experienced qualitative researchers with master's level social science degrees (authors OU (male) MS, DG, BM and NC (female)) conducted in depth interviews (n=59), focus groups (n=12) and observation of data collection and entry (n=9) in Rajasthan and Madhya Pradesh in September 2018 (table 3). All researchers were trained over a one-week period, which

For peer review only - http://bmjopen.bmj.com/site/about/guidelines.xhtml

included pilot testing the FLHW interview guide. In each state, we selected one district and two blocks within that district with high levels of female phone ownership to explore barriers to capturing women's mobile phone numbers in MCTS/RCH. We sampled government health system actors who were involved in MCTS/RCH at the state, district, block and frontline levels, including medical officers, data entry officers and frontline providers, as well as with women who recently interacted with government healthcare providers in situations where they were asked to register their mobile numbers in MCTS/RCH (table 3).

Table 3. Respondent sample

Respondent type	Respondent profile	MP	RJN	N
In depth interviews				
State level stakeholders	Senior government employees of the department of health and family welfare who are in charge of data	3	1	4
District stakeholders	Community mobilizers, District Programme Manager (DPM), District Community Mobilizer (DCM), District Monitoring and Evaluation Officer (DMEO), District Nodal Officer (DNO)	3	4	7
Medical officers (MO) at primary health centers	Doctors (allopathic, homeopathic or ayurvedic), 5.5 years training	2	4	6
Data entry operators	Information technologists with undergraduate level education	4	6	10
Auxiliary nurse midwives (ANMs)	Female maternal and child health worker with 1.5 years training (6 months midwifery focused)	10	9	19
ASHA	Female volunteer community health worker, received incentive based remuneration, initial 24 days training and periodic week-long additional training	3	5	8
Other stakeholders at the PHC and block level	Block Programme Manager (BPM), Multipurpose Health Supervisor (MPS), Primary Health Centre Supervisor (PHS)	2	3	5
Total IDIs		27	32	59
FGDs				
Beneficiaries	Pregnant and post-partum women with mobile phones who recently interacted with health system actors for MCTS/RCH registration	6	6	12
Observation				
Beneficiary – FLHW interaction	Observation of beneficiary – FLHW interaction (ANC or post-partum) interaction where MCTS/RCH data recorded	1	1	2
Data entry into electronic system	Observation of data entry into electronic system	3	4	7
Total observations		4	5	9

For peer review only

The respondents were approached through their government health facility. A research team member contacted potential respondents by phone or face-to-face and explained the study, and that the team was from a Delhi-based company and had governmental approval, then invited them for a face to face meeting to learn more and, if they agreed, to participate. The study information and informed consent was read to each potential participant and then summarized in conversational language to ensure comprehension. While all the health facility staff approached for the study agreed to participate while three women invited to attend focus groups declined, siting responsibilities at home. The interviews took about an hour and the focus group discussions took about an hour and a half; all were conducted in health faculties and audio recorded and detailed notes were taken. When curious onlookers came over during focus groups and interviews, another

For peer review only - http://bmjopen.bmj.com/site/about/guidelines.xhtml

researcher politely asked them to move on. If any supervisors, patients or family members stopped by to speak to the respondents during the interviews or focus groups the research paused until privacy was restored.

Interviews and FGDs were conducted using semi structured guides that explored a range of domains around sharing, documenting, inputting, and using health data, such as reasons for late pregnancy identification by FLHWs, FLHW work environment, and a detailed description of data flow from when a pregnant woman first interacts with a FLHW until her health information is entered into the online portal.

Ethics approval

This study was approved by the institutional ethics review boards at Sigma, Delhi, India (10041/IRB/D/17-18) and JHU, Baltimore, USA (00008360). All respondents provided informed oral consent.

Data analysis

Daily debriefs enabled the team to share emergent findings, refine the focus of their probing for the next day's data collection, and identify areas of saturation. The audio files were transcribed and translated into English. Data were coded in Dedoose by OU and KS, using principles of thematic network analysis.[17] A coding framework was developed that consisted of emergent codes on specific reasons for inaccurate and delayed data, that were then grouped according to an overarching data flow framework (Fig 1). For instance, we created a code cluster for late ANC registration, which included codes to be applied to text describing when and how pregnancies come to the FLHW's attention, when pregnancies were entered into the online portal, reasons FLHWs become aware of pregnancies late (after the first trimester), and the implications of a woman's choice of the public or private sector for ANC on timeliness of registration. After coding, we read the text excerpts that had been tagged for specific codes to identify how key actors experience of the data collection and digitization process, reasons for late or inaccurate data, and mechanisms that can bolster timeliness and accuracy.

Conceptual framework

We describe the data collection and digitization process according to a general health system data flow framework (Fig 1). This framework identifies six components that enable the creation and movement of health data from beneficiaries through FLHWs to electronic data entry and onward.

Figure 1. Data flow framework for electronic health record systems

The first component, "beneficiary," describes women's access to the data required for documentation, particularly mobile phone numbers, as well as their attitudes towards sharing this data with government health system functionaries. The second component considers the beneficiary-FLHW interactions that initiate the government health system's

For peer review only - http://bmjopen.bmj.com/site/about/guidelines.xhtml

awareness of a health event. In this section we consider how and when FLHWs become aware of a new pregnancy, the value that FLHWs place on collecting beneficiary data, and the strategies used by beneficiaries to collect data. The third component involves the creation of initial paper records. In this section we consider the various official and unofficial forms and registers where data is recorded, potential delays between a health worker interacting with a beneficiary and a paper record being created, and the FLHW experience of documentation. The fourth component considers the process through which a paper health record reaches the site of digitization. While steps three and four may be dropped as FLHWs directly create digital records themselves, in most LMIC health systems paper records created during outreach service provision remain the norm. The fifth component is the time when data is digitized and considers the data entry personnel's work environment, training, the electronic portal interface, and staffing considerations. The final component examines the ongoing use of data through paper forms and online systems. Here we consider how data can be corrected or updated, data monitoring and supervision, and the use of data by FLHWs and higher level health system actors.

Patient and Public Involvement

The research was shaped by health system actor priorities, experiences and preferences through iterative probing and flexibility within our research domains. Results were disseminated to Government of India stakeholders but not to research participants.

Findings

Our findings are organized conceptually according to the six components in the general data flow framework. For each step, we provide a description of how this aspect of the data system is experienced by the actors involved, and identify barriers and facilitators to timeliness and accuracy.

Level 1. Beneficiary

Women face barriers to accessing phones and services that generate the data required by MCTS/RCH

Women who did not have their own personal mobile phones could not provide their number. Many women lived in households with phones so could provide their husband's phone number or the number for a shared phone. Women living in households without a phone could provide a neighbor's phone number or a FLHW's number.

> R: Some women have their husband's mobile number. In some cases both husband and wife don't have mobile phones. Some are from poor families where both of them don't have mobile phones.
> I : So, what do you do in such situation?

For peer review only - http://bmjopen.bmj.com/site/about/guidelines.xhtml

> R: Either we take a relative's phone number or a neighbor's. […] If any laborer is there who does labor work how will he have mobile phone? Even if it's there then her husband must have taken it while going for labor work. (MP_IDI_FLHW_06)

Additional data required from beneficiaries included their bank account numbers and branch names (for both the beneficiary and her husband), national identification (Aadhaar), ration card number, and state identification numbers (Bhamashah state health insurance in Rajasthan and Samagra in Madhya Pradesh). FLHWs explained that women often did not have all of these items or did not know this information. ASHAs were tasked with following up with women to encourage them – or at times personally help them – open bank accounts, register for identification numbers, and provide photocopies of this information.

> Like the [bank] account number, aadhaar number we get them very rarely. We have to with the ASHA then go again to get information. We fill the form and submit it and it's fed in. If half the information remains incomplete then we can't do the RCH. (MP_IDI_FLHW_01)

Women are generally comfortable sharing data with frontline health workers

Women and FLHWs overwhelmingly reported that beneficiaries with access to a mobile phone shared their mobile number with their ASHA or ANM without hesitation and without questioning. They explained that women trusted FLHWs and felt that the number would only be used to help them, such as to convey important information about health services or financial benefits through government programs.

> They are observing me from 20 years so they are aware that madam will not misuse the number so they never deny giving number (RJN_IDI_FLHW_25)

> [They take our mobile number] so that they can give us a phone call next time to call us whenever vaccination is to be given. That's why. (RJN_FGD_BENEF_07)

> They are aware that the mobile number is essential as when the money will come in the bank account then they will get the message on mobile. We take the mobile number for their comfort only as we can call them as required. […] And calls also come on their mobile who tells them about the health. (RJN_IDI_FLHW_27)

There were exceptions to this predominantly positive view of sharing mobile numbers with health workers. FLHWs and beneficiaries reported that a very small minority of women resisted sharing their numbers with them because of concern about receiving calls from strangers or because they had hidden their mobile phone.

In Madhya Pradesh, most women thought that only the ASHA had their number. When told that their numbers were passed onward from the ASHA to the ANM, women were unconcerned. In Rajasthan, women were generally aware that their mobile numbers were collected for use beyond beneficiary-ASHA communication. They clearly explained that their mobile number was linked to their receipt of financial benefits. While most women in Rajasthan said the only calls they received from government actors were from their ASHA,

For peer review only - http://bmjopen.bmj.com/site/about/guidelines.xhtml

calling them for checkups, a few received informational calls from the government of India, which were likely Kilkari messages and one said that the government would use their mobile numbers to call them and check “whether the facilities which government is providing are reaching us or not” (RJN_FGD_BENEF_09).

Level 2. FLHW-beneficiary interaction

Early identification of most pregnancies – with notable exceptions

Digital records of pregnancies can only be created after the government health system functionaries identify a pregnant woman. ASHAs are a pregnant women’s first point of contact with the government health system. ANMs rely on ASHAs to identify pregnant women and encourage them to meet the ANM for antenatal care. Beneficiaries and FLHWs reported that ASHAs were notified within the first four months about almost all pregnancies.

However, FLHWs noted that women living in remote communities, migrants and less educated women, may delay seeking antenatal care due to “carelessness” (RJN_IDI_FLHW_27), superstition, and not knowing that they were pregnant. Wealthier women who received ANC in private facilities would also be missed by the government health system or registered only after giving birth.

FLHWs place high value on collecting and digitizing beneficiary data

FLHWs in Rajasthan and MP articulated a clear focus on accurately collecting and digitizing women’s bank account details and identification numbers, since this data enabled women to receive financial support from government programs. While phone numbers were considered important for ASHA-beneficiary communication, the value of digitizing this information (rather than just ensuring it was collected in the ASHA’s paper records) was not always clear. Some health workers in MP could only articulate a vague sense that entering mobile numbers in RCH was useful. Most FLHWs in MP reported that mobile numbers were used by the state or central government to call beneficiaries and check whether they are receiving appropriate health services, suggesting that FLHWs saw the digitization of women’s mobile numbers as a means for the government to keep tabs on FLHW performance. In Rajasthan, FLHWs were clear about the value of mobile numbers in PCTS, and explained that the numbers were not only used for checking that women were receiving health services, but also for conveying health information, advertising government programs, and notifying women about financial transfers.

FLHWs describe several strategies for trying to collect accurate data

In Rajasthan and MP, ASHAs and ANMs explained that many women could not accurately recall their mobile numbers at ANC visits. For cases when women did not have mobile phones with them, ASHAs would visit their homes and ask other family members to provide a mobile number or ASHAs and ANMs would ask women to bring a mobile number written on a piece of paper to the next ANC checkup.

For peer review only - http://bmjopen.bmj.com/site/about/guidelines.xhtml

> Like, they don't know, some of them.. they don't know the mobile number. So, we tell them to ask the people in her house who can write, to note it down on a piece of paper, and bring it to us. Tell them the sister needs it, for your information. So they do that. (MP_FLHW_01)

ASHAs were confident that they received accurate numbers because they reported frequently calling women using the number provided. In cases when women had mobile phones with them, some ANMs or ASHAs reported giving themselves a missed call from beneficiary mobile phones to identify the number; however, the use of missed calls was not a universal practice.

FLHWs said that pregnant women sometimes asked FLHWs to record their husband's mobile number rather than their own, even when she had her own phone. Women in one FGD (RJN_FGD_BENEF_10) explained that the husband's mobile number was often provided so that he would receive the SMS notification of a financial transfer into the beneficiary's bank account. While some FLHWs complied in taking the husband's number, others tried to convince woman to provide her own mobile number since her husband will frequently be out of the house with the mobile.

FLHWs reported asking women to provide photocopies of bank account information and government identification cards, in order to reduce errors that could be introduced by women conveying this information on scraps of paper. When assessing last menstrual period (LMP) in order to estimate gestational age, two FLHWs noted that many women struggled to report exact dates. FLHWs estimated LMP based on women's recall of the moon cycle, harvest, or festivals, which would introduce inaccuracy around pregnancy stage.

Level 3. Creation of paper records

Beneficiary data copied by ASHAs and ANMs across multiple paper forms and registers

In MP and Rajasthan, when an ASHA identified a pregnancy in the village she would write key information about the woman in her official ASHA diary. In cases where ASHAs were considered sufficiently literate by the local ANM, the ASHA was also expected to start a paper RCH form (in MP) or ANC form (in Rajasthan) by filling in some initial fields. In MP we found that ASHAs in one block reported being paid Rs. 50 ($0.80) for each form they created, while in a nearby block ASHAs received no payment. In both states, some ASHAs first wrote the pregnant woman's details in an unofficial notebook and then copied this information into the official ASHA diary and the RCH/ANC form. While some ASHAs were clear and confident about their role filling in the RCH/ANC form, others were unsure about which documents they completed.

When the woman comes the VHND, the ASHA will pass the partially-filled RCH/ANC form to the ANM. The ANM will fill in additional details from the woman's first ANC visit, often with the ASHA's help. ANMs explained that they were overwhelmed by documentation

For peer review only - http://bmjopen.bmj.com/site/about/guidelines.xhtml

requirements and thus had to take assistance from ASHAs, even when ASHAs struggled with the literacy and numeracy requirements of this work.

> We have to fill a big register, we make sehyogini [ASHA] fill it that is the reason it gets wrong. Now they [ASHAs] don't understand. They themselves have filled the card. Now, there was so much crowd that time, that I was writing something.. and if they say, the weight is 53 and she says its 61, so then there's confusion like that. So sometimes they write it wrong. When there's a crowd sometimes, we take help from them only, and it becomes wrong. (MP_IDI_FLHW_07, ANM)

In addition to the RCH/ANC form, ANMs record data about pregnant women in the ANM RCH register (a book) and create a Mother and Child Protection (MCP) card (Madhya Pradesh) or Mamta card (Rajasthan). MCP/Mamta card is given to the pregnant woman to keep. Similar to our observations of ASHAs, ANMs in MP also sometimes wrote down beneficiary information in unofficial notebooks and then copied this information into their official RCH register and the RCH forms once they returned home; they explained that the RCH register is heavy and they wanted to keep it neat and clean. In Rajasthan the official register was smaller and more portable so ANMs tended to carry it with them.

Delays in antenatal care have implications for the creation of health records

There could be several weeks or even months between the ASHA becoming aware of a pregnancy and the ANM meeting the woman for first antenatal care. No paper record could be completed until the woman interacted with an ANM for antenatal care, thus introducing delay in the production of official health records.

FLHWs found data collection under RCH to be too time-intensive

ANMs in Madhya Pradesh said that they found data collection to be highly burdensome. They felt that their work had shifted from healthcare provision to finding documents and filling in forms. FLHWs described facing pressure to complete the digital records from senior actors in the health system hierarchy as well as from beneficiaries who were worried about delays in receiving their financial incentives. They described having to fill up to 70 data fields in various paper forms and registers, and having to track down beneficiary banking and identification details, without adequate time or support.

> We have to do this also and that also. In this what happens is, like this "Prashuti Sahayata Yojana" has come [i.e., yet another scheme has been introduced]. So, we keep on calling them the whole day that "Get this paper" "This is not enough" "This paper is short." Just now also madam [a supervisor] called. And sir [another supervisor] also called saying that high risk pregnancy too needs to be checked too. (MP_FLHW_06, ANM)

> They've increased our work a lot, a lot. … We need to visit each beneficiary 10 times… "Give the Aadhar card, the Samagrah ID." The beneficiary isn't able to submit, isn't able to give it to us, and above that they reach here and start complaining, that, madam isn't filling our form so we aren't getting our money when they themselves aren't giving us the documents all together. We used to go to each house, of the beneficiary, give them the vaccinations, meet each one. And the bai [women] that were there, they had very good behaviour, were

For peer review only - http://bmjopen.bmj.com/site/about/guidelines.xhtml

For peer review only

> respectful, and would make us sit inside, that come, madam, sit inside. Would treat us very well. And, these days I don't know what is happening, so the beneficiaries also snap at us, for a little bit. If they say, madam we need the money, and we tell them to submit the documents, and they'll get the money... Oh oh, but you need this paper, and that paper, and we've given it to the Anganwadi, to you, and to the ASHA, what is this nonsense. (MP_IDI_FLHW_07)

ANMs who worked with low-literacy ASHAs noted that they faced an additional struggle because they ould not rely on the ASHAs to complete any paper forms.

Level 4. Paper records brought to health centre

Delay between creation of paper records and records reaching the health center with a computer

The ANM becomes aware of a pregnancy during the woman's first ANC visit and considers the woman's pregnancy to have been registered at this time. However, the pregnancy is not entered into the online portal until the ANM brings the RCH/ANC forms and RCH registers to the health center for digitization. This occurs only once per month. In addition, if an ANM is waiting for a beneficiary to provide documents required to complete the RCH/ANC form, she will hold on the form even longer, until a future visit to the health center.

> There are some women in the village, even after their delivery, their Samagra ID isn't able to be made..[...] [So] It's not fed [entered into RCH] here because the Samagra ID is incomplete. (MP_IDI_FLHW_01)

Level 5. Data entry

DEOs spoke of high workloads and some technical challenges but noted recent improvements and supportive peers

DEOs in both states said there was excess workload, which could lead to data entry backlog. They explained that in addition to entering maternal and child health data they were also responsible for entering data from the pharmacy, ASHA records, birth and death registration, HMIS, and health facility forms. In some areas, one DEO was covering the workload of two or three because of vacancies. ANMs suggested that DEOs may make data entry errors, because they were rushing or careless.

In both states the DEOs mentioned that the infrastructure to support them was improving, such as the provision of newer computers, faster internet, and upgraded servers. In Rajasthan the DEOs had battery backup for power outages and a dedicated broadband internet connection; even so, during observation, opening the PCTS portal took up to 10 minutes because of slow internet connection or server capacity. In MP, many DEOs did not have functioning battery backup, leaving them unable to work when the electricity went off.

For peer review only - http://bmjopen.bmj.com/site/about/guidelines.xhtml

Some relied on their own portable WIFI device or their mobile phone's hotspot, both of which were often slow.

DEOs in MP reported having received only one training on RCH, while DEOs in Rajasthan received frequent trainings and felt reasonably well supported by the supervisory structure as well as their peers. The one-time training in MP occurred years ago, and many months before RCH was introduced so recipients forgot much of it. It consisted of a presentation without any hands-on learning. DEOs reported learning how to use RCH on the job, through trial and error and from other DEOs, who provided ongoing peer support.

Required fields and auto-fill invite errors

The digital portals had numerous required fields, validation checks, and autofill features, including that 10 digits be entered in the mobile number field. In many cases these features supported accurate and complete data entry. However, DEOs recalled instances where these features forced them to introduce errors into the system. For instance, when DEOs encountered forms that did not have a beneficiary mobile number, DEOs reported using the ASHA's number or their own number because the mobile number field is mandatory. In MP, DEOs reported that RCH initially required all mobile numbers to start with the digit 9. When a new phone company began issuing mobile numbers beginning with 6 or 7, DEOs could not get the system to accept these numbers until the RCH portal was updated.

In RCH, pregnancy records are linked to eligible couple record. The pre-existing eligible couple registry auto-completes fields in the new pregnancy registry, including for the mobile number. Thus the phone number provided by a family at time of marriage becomes the default current phone number at the time of pregnancy.

DEO and ANM absences can lead to data entry delays and may compromise quality

When DEOs took time off, there were not always additional trained personnel to fill in. Other health system actors (such as a PHC health supervisor or ASHA supervisor) would take over data entry, even without training. In cases of longer-term absence, such as when there was no DEO appointed to a PHC at all, a DEO at a neighbouring facility would be assigned to cover both catchment areas. ANMs then had to bring their paper records to a more distant health facility to have them digitized and DEOs had to cover double the caseload.

There are gaps in data collection at the frontlines when ANMs take time off, during transition from an older ANM to a new recruit, or when the ANM position is vacant. During these periods no health records are created or digitized but the district health system actors demand that data continues to be entered. This leaves the DEO in a difficult situation wherein no paper records are being passed on to them but they are expected to create digital records.

Level 6. Ongoing use of paper and online records

For peer review only - http://bmjopen.bmj.com/site/about/guidelines.xhtml

Mobile numbers are not corrected or updated in the online system

Beneficiaries explained that they updated their ASHA whenever they changed mobile numbers and ASHAs were confident that they retained up to date mobile numbers for the pregnant women in their villages. However, ASHAs, ANMs and DEOs reported that they do not update or change mobile numbers in the online portals. Some DEOs were unsure if it is even possible to do so. Checking and correcting bank account details and government identification details was common, since this ensured financial transfers proceeded. Ensuring correct and up-to-date mobile numbers was not a priority. Even among women and FLHWs who identified benefits for women who shared their mobile number with the government, the value of receiving government information and notifications was overshadowed by the importance of receiving financial incentives.

Supervision focuses on completeness not accuracy

DEOs as well as block, district and state level actors focused on data entry completion and timeliness but not on accuracy. DEOs were encouraged to complete their data entry activities each month and clear backlog. However attention to data accuracy only arose when mistakes came to light through irregular means, such as a financial transfer going into the wrong bank account or a polio outbreak alert being triggered by a DEO incorrectly entering eight polio cases rather than zero.

In MP, some health facilities had multi-purpose supervisors and ASHA support staff who checked the forms and registers provided by ANMs and ASHAs to the DEO and who oversaw the DEOs work. These supervisors could flag potential inaccuracies for follow up. However in other facilities these positions were vacant. The Medical Officer was expected to sign off on the DEO's work but was so busy with patient care that they often gave an automatic approval or even provided their login and password to the DEO so that the DEO could approve their own work.

In both MP and Rajasthan, DEO work at the health centers was overseen by block level supervisors. In both states these actors reported being overwhelmed by having to oversee five to seven software programs and more than 50 reporting processes and could only focus on issues of incomplete data.

At the frontlines, ANMs and ASHAs in MP and RJN had no way of knowing whether the mobile number entered in PCTS or RCH was correct and had never been asked to cross check these digital records with beneficiary numbers.

Health system actors have an overall positive view of the value of digitization

Despite noting hiccups and increased documentation burden, ANMs and ASHAs were generally positive about the move towards digital health records. They framed the use of digital systems as a better way for them to showcase their hard work and to receive recognition (and, for ASHAs, performance based remuneration), rather than a way to benefit from the digital system's ability to generate ANM workplans or other synthesized data. Block, district and state level actors described benefiting enormously from the digital system. The explained that it enabled them to see the progress of programs, monitor

For peer review only - http://bmjopen.bmj.com/site/about/guidelines.xhtml

service provision by FLHWs, conduct yearly planning to address gaps, and prioritize resources: “In one click the full report can be retrieved” (RJN_IDI_MO_04).

Discussion

This study explored how beneficiaries, frontline health workers, data entry operators and higher level actors in the government health system experienced HIS digitization to understand barriers to timely, accurate, and complete data (Table 4). We found that frontline workers and their supervisors prioritized the accuracy of core health information and of data elements that enabled pregnant clients to receive government financial entitlements (e.g., bank account information, identification numbers) over timeliness or the accuracy of beneficiary mobile phone numbers. Providers reported that it often took months – and significant effort that detracted from healthcare provision – to gather the documents and information from pregnant clients required to create a digital record; frontline workers relied on their existing paper systems to provide ongoing healthcare and keep track of up-to-date beneficiary mobile numbers.

For peer review only

For peer review only - http://bmjopen.bmj.com/site/about/guidelines.xhtml

Table 4. Summary of barriers to the creation of timely, accurate and complete PCTS/RCH data

	Barriers to timely data	Barriers to accurate and complete data
1. Beneficiary	• Beneficiaries must open bank accounts and attain government identification before an electronic record can be created	• Duplicate mobile phone entries are created when one number is provided by multiple pregnant women, such as when multiple women in a joint family with one phone become pregnant, or when multiple women provide a neighbour's number or the ASHA's number.<br>• Non-beneficiary numbers are entered when women without personal mobile phones provide their husband's number, a joint family phone number, a neighbour's number, or the ASHA's number.
2. Beneficiary-FLHW	• While ASHAs learn of most pregnancies within the first trimester, pregnancies among migrants, women living in remote communities, very poor women who did not see the value in seeking early ANC, and wealthy women who received ANC in the private sector were all detected late – sometimes even after the baby was born	• FLHWs value collecting correct mobile numbers so that ASHAs can keep in touch with beneficiaries. The value of accurately digitizing these numbers is not always clear to FLHWs in MP. In both Rajasthan and MP accurate mobile phone number digitization does not have immediate or direct influence on health care or financial transfers.<br>• While ASHAs and ANMs have a number of strategies to seek correct mobile numbers, beneficiaries may incorrectly recall their mobile numbers; checking the beneficiary's number through a missed call is common but not universal and is not an official requirement.<br>• Accurate gestational age estimation (which is linked to the provision of stage-based information in Kilkari) is a challenge since women cannot always report the exact date of their last menstrual period.
3. FLHW creates paper records	• There can be a delay of weeks or even months between an ASHA learning of a new pregnancy and the woman receiving her first ANC from the ANM at village health and nutrition days<br>• It is only after the woman meets the ANM that her (paper) RCH form can be passed onward for digitization	• Copying errors can occur when ASHAs and ANMs write women's information in multiple places for different programs, often with slightly different data fields; beneficiaries may also provide their mobile phone numbers on scraps of paper.<br>• ASHAs are often expected to complete many fields in the RCH/ANC form before the ANM fills in additional details and passes it to the DEO; ASHAs with lower literacy struggled with this responsibility.<br>• FLWHs in MP found data collection for RCH to be highly burdensome and described high pressure from above to fill forms and registries without adequate time or support.<br>• Some ASHAs were confused about the names and purposes of various paper forms and some hand made their own additional registries that they use in the field
4. FLHW brings paper records to health centre	• Many ANMs bring paper RCH/ANC forms and RCH registers to the health center for data entry only once a month<br>• A woman's paper form cannot be passed to the health center until she has provided all required fields (such as	• Respondents did not note any risks to data accuracy or completeness while transporting paper forms and registers from the field to the data digitization facility.

For peer review only - http://bmjopen.bmj.com/site/about/guidelines.xhtml

	Barriers to timely data	Barriers to accurate and complete data
	a bank account number), which may introduce further delays	
5. Data entry into online portal	• Server errors, internet and electricity issues may delay DEO in creating electronic records<br>• If DEOs are overburdened with data entry activities, they can fall behind	• When entering a pregnancy into the online portal, RCH auto-fills the mobile number provided at the time of "eligible couple" registration; this number may be outdated.<br>• DEOs in MP found their training on RCH to be insufficient; peer support enabled DEOs to navigate challenges. DEOs in Rajasthan were confident in PCTS and received frequent training.<br>• When DEOs are on leave or when DEO positions are vacant result in other health facility employees, who have not been trained,
6. Ongoing use of paper and online records	None identified	• Although the ASHA is generally notified if a pregnant woman's contact number changes over the course of her pregnancy, this new number will not be updated in the PCTS/RHC portals<br>• Supervisor positions remain vacant in some health facilities and higher level supervisors (block and district level) oversee numerous databases and reporting systems;<br>• Supervisors tend to focus on completeness and timeliness rather than accuracy

For peer review only

For peer review only - http://bmjopen.bmj.com/site/about/guidelines.xhtml

Scholars have critiqued India's health system digitization as disproportionately focused on the collection of data rather than its use.[18,19] However the health system actors that we interviewed conveyed a clear understanding of ways in which the digital HIS supports work planning and monitoring. Nonetheless, the overarching logic behind digitization articulated by frontline workers remained as a mechanism to feed information up the health system hierarchy rather than to improve their work planning or to access information about their clients.

While pregnant women were willing to share their or their family's mobile numbers with ASHAs and ANMs, many were unaware that their mobile numbers were passed onward and entered into computers. Although women trusted the health workers and associated the provision of requested data with receiving benefits from the government, strengthening consent processes can protect beneficiaries and safeguard relationships at the frontlines.

As identified earlier by other researchers,[20] we again found poor standardization across multiple data systems (paper based and digital) and identified opportunities for streamlining records and entry options to become more intuitive and user friendly. Echoing findings from other settings,[21] supervision focused on completeness rather than accuracy, highlighted by the fact that there were no mechanisms to check whether mobile numbers were correct. Workers at the frontlines require clear guidelines on how to handle common challenges in accessing data, particularly mobile phone numbers, including how to handle data changes (such as new phone numbers) and unavailable data (such as no phone number).

Required fields, auto-fill features and validation checks can force DEOs to adjust the data provided to them according to their judgement in order to proceed with data entry. Another study on PCTS in Rajasthan found that the portal would not accept delivery dates that appeared to occur after more than 9 months gestation, a situation created by incorrect gestational age estimation based on faulty last menstrual period estimates.[22] Finally, the consolidation and standardization of multiple records may reduce errors introduced by manual copying from form to form and reduce the work burden on frontline actors.

Conclusion

Frontline health workers are broadly supportive of the move towards digitization despite implementation challenge. Beneficiary willingness to share their information with health workers – including their mobile phone numbers – highlights their trust in health workers and the value they place on government financial incentives. This positive engagement bodes well for derivative uses of HIS, such as for mHealth programs and surveys. However the underlying causes of inaccuracies and severe delays must be addressed before the true benefit of these uses will be realised. In addition to data system and supervisory supports, frontline workers and beneficiaries themselves must understand and experience the benefits of accurate, complete and timely digital health information systems.

For peer review only

Acknowledgements

The authors are grateful to the health system actors who generously provided their time and insights to make this research possible. This work was made possible by the Bill and Melinda Gates Foundation. We thank Diva Dhar, Suhel Bidani, Rahul Mullick, Dr. Suneeta Krishnan, Dr. Neeta Goel and Dr. Priya Nanda for giving us this opportunity. We additionally thank Vinit Pattnaik at OPM and Erica Crawford at Johns Hopkins for their support to the financial management of our work.

Competing interests

All authors have completed the Unified Competing Interest form (available on request from the corresponding author) and declare that the research reported was funded by the Bill and Melinda Gates Foundation. The authors do not have any relationships or engagement in activities that could appear to have influenced the submitted work.

Data sharing

Data for this study consist of qualitative interview and focus group discussion transcripts. Uploading all transcripts for open availability would compromise our ability to fully mask participant details. However, we are happy to share anonymized portions of these transcripts upon reasonable request.

Contributor statement

KS, SC , OU, DM and AEL conceptualized and designed the study. MS, DG, BM and NC conducted the data collection and preliminary data analysis through daily analytic debriefs. KS and OU led and managed the data collection and analysis, including the coding and thematic synthesis. KS drafted the manuscript and revised it based on critical and substantive input from SC, OU, AEL, MS, BM, DM and NC. All authors agree to be accountable for all aspects of the work related to accuracy and integrity.

Funding statement

This work was supported by the Bill and Melinda Gates Foundation grant number OPP1179252.

Works Cited

1. WHO. Framework and Standards for Country Health Information Systems, 2nd Edition. Geneva, Switzerland; 2012.
2. WHO. Health Metrics Network Framework and Standards for Country Health

For peer review only

Information Systems. 2008.
3. Al-Shorbaji N, Geissbuhler A. Establishing an evidence base for e-health: the proof is in the pudding. Bull World Health Organ. 2012;90:322.
4. WHO. Resolution WHA58.28. eHealth. [Internet]. WHA58/2005/REC/1 Fifty-eighth World Health Assembly, Geneva, 16–25 May 2005. Annex. Resolutions and decisions. Geneva; 2005. Available from: apps.who.int/gb/or/e/e_wha58r1.html
5. Lewis T, Synowiec C, Lagomarsino G, Schweitzer J. E-health in low- and middle-income countries: findings from the Center for Health Market Innovations. Bull World Heal Organ. 2012;90:332–40.
6. Neupane S, Odendaal W, Friedman I, Jassat W, Schneider H, Doherty T. Comparing a paper based monitoring and evaluation system to a mHealth system to support the national community health worker programme, South Africa: an evaluation. BMC Med Inform Decis Mak [Internet]. 2014 Dec 9;14(1):69. Available from: https://bmcmedinformdecismak.biomedcentral.com/articles/10.1186/1472-6947-14-69
7. Tamrat T, Kachnowski S. Special Delivery: An Analysis of mHealth in Maternal and Newborn Health Programs and Their Outcomes Around the World. Matern Child Health J [Internet]. 2012 Jul 19;16(5):1092–101. Available from: http://link.springer.com/10.1007/s10995-011-0836-3
8. Schulte F, Fry E. Death by 1,000 clicks: where electronic health records went wrong. Fortune and Kaiser Health News [Internet]. 2019; Available from: https://khn.org/news/death-by-a-thousand-clicks/
9. Källander K, Tibenderana JK, Akpogheneta OJ, Strachan DL, Hill Z, Asbroek A, et al. Mobile health (mhealth) approaches and lessons for increased performance and retention of community health workers in lowand middle-income countries: A review. J Med Internet Res. 2013;15(1).
10. Gera R, Muthusamy N, Bahulekar A, Sharma A, Singh P, Sekhar A. An in-depth assessment of India’s Mother and Child Tracking System (MCTS) in Rajasthan and Uttar Pradesh. BMC Health Serv Res [Internet]. 2015;1–14. Available from: http://dx.doi.org/10.1186/s12913-015-0920-2
11. Balakrishnan R, Gopichandran V, Chaturvedi S, Chatterjee R, Mahapatra T, Chaudhuri I. Continuum of Care Services for Maternal and Child Health using mobile technology – a health system strengthening strategy in low and middle income countries. BMC Med Inform Decis Mak [Internet]. 2016;16(84):1–8. Available from: http://dx.doi.org/10.1186/s12911-016-0326-z
12. Bashingwa JJ, Mohan D, Chamberlain S, Mendiratta J, Arora S, Rahul S, et al. Assessing exposure to Kilkari, the largest maternal mobile messaging programming of its kind in the world: a big data analysis of findings across 13 states in India. BMJ Glob Heal. 2021;
13. SRS. Special Bulletin on Maternal Mortality In India 2016-18. New Delhi; 2020.
14. MoHFW India. National Family Health Survey 4 (2015-2016): State Fact Sheet Rajasthan [Internet]. Mumbai; 2017. Available from: http://rchiips.org/nfhs/pdf/NFHS4/RJ_FactSheet.pdf
15. MoHFW India. National Family Health Survey - 4 (2015-16); State Fact Sheet Madhya Pradesh. 2017.
16. GoI. RCH Application and ANMOL: Data Elements, Validation, Logic and Tool Tip Document, V 2.0. 2016.

For peer review only

17. Attride-Stirling J. Thematic Networks: an analytic tool for qualitative research. Qual Res. 2001;1(3):385–405.
18. Mukherjee A. Who's data is this anyway? Understanding relationship between power, empowerment and technology: Case from Mother and Child Tracking System (MCTS) in India. Proceedings of the 9 th Health Informatics in Africa Conference HELINA'14 PART 2. Hamburg, Germany; 2014.
19. NHSRC and Taurus Glocal Consult. Study of Public Health IT Systems in India. New Delhi, India; 2011.
20. Gera R, Muthusamy N, Bahulekar A, Sharma A, Singh P, Sekhar A. An in-depth assessment of India's Mother and Child Tracking System (MCTS) in Rajasthan and Uttar Pradesh. BMC Health Serv Res [Internet]. 2015;15:315. Available from: http://dx.doi.org/10.1186/s12913-015-0920-2
21. Mphatswe W, Mate K, Bennett B, Ngidi H, Reddy J, Barker P, et al. Improving public health information: a data quality intervention in KwaZulu-Natal, South Africa. Bull World Health Organ [Internet]. 2012 Mar 1;90(3):176–82. Available from: http://www.who.int/bulletin/volumes/90/3/11-092759.pdf
22. The Earth Institute. Pregnancy Child Tracking and Health Services Management System (PCTS) Gap Analysis -- Rajasthan. Mumbai; 2014.

For peer review only

For peer review only - http://bmjopen.bmj.com/site/about/guidelines.xhtml

For peer review only

For peer review only - http://bmjopen.bmj.com/site/about/guidelines.xhtml

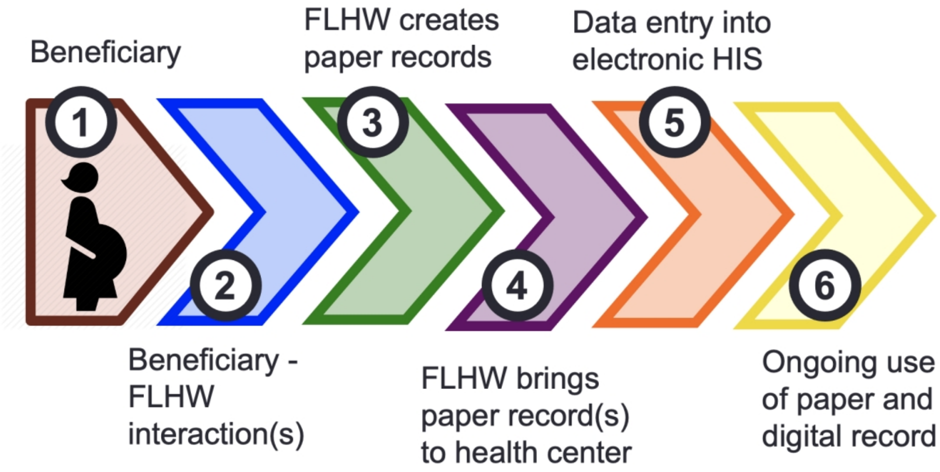
Beneficiary
FLHW creates paper records
Data entry into electronic HIS
1
2
3
4
5
6
Beneficiary - FLHW interaction(s)
FLHW brings paper record(s) to health center
Ongoing use of paper and digital record

NA

COREQ (COnsolidated criteria for REporting Qualitative research) Checklist

A checklist of items that should be included in reports of qualitative research. You must report the page number in your manuscript where you consider each of the items listed in this checklist. If you have not included this information, either revise your manuscript accordingly before submitting or note N/A.

Topic	Item No.	Guide Questions/Description	Reported on Page No.
**Domain 1: Research team and reflexivity**			
Personal characteristics			
Interviewer/facilitator	1	Which author/s conducted the interview or focus group?	
Credentials	2	What were the researcher's credentials? E.g. PhD, MD	
Occupation	3	What was their occupation at the time of the study?	
Gender	4	Was the researcher male or female?	
Experience and training	5	What experience or training did the researcher have?	
Relationship with participants			
Relationship established	6	Was a relationship established prior to study commencement?	
Participant knowledge of the interviewer	7	What did the participants know about the researcher? e.g. personal goals, reasons for doing the research	
Interviewer characteristics	8	What characteristics were reported about the inter viewer/facilitator? e.g. Bias, assumptions, reasons and interests in the research topic	
**Domain 2: Study design**			
Theoretical framework			
Methodological orientation and Theory	9	What methodological orientation was stated to underpin the study? e.g. grounded theory, discourse analysis, ethnography, phenomenology, content analysis	
Participant selection			
Sampling	10	How were participants selected? e.g. purposive, convenience, consecutive, snowball	
Method of approach	11	How were participants approached? e.g. face-to-face, telephone, mail, email	
Sample size	12	How many participants were in the study?	
Non-participation	13	How many people refused to participate or dropped out? Reasons?	
Setting			
Setting of data collection	14	Where was the data collected? e.g. home, clinic, workplace	
Presence of non-participants	15	Was anyone else present besides the participants and researchers?	
Description of sample	16	What are the important characteristics of the sample? e.g. demographic data, date	
Data collection			
Interview guide	17	Were questions, prompts, guides provided by the authors? Was it pilot tested?	
Repeat interviews	18	Were repeat inter views carried out? If yes, how many?	
Audio/visual recording	19	Did the research use audio or visual recording to collect the data?	
Field notes	20	Were field notes made during and/or after the inter view or focus group?	
Duration	21	What was the duration of the inter views or focus group?	
Data saturation	22	Was data saturation discussed?	
Transcripts returned	23	Were transcripts returned to participants for comment and/or	

For peer review only

Topic	Item No.	Guide Questions/Description	Reported on Page No.
		correction?	
**Domain 3: analysis and findings**			
Data analysis			
Number of data coders	24	How many data coders coded the data?	
Description of the coding tree	25	Did authors provide a description of the coding tree?	
Derivation of themes	26	Were themes identified in advance or derived from the data?	
Software	27	What software, if applicable, was used to manage the data?	
Participant checking	28	Did participants provide feedback on the findings?	
Reporting			
Quotations presented	29	Were participant quotations presented to illustrate the themes/findings? Was each quotation identified? e.g. participant number	
Data and findings consistent	30	Was there consistency between the data presented and the findings?	
Clarity of major themes	31	Were major themes clearly presented in the findings?	
Clarity of minor themes	32	Is there a description of diverse cases or discussion of minor themes?	

Developed from: Tong A, Sainsbury P, Craig J. Consolidated criteria for reporting qualitative research (COREQ): a 32-item checklist for interviews and focus groups. *International Journal for Quality in Health Care*. 2007. Volume 19, Number 6: pp. 349 – 357

Once you have completed this checklist, please save a copy and upload it as part of your submission. DO NOT include this checklist as part of the main manuscript document. It must be uploaded as a separate file.

For peer review only

BMJ Open

At the frontlines of digitization: The challenges and opportunities in maintaining accurate, complete, and timely digital health records in India’s government health system

Journal:	*BMJ Open*
Manuscript ID	bmjopen-2021-051193.R1
Article Type:	Original research
Date Submitted by the Author:	03-Dec-2021
Complete List of Authors:	Scott, Kerry; Johns Hopkins University Bloomberg School of Public Health<br>Ummer, Osama; Oxford Policy Management; BBC Media Action, Research and Learning<br>Chamberlain, Sara; BBC Media Action, BBC Media Action, India; BBC Media Action, Asia<br>Sharma, Manjula; Independent researcher<br>Gharai, Dipanwita; Oxford Policy Management<br>Mishra, Bibha; Oxford Policy Management<br>Choudhury, Namrata; Jawaharlal Nehru University<br>Mohan, Diwakar; Johns Hopkins University Bloomberg School of Public Health<br>LeFevre, Amnesty; University of Cape Town
**Primary Subject Heading**:	Health services research
Secondary Subject Heading:	Health informatics, Global health, Qualitative research
Keywords:	Public health < INFECTIOUS DISEASES, Health informatics < BIOTECHNOLOGY & BIOINFORMATICS, Information management < BIOTECHNOLOGY & BIOINFORMATICS, Information technology < BIOTECHNOLOGY & BIOINFORMATICS, HEALTH SERVICES ADMINISTRATION & MANAGEMENT

SCHOLARONE™
Manuscripts

I, the Submitting Author has the right to grant and does grant on behalf of all authors of the Work (as defined in the below author licence), an exclusive licence and/or a non-exclusive licence for contributions from authors who are: i) UK Crown employees; ii) where BMJ has agreed a CC-BY licence shall apply, and/or iii) in accordance with the terms applicable for US Federal Government officers or employees acting as part of their official duties; on a worldwide, perpetual, irrevocable, royalty-free basis to BMJ Publishing Group Ltd ("BMJ") its licensees and where the relevant Journal is co-owned by BMJ to the co-owners of the Journal, to publish the Work in this journal and any other BMJ products and to exploit all rights, as set out in our licence.

The Submitting Author accepts and understands that any supply made under these terms is made by BMJ to the Submitting Author unless you are acting as an employee on behalf of your employer or a postgraduate student of an affiliated institution which is paying any applicable article publishing charge ("APC") for Open Access articles. Where the Submitting Author wishes to make the Work available on an Open Access basis (and intends to pay the relevant APC), the terms of reuse of such Open Access shall be governed by a Creative Commons licence – details of these licences and which Creative Commons licence will apply to this Work are set out in our licence referred to above.

Other than as permitted in any relevant BMJ Author's Self Archiving Policies, I confirm this Work has not been accepted for publication elsewhere, is not being considered for publication elsewhere and does not duplicate material already published. I confirm all authors consent to publication of this Work and authorise the granting of this licence.

For peer review only

At the frontlines of digitization: The challenges and opportunities in maintaining accurate, complete, and timely digital health records in India's government health system

Authors: Kerry Scott[1], Osama Ummer[2], Sara Chamberlain[3], Manjula Sharma[4], Dipanwita Gharai[2], Bibha Mishra[2], Namrata Choudhury[5], Diwakar Mohan[1] and Amnesty LeFevre[1,6]

[1] Department of International Health, Johns Hopkins Bloomberg School of Public Health, 615 N. Wolfe St, Baltimore, Maryland, USA
[2] Oxford Policy Management-Delhi, 4/6 First Floor, Siri Fort Institutional Area, New Delhi, Delhi 110049, India
[3] BBC Media Action, India, Old Fort Saket District Mall, Saket District Centre, Sector 6, Pushp Vihar, New Delhi, Delhi 110017, India
[4] Independent researcher, New Delhi, India
[5] Centre for the Study of Law and Governance, Jawaharlal Nehru University
[6] School of Public Health and Family Medicine, University of Cape Town, Cape Town, South Africa

Corresponding author: Kerry Scott, 615 N. Wolfe St, Baltimore, MD, 21205, kerry.e.scott@gmail.com, 647-675-7956

Abstract

Objectives: To understand factors underpinning the accuracy and timeliness of mobile phone numbers and other health information captured in India's government registry for pregnant and postpartum women. Accurate and timely registration of mobile phone numbers is necessary for beneficiaries to receive mHealth services.

Setting: Madhya Pradesh and Rajasthan states in India at the community, clinical, and administrative levels of the health system.

Participants: Interviews (n=59) with frontline health workers (FLHWs), data entry operators, and higher level officials. Focus group discussions (n=12) with pregnant women to discuss experiences with sharing data in the health system. Observations (n=9) of interactions between stakeholders for data collection and the process of data digitization.

Primary and secondary outcome measures: Thematic analysis identified how key actors experienced the data collection and digitization process, reasons for late or inaccurate data, and mechanisms that can bolster timeliness and accuracy.

For peer review only

Results: Pregnant women were comfortable sharing mobile numbers with health workers but many were unaware that their data moved beyond their FLHW. FLHWs valued knowing up-to-date beneficiary mobile numbers but felt little incentive to ensure accuracy in the digital records system. Delays in registering pregnant women in the online portal were attributed to slow movement of paper records into the digital system and difficulties in gathering required documents from beneficiaries. Data, including women's phone numbers, were hand written and copied multiple times by beneficiaries and health workers with variable literacy. Supervision tended to focus on completeness rather than accuracy. Health system actors noted challenges with the digital system but valued the broader project of digitization.

Conclusions: Increased focus on training, supportive supervision, and user-friendly data processes that prioritize accuracy and timeliness should be considered. These inputs can build upon existing positive patient-provider relationships and health system actors' enthusiasm for digitization.

Strengths and limitations of this study

- This study was strengthened by the use of multiple research methods (interviews, focus groups and observation) and engagement of multiple frontline stakeholders: the women whose data is entered into the system, the frontline health workers who care for them and create the initial (paper) records, the data entry operators who digitize these records, and the managers, administrators and leaders who supervise these processes.

- Frontline health workers and data entry operators were aware that their work was being observed and thus may have minimized or hidden unauthorized behavior, such as shortcuts to speed up data entry or unsanctioned task-shifting.

- Data collection occurred just prior to the launch of a new digitization strategy in some (but not all) regions, wherein frontline health workers would be given tablets and asked to directly digitize patient health data; thus some of the challenges identified in this study may have self-resolved due to this new (shorter) dataflow pathway, and new challenges to accuracy, timeliness and completeness may have arisen.

Introduction

Health information systems (HIS) capture data on patients and their contacts with routine health services. Data collected about patients includes clinical content, such as weight and blood pressure, and non-clinical content, such as their address, patient identification code,

For peer review only - http://bmjopen.bmj.com/site/about/guidelines.xhtml

and phone number. HIS support responsive policymaking, resource allocation, routine service delivery, and health systems accountability.[1] The achievement of these functions is largely dependent on the accuracy, completeness and timeliness of the data in the health information system.[2] Information communication technologies (ICTs) are increasingly being used to improve the core functions of HIS through digitization, wherein patient data is entered into a computer system rather than maintained in handwritten records.[3–5] ICTs for health information systems have great potential to strengthen health systems by streamlining data collection and entry, accelerating the transmission and analysis of data, instituting validity checks, and increasing frontline worker access to a range of clinical and administrative support services. [6,7 8]

The digitization of health information systems has occurred concurrently with increasing mobile phone penetration at a population level and, in turn, an increase in mobile health (mHealth) initiatives. mHealth programs can transmit health-related information, send reminders, or solicit patient feedback on services.[9] To attain high population level coverage at scale, mHealth programs often rely on beneficiary mobile numbers and other clinical and non-clinical data that have been collected through government health information systems. The accuracy, completeness, and timeliness of records in government systems can directly affect the capacity of mHealth programs to reach target populations at the right timepoints.

The Government of India's Kilkari program is one example of an mHealth service that relies on the quality and timeliness of mobile phone numbers and other data collected in the government's health registries in order to reach beneficiaries. Developed and scaled by BBC Media Action in collaboration with the Indian Ministry of Health and Family Welfare, Kilkari is the world's largest maternal mobile messaging initiative, having reached 10 million subscribers in 13 states by December 2018.[10] It delivers maternal, child, and reproductive health information content through up to 72 once-weekly outgoing pre-recorded calls. Beneficiaries (pregnant or postpartum women and their husbands)[11] are subscribed to Kilkari based on the mobile number captured in governmental HIS registries called, depending on the state, the Maternal and Child Health Tracking System (MCTS) or Reproductive and Child Health (RCH) system.[10] The gestational age estimate or child's date of birth is used to determine the appropriate Kilkari messages to send based on the stage in pregnancy or child's age.

Missing and incorrect data in MCTS/RCH reduces the number of beneficiaries who can be exposed to Kilkari. In 2018, one quarter of the unique mobile numbers registered for Kilkari were never reached[12] due to a number of factors including inaccurate or out-of-date mobile numbers and the phone being switched off or out of network. A 2015 study[13] found that one-third of women's profiles in the government registry in Rajasthan and two-thirds in Uttar Pradesh were incomplete. Data delays and late care-seeking impede overall exposure to Kilkari. Kilkari exposure can begin in second trimester of pregnancy, however only 31% of subscribers answered their first call during this period.[12] Over half (58%) answered their first call after they give birth, which means they missed at least one-third of Kilkari's content.[12] Research from Bihar documented an average delay of 72 days between service delivery and being registered in MCTS.[14]

For peer review only - http://bmjopen.bmj.com/site/about/guidelines.xhtml

In order to understand why Kilkari was not reaching every pregnant woman as soon as possible (e.g., in the second trimester) we conducted a qualitative examination of frontline barriers to the accurate, complete, and timely capture of mobile phone numbers and other data in MCTS/RCH. We explored beneficiary perceptions of providing mobile phone numbers to health care providers, and frontline health worker (FLHW) experiences with the digital health record system broadly, and capturing mobile numbers specifically. Study findings will inform health systems in India and beyond as they move towards ICT-enabled strategies to bolster the quality of health information systems.

Methods

Study setting

This qualitative study took place in Madhya Pradesh and Rajasthan, two large Hindi speaking states in central and western India, respectively. These states have high burdens of maternal and child mortality, significant gender gaps around technology access and literacy, and sub-optimal maternal healthcare services (table 1).

Table 1. Social and health indicators, Rajasthan and Madhya Pradesh

Indicator	Rajasthan	Madhya Pradesh
Population	77 million	82 million
Maternal mortality ratio (deaths per 100,000)[15]	164	173
Under five mortality (deaths per 1000 live births)[16,17]	51	65
Literacy[16,17]		
• Female	57%	59%
• Male	85%	82%
Mobile phone access[16,17]		
• Household ownership	94%	84%
• Female access	41%	29%
Maternal health care[16,17]		
• Pregnant women attended antenatal care in first trimester	63%	53%
• Received recommended four antenatal care visits	39%	36%
• Gave birth in a health facility	84%	81%
• Received postnatal health check within 2 days of birth	64%	55%
• Registered pregnancies for which the mother received Mother and Child Protection (MCP) card	92%	92%

Both Madhya Pradesh and Rajasthan moved to digital health records in the late 2000s, however there are important differences between the states, in terms of the digital programs and processes implemented (Table 2). In 2016, Madhya Pradesh transitioned from MCTS to RCH. RCH added urban coverage, an initial registration of all “eligible couples” (married couples of reproductive age) who would then be linked to pregnancy tracking when a pregnancy occurred, and the creation of village profiles.[18] Furthermore, RCH expanded the data elements collected from 111 (in MCTS) to 247 to include abortion

tracking, beneficiary bank account and identification details (including the Aadhaar national identification number), additional details about the pregnant woman's antenatal care, infant feeding practice, and the child's immunization records for his or her first five years.[18] Mobile phone numbers were collected in MCTS and continued to be in RCH. In contrast to Madhya Pradesh, the Rajasthan state government did not adopt RCH but instead retained its state-level version of MCTS, called the Pregnancy, Child Tracking, and Health Services Management System (PCTS), which syncs with MCTS. Annexure 1 contains an explanation of key acronyms and terms.

Table 2. Comparing Rajasthan and Madhya Pradesh's digital HIS

Parameter	Rajasthan	Madhya Pradesh
HIS system currently in use	Pregnancy Child Tracking and Health Services Management System (PCTS), a state-specific system that syncs with MCTS	Reproductive and Child Health (RCH), an expanded and upgraded version of MCTS
Timeline	Adopted PCTS in 2008 (first state in India to launch an electronic health records system) and has not changed to RCH	Adopted MCTS in 2009 and changed to RCH in 2016
Who collects HIS data at the frontline?	Three frontline workers: community health worker called the Accredited Social Health Activist (ASHA Sahyogini in Rajasthan), community nutrition and preschool worker called anganwadi & auxiliary nurse midwife (ANM)	Two frontline workers: ASHA & ANM. The anganwadi worker is not involved.
Feedback mechanisms built into the digital record system	Creates a workplan for the ANM telling her who is due for antenatal care, delivery, postnatal care as well as listing drop outs; sends SMS alerts to beneficiaries (program called Swasthya Sandesh Sewa)	Creates a workplan for the ANM telling her who is due for antenatal care, delivery, and postnatal care, as well as listing drop outs; sends SMS alerts to beneficiaries and also to health functionaries at different levels
Paper forms involved in data collection for pregnancies	***1) Pregnant women's antenatal care registry form***: Paper where up to five new pregnancies being managed by the frontline worker can be listed with details of first antnatal care visit. This form is filled in by the ASHA/ANM and passed to the data entry operator for entry into PCTS.<br>***2) Mamta card***: Card filled by ANM/ASHA; stays with the pregnant woman<br>***3) Reproductive and Child Health (RCH) register***: Book kept with the ANM or at the health center; all pregnancies under the ANM are in this book. It is passed to the data entry operator for data entry.<br>***4) Personal ANM diary***: Unofficial paper book that ANMs fill (because RCH register is sometimes too big to	***1) RCH form***: One form per woman, filled by ASHA/ANM and given to the data entry operator. Meant to travel back and forth between ANM and data entry operator as the woman proceeds through her antenatal care visits and delivery.<br>***2) Mother Child Protection (MCP) card***: Card filled by ANM/ASHA; stays with the pregnant woman<br>***3) RCH register:*** Book kept with the ANM or at the health center; all pregnancies under the ANM are in this book. It is passed to the data entry operator for data entry.<br>***4) Personal ANM diary***: Unofficial paper book that ANMs fill (because RCH register is sometimes too big to bring to the village or is with the data entry operator)<br>***5) Personal ASHA diary***: Official paper book that the ASHA fills

For peer review only - http://bmjopen.bmj.com/site/about/guidelines.xhtml

Parameter	Rajasthan	Madhya Pradesh
	bring to the village or is with the data entry operator)<br>***5) Personal ASHA diary***: Official paper book that the ASHA fills	

The states thus enable us to examine data systems in a more typical case (Madhya Pradesh), which, like most large Indian states, recently moved from MCTS to RCH, and an outlier case (Rajasthan) which has retained a tailor-made electronic record system since the beginning of digitization.

Data collection

Five experienced qualitative researchers with master's level social science degrees (authors OU (male) MS, DG, BM and NC (female)) conducted in depth interviews (n=59), focus groups (n=12) and observation of data collection and entry (n=9) in Rajasthan and Madhya Pradesh in September 2018 (table 3). All researchers were trained over a one-week period, which included pilot testing the FLHW interview guide. In each state, we selected one district and two blocks within that district with high levels of female phone ownership to explore barriers to capturing women's mobile phone numbers in MCTS/RCH. We sampled government health system actors who were involved in MCTS/RCH at the state, district, block and frontline levels, including medical officers, data entry officers and frontline providers, as well as with women who recently interacted with government healthcare providers in situations where they were asked to register their mobile numbers in MCTS/RCH (table 3).

Table 3. Respondent sample

Respondent type	Respondent profile	MP	RJN	N
In depth interviews (IDIs)		Number of IDIs		
State level stakeholders	Senior government employees of the department of health and family welfare who are in charge of data	3	1	4
District stakeholders	Community mobilizers, District Programme Manager, District Community Mobilizer, District Monitoring and Evaluation Officer, District Nodal Officer	3	4	7
Medical officers (MOs) at primary health centers	Doctors (allopathic, homeopathic or ayurvedic), 5.5 years training	2	4	6
Data entry operators (DEOs)	Information technologists with undergraduate level education	4	6	10
Auxiliary nurse midwives (ANMs)	Female maternal and child health worker with 1.5 years training (6 months midwifery focused)	10	9	19
Accredited Social Health Activist (ASHA) community health worker	Female volunteer community health worker, received incentive based remuneration, initial 24 days training and periodic week-long additional training	3	5	8
Other stakeholders at the PHC and block level	Block Programme Manager, Multipurpose Health Supervisor, Primary Health Centre Supervisor	2	3	5
Total IDIs		27	32	59
Focus Group Discussions (FGDs)		Number of FGDs		
Beneficiaries	Pregnant and post-partum women with mobile phones who recently interacted with health system actors for MCTS/RCH registration	6	6	12

For peer review only - http://bmjopen.bmj.com/site/about/guidelines.xhtml

Observation		Number of observations		
Beneficiary – FLHW interaction	Observation of beneficiary – FLHW interaction (antenatal or post-partum) where MCTS/RCH data recorded	1	1	2
Data entry into electronic system	Observation of data entry into electronic system	3	4	7
Total observations		4	5	9

The respondents were approached through their government health facility. A research team member contacted potential respondents by phone or face-to-face and explained the study, and that the team was from a Delhi-based company and had governmental approval, then invited them for a face to face meeting to learn more and, if they agreed, to participate. The study information and informed consent was read to each potential participant and then summarized in conversational language to ensure comprehension. While all the health facility staff approached for the study agreed to participate while three women invited to attend focus groups declined, siting responsibilities at home. The interviews took about an hour and the focus group discussions took about an hour and a half; all were conducted in health faculties and audio recorded and detailed notes were taken. When curious onlookers came over during focus groups and interviews, another researcher politely asked them to move on. If any supervisors, patients or family members stopped by to speak to the respondents during the interviews or focus groups the research paused until privacy was restored.

Interviews and FGDs were conducted using semi structured guides that explored a range of domains around sharing, documenting, inputting, and using data, with a focus on mobile numbers. We explored potential drivers of inaccuracies and delays by asking about late pregnancy identification, FLHW work environment, and the relationship between beneficiaries and FLHWs. In the interviews with health system actors we also explored each step of a detailed description of data flow (Fig 1) from when a pregnant woman first interacts with a FLHW until her health information is entered into the online portal, and beyond to understand perceptions on the use of this data.

For peer review only

Ethics approval

This study was approved by the institutional ethics review boards at Sigma, Delhi, India (10041/IRB/D/17-18) and JHU, Baltimore, USA (00008360). All respondents provided informed oral consent.

Data analysis

Daily debriefs enabled the team to share emergent findings, refine the focus of their probing for the next day's data collection, and identify areas of saturation. The audio files were transcribed and translated into English. Data were coded in Dedoose by OU and KS, using principles of thematic network analysis.[19] A coding framework was developed that consisted of emergent codes on specific reasons for inaccurate and delayed data, that were then grouped according to an overarching data flow framework (Fig 1). For instance, we created a code cluster for late antenatal care registration, which included codes to be applied to text describing when and how pregnancies come to the FLHW's attention, when

pregnancies were entered into the online portal, reasons FLHWs become aware of pregnancies late (after the first trimester), and the implications of a woman's choice of the public or private sector for antenatal care on timeliness of registration. After coding, we read the text excerpts that had been tagged for specific codes to identify how key actors experience of the data collection and digitization process, reasons for late or inaccurate data, and mechanisms that can bolster timeliness and accuracy.

Conceptual framework

While we initially set out to understand inaccuracies and delays in entering mobile phone numbers into the pregnancy registry, it became clear that this one piece of data could not be separated from the broader data collection process at the frontlines of government health service provision. We thus examined mobile phone number data collection and digitization within the context of an overarching health system data flow framework (Fig 1). This framework identifies six components that enable the creation and movement of health data, including mobile phone numbers, from beneficiaries through FLHWs to electronic data entry and onward.

Figure 1. Data flow framework for electronic health record systems

The first component, "beneficiary," describes women's access to the data required for documentation (e.g., whether they have a mobile phone number to provide), as well as their attitudes towards sharing this data with government health system functionaries (e.g., their willingness to provide their mobile numbers when asked). The second component considers the beneficiary-FLHW interactions that initiate the government health system's awareness of a health event. In this section we consider how and when FLHWs become aware of a new pregnancy, the value that FLHWs place on collecting beneficiary data, and the strategies used to collect data. The third component involves the creation of initial paper records. In this section we consider the various official and unofficial forms and registers where data is recorded, the health worker's literacy and numeracy if data is copied from paper form to paper form, and potential delays between a health worker interacting with a beneficiary and a paper record being created. The fourth component considers the process through which a paper health record reaches the site of digitization. We consider how often the FLHW visits the digitization site and whether the FLWH waits to bring paper records for digitization until all mandatory fields have been filled out. While steps three and four may be dropped as FLHWs directly create digital records themselves, in many health systems in lower resource settings, paper records are still created during outreach service provision. The fifth component is the time when data is digitized and considers the data entry personnel's work environment, training, the electronic portal interface, and staffing considerations. The final component examines the ongoing use of data through paper forms and online systems. Here we consider how data can be corrected or updated, data monitoring and supervision, and the use of data by FLHWs and higher level health system actors.

For peer review only - http://bmjopen.bmj.com/site/about/guidelines.xhtml

Patient and Public Involvement

The research was shaped by health system actor priorities, experiences and preferences through iterative probing and flexibility within our research domains. Results were disseminated to Government of India stakeholders but not to research participants due to the policy-level implications of our findings.

Findings

Our findings are organized conceptually according to the six components in the general data flow framework. For each step, we provide a description of how this aspect of the data system is experienced by the actors involved, and identify barriers and facilitators to timeliness and accuracy.

Level 1. Beneficiary

Women faced barriers to accessing phones and services that generate the data required by MCTS/RCH

Women who did not have their own personal mobile phones could not provide their number. Many women lived in households with phones so could provide their husband's phone number or the number for a shared phone. Women living in households without a phone could provide a neighbor's phone number or a FLHW's number.

> R: Some women have their husband's mobile number. In some cases both husband and wife don't have mobile phones. Some are from poor families where both of them don't have mobile phones.
> I : So, what do you do in such situation?
> R: Either we take a relative's phone number or a neighbor's. […] If any laborer is there who does labor work how will he have mobile phone? Even if it's there then her husband must have taken it while going for labor work.
> (MP_IDI_FLHW_06, ANM)

Additional data required from beneficiaries included their bank account numbers and branch names (for both the beneficiary and her husband), national identification (Aadhaar), ration card number, and state identification numbers (Bhamashah state health insurance in Rajasthan and Samagra in Madhya Pradesh). FLHWs explained that women often did not have all of these items or did not know this information. ASHAs were tasked with following up with women to encourage them – or at times personally help them – open bank accounts, register for identification numbers, and provide photocopies of this information.

> Like the [bank] account number, Aadhaar number we get them very rarely. We have to with the ASHA then go again to get information. We fill the form and submit it and it's fed in. If half the information remains incomplete then we can't do the RCH. (MP_IDI_FLHW_01, ANM)

For peer review only - http://bmjopen.bmj.com/site/about/guidelines.xhtml

Women were generally comfortable sharing data with frontline health workers

Women and FLHWs overwhelmingly reported that beneficiaries with access to a mobile phone shared their mobile number with their ASHA or ANM without hesitation and without questioning. They explained that women trusted FLHWs and felt that the number would only be used to help them, such as to convey important information about health services or financial benefits through government programs.

> They are observing me from 20 years so they are aware that madam will not misuse the number so they never deny giving number (RJN_IDI_FLHW_25, ANM)
>
> [Frontline health workers take our mobile number] so that they can give us a phone call next time to call us whenever vaccination is to be given. That's why. (RJN_FGD_BENEF_07)
>
> They are aware that the mobile number is essential as when the money will come in the bank account then they will get the message on mobile. We take the mobile number for their comfort only as we can call them as required. […] And calls also come on their mobile who tells them about the health. (RJN_IDI_FLHW_27, ASHA)

There were exceptions to this predominantly positive view of sharing mobile numbers with health workers. FLHWs and beneficiaries reported that a very small minority of women resisted sharing their numbers with them because of concern about receiving calls from strangers or because they had hidden their mobile phone.

In Madhya Pradesh, most women thought that only the ASHA had their number. When told that their numbers were passed onward from the ASHA to the ANM, women were unconcerned. In Rajasthan, women were generally aware that their mobile numbers were collected for use beyond beneficiary-ASHA communication. They clearly explained that their mobile number was linked to their receipt of financial benefits. While most women in Rajasthan said the only calls they received from government actors were from their ASHA, calling them for checkups, a few received informational calls from the government of India, which were likely Kilkari messages, and one said that the government would use their mobile numbers to call them and check "whether the facilities which government is providing are reaching us or not" (RJN_FGD_BENEF_09).

Level 2. FLHW-beneficiary interaction

Early identification of most pregnancies – with notable exceptions

Digital records of pregnancies can only be created after the government health system functionaries identify a pregnant woman. ASHAs are a pregnant women's first point of contact with the government health system. ANMs rely on ASHAs to identify pregnant women and encourage them to meet the ANM for antenatal care. Beneficiaries and FLHWs reported that ASHAs were notified within the first four months about almost all pregnancies.

For peer review only - http://bmjopen.bmj.com/site/about/guidelines.xhtml

However, FLHWs noted that women living in remote communities, migrants and less educated women, may delay seeking antenatal care due to “carelessness” (RJN_IDI_FLHW_27, ASHA), superstition, and not knowing that they were pregnant. Wealthier women who received antenatal care in private facilities would also be missed by the government health system or registered only after giving birth.

FLHWs place high value on collecting and digitizing beneficiary data

FLHWs in Rajasthan and Madhya Pradesh articulated a clear focus on accurately collecting and digitizing women’s bank account details and identification numbers, since this data enabled women to receive financial support from government programs. While phone numbers were considered important for ASHA-beneficiary communication, the value of digitizing this information (rather than just ensuring it was collected in the ASHA’s paper records) was not always clear. Some health workers in Madhya Pradesh could only articulate a vague sense that entering mobile numbers in RCH was useful. Most FLHWs in Madhya Pradesh reported that mobile numbers were used by the state or central government to call beneficiaries and check whether they are receiving appropriate health services, suggesting that FLHWs saw the digitization of women’s mobile numbers as a means for the government to keep tabs on FLHW performance. In Rajasthan, FLHWs were clear about the value of mobile numbers in PCTS, and explained that the numbers were not only used for checking that women were receiving health services, but also for conveying health information, advertising government programs, and notifying women about financial transfers.

FLHWs describe several strategies for trying to collect accurate data

In Rajasthan and Madhya Pradesh, ASHAs and ANMs explained that many women could not accurately recall their mobile numbers at antenatal care visits. For cases when women did not have mobile phones with them, ASHAs would visit their homes and ask other family members to provide a mobile number or ASHAs and ANMs would ask women to bring a mobile number written on a piece of paper to the next antenatal care checkup.

> Like, they don't know, some of them.. they don't know the mobile number. So, we tell them to ask the people in her house who can write, to note it down on a piece of paper, and bring it to us. Tell them the sister [nurse] needs it, for your information. So they do that. (MP_IDI_FLHW_01, ANM)

ASHAs were confident that they received accurate numbers because they reported frequently calling women using the number provided. In cases when women had mobile phones with them, some ANMs or ASHAs reported giving themselves a missed call from beneficiary mobile phones to identify the number; however, the use of missed calls was not a universal practice, as observed during beneficiary – FLHW interactions.

FLHWs said that pregnant women sometimes asked FLHWs to record their husband’s mobile number rather than their own, even when she had her own phone. Women in one FGD (RJN_FGD_BENEF_10) explained that the husband’s mobile number was often provided so that he would receive the SMS notification of a financial transfer into the beneficiary’s bank account. While some FLHWs complied in taking the husband’s number, others tried to

For peer review only - http://bmjopen.bmj.com/site/about/guidelines.xhtml

convince woman to provide her own mobile number since her husband will frequently be out of the house with the mobile.

FLHWs reported asking women to provide photocopies of bank account information and government identification cards, in order to reduce errors that could be introduced by women conveying this information on scraps of paper. When assessing last menstrual period in order to estimate gestational age, two FLHWs noted that many women struggled to report exact dates. FLHWs estimated last menstrual period based on women's recall of the moon cycle, harvest, or festivals, which would introduce inaccuracy around pregnancy stage.

Level 3. Creation of paper records

Beneficiary data copied by ASHAs and ANMs across multiple paper forms and registers

In Madhya Pradesh and Rajasthan, when an ASHA identified a pregnancy in the village she would write key information about the woman in her official ASHA diary. In cases where ASHAs were considered sufficiently literate by the local ANM, the ASHA was also expected to start a paper RCH form (in Madhya Pradesh) or ANC form (in Rajasthan) by filling in some initial fields. In Madhya Pradesh we found that ASHAs in one block reported being paid Rs. 50 ($0.80) for each form they created, while in a nearby block ASHAs received no payment. In both states, some ASHAs first wrote the pregnant woman's details in an unofficial notebook and then copied this information into the official ASHA diary and the RCH/ANC form. While some ASHAs were clear and confident about their role filling in the RCH/ANC form, others were unsure about which documents they completed.

When the woman comes the village health and nutrition day (an outreach event where antenatal care is provided by ANMs), the ASHA will pass the partially-filled RCH/ANC form to the ANM. The ANM will fill in additional details from the woman's first antenatal care visit, often with the ASHA's help. ANMs explained that they were overwhelmed by documentation requirements and thus had to take assistance from ASHAs, even when ASHAs struggled with the literacy and numeracy requirements of this work.

> We have to fill a big register, we make sehyogini [ASHA] fill it that is the reason it gets wrong. Now they [ASHAs] don't understand. They themselves have filled the card. Now, there was so much crowd that time, that I was writing something.. and if they say, the weight is 53 and she says its 61, so then there's confusion like that. So sometimes they write it wrong. When there's a crowd sometimes, we take help from them only, and it becomes wrong. (MP_IDI_FLHW_07, ANM)

In addition to the RCH/ANC form, ANMs record data about pregnant women in the ANM RCH register (a book) and create a Mother and Child Protection (MCP) card (Madhya Pradesh) or Mamta card (Rajasthan). MCP/Mamta card is given to the pregnant woman to keep. Similar to our observations of ASHAs, ANMs in Madhya Pradesh also sometimes wrote down beneficiary information in unofficial notebooks and then copied this information into

For peer review only - http://bmjopen.bmj.com/site/about/guidelines.xhtml

their official RCH register and the RCH forms once they returned home; they explained that the RCH register is heavy and they wanted to keep it neat and clean. In Rajasthan the official register was smaller and more portable so ANMs tended to carry it with them.

Delays in antenatal care have implications for the creation of health records

There could be several weeks or even months between the ASHA becoming aware of a pregnancy and the ANM meeting the woman for first antenatal care. No paper record could be completed until the woman interacted with an ANM for antenatal care, thus introducing delay in the production of official health records.

FLHWs found data collection under RCH to be too time-intensive

ANMs in Madhya Pradesh said that they found data collection to be highly burdensome. They felt that their work had shifted from healthcare provision to finding documents and filling in forms. FLHWs described facing pressure to complete the digital records from senior actors in the health system hierarchy as well as from beneficiaries who were worried about delays in receiving their financial incentives. They described having to fill up to 70 data fields in various paper forms and registers, and having to track down beneficiary banking and identification details, without adequate time or support.

> We have to do this also and that also. In this what happens is, like this “Prashuti Sahayata Yojana” has come [i.e., yet another scheme has been introduced]. So, we keep on calling them the whole day that “Get this paper” “This is not enough” “This paper is short.” Just now also madam [a supervisor] called. And sir [another supervisor] also called saying that high risk pregnancy too needs to be checked too. (MP_IDI_FLHW_06, ANM)

> They've increased our work a lot, a lot. … We need to visit each beneficiary 10 times… “Give the Aadhar card, the Samagrah ID.” The beneficiary isn't able to submit, isn't able to give it to us, and above that they reach here and start complaining, that, madam isn't filling our form so we aren't getting our money when they themselves aren't giving us the documents all together. We used to go to each house, of the beneficiary, give them the vaccinations, meet each one. And the bai [women] that were there, they had very good behaviour, were respectful, and would make us sit inside, that come, madam, sit inside. Would treat us very well. And, these days I don't know what is happening, so the beneficiaries also snap at us, for a little bit. If they say, madam we need the money, and we tell them to submit the documents, and they'll get the money... Oh oh, but you need this paper, and that paper, and we've given it to the Anganwadi, to you, and to the ASHA, what is this nonsense. (MP_IDI_FLHW_07, ANM)

ANMs who worked with low-literacy ASHAs noted that they faced an additional struggle because they could not rely on the ASHAs to complete any paper forms.

For peer review only - http://bmjopen.bmj.com/site/about/guidelines.xhtml

Level 4. Paper records brought to health centre

Delay between creation of paper records and records reaching the health center with a computer

The ANM becomes aware of a pregnancy during the woman's first antenatal care visit and considers the woman's pregnancy to have been registered at this time. However, the pregnancy is not entered into the online portal until the ANM brings the RCH/ANC forms and RCH registers to the health center for digitization. This occurs only once per month. In addition, if an ANM is waiting for a beneficiary to provide documents required to complete the RCH/ANC form, she will hold on the form even longer, until a future visit to the health center.

> There are some women in the village, even after their delivery, their Samagra ID isn't able to be made..[…] [So] It's not fed [entered into RCH] here because the Samagra ID is incomplete. (MP_IDI_FLHW_01, ANM)

Level 5. Data entry

DEOs spoke of high workloads and some technical challenges but noted recent improvements and supportive peers

DEOs in both states said there was excess workload, which could lead to data entry backlog. They explained that in addition to entering maternal and child health data they were also responsible for entering data from the pharmacy, ASHA records, birth and death registration, HMIS, and health facility forms. In some areas, one DEO was covering the workload of two or three because of vacancies. ANMs suggested that DEOs may make data entry errors, because they were rushing or careless.

In both states the DEOs mentioned that the infrastructure to support them was improving, such as the provision of newer computers, faster internet, and upgraded servers. In Rajasthan the DEOs had battery backup for power outages and a dedicated broadband internet connection; even so, during observation, opening the PCTS portal took up to 10 minutes because of slow internet connection or server capacity. In Madhya Pradesh, many DEOs did not have functioning battery backup, leaving them unable to work when the electricity went off. Some relied on their own portable WIFI device or their mobile phone's hotspot, both of which were often slow.

DEOs in Madhya Pradesh reported having received only one training on RCH, while DEOs in Rajasthan received frequent trainings and felt reasonably well supported by the supervisory structure as well as their peers. The one-time training in Madhya Pradesh occurred years ago, and many months before RCH was introduced so recipients forgot much of it. It consisted of a presentation without any hands-on learning. DEOs reported learning how to use RCH on the job, through trial and error and from other DEOs, who provided ongoing peer support.

For peer review only

Required fields and auto-fill invite errors

The digital portals had numerous required fields, validation checks, and autofill features, including that 10 digits be entered in the mobile number field. In many cases these features supported accurate and complete data entry. However, DEOs recalled instances where these features forced them to introduce errors into the system. For instance, when DEOs encountered forms that did not have a beneficiary mobile number, DEOs reported using the ASHA's number or their own number because the mobile number field is mandatory. In Madhya Pradesh, DEOs reported that RCH initially required all mobile numbers to start with the digit 9. When a new phone company began issuing mobile numbers beginning with 6 or 7, DEOs could not get the system to accept these numbers until the RCH portal was updated.

In RCH, pregnancy records are linked to eligible couple record. The pre-existing eligible couple registry auto-completes fields in the new pregnancy registry, including for the mobile number. Thus the phone number provided by a family at time of marriage becomes the default current phone number at the time of pregnancy.

DEO and ANM absences can lead to data entry delays and may compromise quality

When DEOs took time off, there were not always additional trained personnel to fill in. Other health system actors (such as a PHC health supervisor or ASHA supervisor) would take over data entry, even without training. In cases of longer-term absence, such as when there was no DEO appointed to a PHC at all, a DEO at a neighbouring facility would be assigned to cover both catchment areas. ANMs then had to bring their paper records to a more distant health facility to have them digitized and DEOs had to cover double the caseload.

There are gaps in data collection at the frontlines when ANMs take time off, during transition from an older ANM to a new recruit, or when the ANM position is vacant. During these periods no health records are created or digitized but the district health system actors demand that data continues to be entered. This leaves the DEO in a difficult situation wherein no paper records are being passed on to them but they are expected to create digital records.

Level 6. Ongoing use of paper and online records

Mobile numbers are not corrected or updated in the online system

Beneficiaries explained that they updated their ASHA whenever they changed mobile numbers and ASHAs were confident that they retained up to date mobile numbers for the pregnant women in their villages. However, ASHAs, ANMs and DEOs reported that they do not update or change mobile numbers in the online portals. Some DEOs were unsure if it is even possible to do so. Checking and correcting bank account details and government identification details was common, since this ensured financial transfers proceeded. Ensuring correct and up-to-date mobile numbers was not a priority. Even among women and FLHWs who identified benefits for women who shared their mobile number with the

For peer review only - http://bmjopen.bmj.com/site/about/guidelines.xhtml

government, the value of receiving government information and notifications was overshadowed by the importance of receiving financial incentives.

Supervision focuses on completeness not accuracy

DEOs as well as block, district and state level actors focused on data entry completion and timeliness but not on accuracy. DEOs were encouraged to complete their data entry activities each month and clear backlog. However attention to data accuracy only arose when mistakes came to light through irregular means, such as a financial transfer going into the wrong bank account or a polio outbreak alert being triggered by a DEO incorrectly entering eight polio cases rather than zero.

In Madhya Pradesh, some health facilities had multi-purpose supervisors and ASHA support staff who checked the forms and registers provided by ANMs and ASHAs to the DEO and who oversaw the DEOs work. These supervisors could flag potential inaccuracies for follow up. However in other facilities these positions were vacant. The Medical Officer was expected to sign off on the DEO's work but was so busy with patient care that they often gave an automatic approval or even provided their login and password to the DEO so that the DEO could approve their own work.

In both Madhya Pradesh and Rajasthan, DEO work at the health centers was overseen by block level supervisors. In both states these actors reported being overwhelmed by having to oversee five to seven software programs and more than 50 reporting processes and could only focus on issues of incomplete data.

At the frontlines, ANMs and ASHAs in Madhya Pradesh and RJN had no way of knowing whether the mobile number entered in PCTS or RCH was correct and had never been asked to cross check these digital records with beneficiary numbers.

Health system actors have an overall positive view of the value of digitization

Despite noting hiccups and increased documentation burden, ANMs and ASHAs were generally positive about the move towards digital health records. They framed the use of digital systems as a better way for them to showcase their hard work and to receive recognition (and, for ASHAs, performance based remuneration), rather than a way to benefit from the digital system's ability to generate ANM workplans or other synthesized data. Block, district and state level actors described benefiting enormously from the digital system. The explained that it enabled them to see the progress of programs, monitor service provision by FLHWs, conduct yearly planning to address gaps, and prioritize resources: "In one click the full report can be retrieved" (RJN_IDI_MO_04, doctor).

Discussion

This study explored how beneficiaries, frontline health workers, data entry operators and higher level actors in the government health system experienced HIS digitization to understand barriers to accurate, complete, and timely capture of mobile phone numbers

For peer review only - http://bmjopen.bmj.com/site/about/guidelines.xhtml

and other data (Table 4). We found that frontline workers and their supervisors prioritized the accuracy of administrative data elements that enabled pregnant clients to receive government financial entitlements (e.g., bank account information, identification numbers) over mobile phone numbers. Providers reported that it often took months – and significant effort that detracted from healthcare provision – to gather the documents and information from pregnant clients required to create a digital record; frontline workers relied on their existing paper systems to provide ongoing healthcare and keep track of up-to-date beneficiary mobile numbers.

For peer review only

Table 4. Summary of barriers to the creation of timely, accurate and complete mobile phone records in MCTS/RCH data

	Barriers to timely data	Barriers to accurate and complete data
1. Beneficiary	• Beneficiaries must open bank accounts and attain government identification before an electronic record can be created.	• Duplicate mobile phone entries are created when one number is provided by multiple pregnant women, such as when multiple women in a joint family with one phone become pregnant, or when multiple women provide a neighbour's number or the ASHA's number.<br>• Non-beneficiary numbers are entered when women without personal mobile phones provide their husband's number, a joint family phone number, a neighbour's number, or the ASHA's number.
2. Beneficiary-FLHW	• While ASHAs learn of most pregnancies within the first trimester, pregnancies among migrants, women living in remote communities, very poor women who did not see the value in seeking early antenatal care, and wealthy women who received antenatal care in the private sector were all detected late – sometimes even after the baby was born.	• FLHWs value collecting correct mobile numbers so that ASHAs can keep in touch with beneficiaries. The value of accurately digitizing these numbers is not always clear to FLHWs in Madhya Pradesh. In both Rajasthan and Madhya Pradesh accurate mobile phone number digitization does not have immediate or direct influence on health care or financial transfers.<br>• While ASHAs and ANMs have a number of strategies to seek correct mobile numbers, beneficiaries may incorrectly recall their mobile numbers; checking the beneficiary's number through a missed call is common but not universal and is not an official requirement.<br>• Accurate gestational age estimation (which is linked to the provision of stage-based information in Kilkari) is a challenge since women cannot always report the exact date of their last menstrual period.
3. FLHW creates paper records	• There can be a delay of weeks or even months between an ASHA learning of a new pregnancy and the woman receiving her first antenatal care from the ANM at village health and nutrition days.<br>• It is only after the woman meets the ANM that her (paper) antenatal care / RCH form can be filled in with details of the first antenatal care visit.	• Copying errors can occur when ASHAs and ANMs write women's information in multiple places for different programs, often with slightly different data fields; beneficiaries may also provide their mobile phone numbers on scraps of paper.<br>• ASHAs are often expected to complete many fields in the RCH/ANC form before the ANM fills in additional details and passes it to the DEO; ASHAs with lower literacy struggled with this responsibility.<br>• FLWHs in Madhya Pradesh found data collection for RCH to be highly burdensome and described high pressure from above to fill forms and registries without adequate time or support.<br>• Some ASHAs were confused about the names and purposes of various paper forms and some hand made their own additional registries that they use in the field.
4. FLHW brings paper records to health centre	• Many ANMs bring paper antenatal care / RCH forms and registers to the health center for data entry only once a month.<br>• A woman's paper form cannot be passed to the health center until she	• Respondents did not note any risks to data accuracy or completeness while transporting paper forms and registers from the field to the data digitization facility.

For peer review only

	Barriers to timely data	Barriers to accurate and complete data
	has provided all required fields (such as a bank account number), which may introduce further delays.	
5. Data entry into online portal	• Server errors, internet and electricity issues may delay DEO in creating electronic records.<br>• If DEOs are overburdened with data entry activities, they can fall behind.	• When entering a pregnancy into the online portal, RCH auto-fills the mobile number provided at the time of "eligible couple" registration; this number may be outdated.<br>• DEOs in Madhya Pradesh found their training on RCH to be insufficient; peer support enabled DEOs to navigate challenges. DEOs in Rajasthan were confident in PCTS and received frequent training.<br>• When DEOs are on leave or when DEO positions are vacant result in other health facility employees, who have not been trained, perform data entry.
6. Ongoing use of paper and online records	None identified	• Although the ASHA is generally notified if a pregnant woman's contact number changes over the course of her pregnancy or postpartum, this new number will not be updated in the PCTS/RHC portals.<br>• Supervisor positions remain vacant in some health facilities and higher level supervisors (block and district level) oversee numerous databases and reporting systems.<br>• Supervisors tend to focus on completeness and timeliness rather than accuracy.

For peer review only

Scholars have critiqued India's health system digitization as disproportionately focused on the collection of data rather than its use.[20,21] However the health system actors that we interviewed conveyed a clear understanding of ways in which the digital HIS supports work planning and monitoring. Nonetheless, the overarching logic behind digitization articulated by frontline workers remained as a mechanism to feed information up the health system hierarchy rather than to improve their work planning or to access information about their clients.

While pregnant women were willing to share their or their family's mobile numbers with ASHAs and ANMs, many were unaware that their mobile numbers were passed onward and entered into computers. Although women trusted the health workers and associated the provision of requested data with receiving benefits from the government, strengthening consent processes can protect beneficiaries and safeguard relationships at the frontlines. These processes can inform beneficiaries and frontline providers about the benefits of ensuring accurate mobile numbers are registered in the digital system (including that beneficiaries can be subscribed to mHealth programs such as Kilkari), potentially increasing both parties' motivation to keep the registry up-to-date.

As identified earlier by other researchers,[22] we found poor standardization across multiple data systems (paper based and digital) and identified opportunities for streamlining records and entry options to become more intuitive and user friendly. Echoing findings from other settings,[23] supervision focused on completeness rather than accuracy, highlighted by the fact that there were no mechanisms to check whether mobile numbers were correct. Workers at the frontlines require clear guidelines on how to handle common challenges in accessing data, particularly mobile phone numbers, including how to handle data changes (such as new phone numbers) and unavailable data (such as no phone number). Health workers require training on the value of maintaining an up-to-date mobile number in their data registries, so that they are motivated to ensure these numbers are correct. Furthermore, in light of research findings that "SIM churn" (changing SIM cards, and as a result getting a new mobile number) is as high as 44% per year among rural families,[10] frontline workers need to regularly confirm beneficiary mobile numbers and have access to user-friendly mechanisms to update numbers whenever they change.

Required fields, auto-fill features and validation checks can force DEOs to adjust the data provided to them according to their judgement in order to proceed with data entry. Another study on PCTS in Rajasthan found that the portal would not accept delivery dates that appeared to occur after more than 9 months gestation, a situation created by incorrect gestational age estimation based on faulty last menstrual period estimates.[24] Finally, the consolidation and standardization of multiple records may reduce errors introduced by manual copying from form to form and reduce the work burden on frontline actors.

Three core determinants of accurate, complete and timely administrative data emerged from our research and are likely applicable to health registries for pregnant and postpartum women in other lower resource settings. First, beneficiaries who trust their health workers and the government more broadly are willing to provide accurate data.[25,26] Second, data entry systems that are easy to use, appropriate to the local context, and useful to the

For peer review only - http://bmjopen.bmj.com/site/about/guidelines.xhtml

frontline worker facilitate data accuracy and timeliness.[8,27] And third, supportive supervision and adequate resources are vital to building frontline health worker skills and knowledge across clinical and administrative functions.[28–30]

Conclusion

Frontline health workers are broadly supportive of the move towards digitization despite implementation challenge. Beneficiary willingness to share their information with health workers – including their mobile phone numbers – highlights their trust in health workers and the value they place on government financial incentives. This positive engagement bodes well for derivative uses of HIS, such as for mHealth programs and surveys. However the underlying causes of inaccuracies and severe delays must be addressed before the true benefit of these uses will be realised. In addition to data system and supervisory supports, frontline workers and beneficiaries themselves must understand and experience the benefits of accurate, complete and timely digital health information systems.

Acknowledgements

The authors are grateful to the health system actors who generously provided their time and insights to make this research possible. This work was made possible by the Bill and Melinda Gates Foundation. We thank Diva Dhar, Suhel Bidani, Rahul Mullick, Dr. Suneeta Krishnan, Dr. Neeta Goel and Dr. Priya Nanda for giving us this opportunity. We additionally thank Vinit Pattnaik at OPM and Erica Crawford at Johns Hopkins for their support to the financial management of our work.

Competing interests

All authors have completed the Unified Competing Interest form (available on request from the corresponding author) and declare that the research reported was funded by the Bill and Melinda Gates Foundation. The authors do not have any relationships or engagement in activities that could appear to have influenced the submitted work.

Data sharing

Data for this study consist of qualitative interview and focus group discussion transcripts. Uploading all transcripts for open availability would compromise our ability to fully mask participant details. However, we are happy to share anonymized portions of these transcripts upon reasonable request.

Contributor statement

KS, SC , OU, DM and AEL conceptualized and designed the study. MS, DG, BM and NC conducted the data collection and preliminary data analysis through daily analytic debriefs. KS and OU led and managed the data collection and analysis, including the coding and thematic synthesis. KS drafted the manuscript and revised it based on critical and

For peer review only - http://bmjopen.bmj.com/site/about/guidelines.xhtml

substantive input from SC, OU, AEL, MS, BM, DM and NC. All authors agree to be accountable for all aspects of the work related to accuracy and integrity.

Funding statement

This work was supported by the Bill and Melinda Gates Foundation grant number OPP1179252.

Works Cited

1. WHO. Framework and Standards for Country Health Information Systems, 2nd Edition. Geneva, Switzerland; 2012.
2. WHO. Health Metrics Network Framework and Standards for Country Health Information Systems. 2008.
3. Al-Shorbaji N, Geissbuhler A. Establishing an evidence base for e-health: the proof is in the pudding. Bull World Health Organ. 2012;90:322.
4. WHO. Resolution WHA58.28. eHealth. WHA58/2005/REC/1 Fifty-eighth World Health Assembly, Geneva, 16–25 May 2005. Annex. Resolutions and decisions. Geneva; 2005.
5. Lewis T, Synowiec C, Lagomarsino G, Schweitzer J. E-health in low- and middle-income countries: findings from the Center for Health Market Innovations. Bull World Heal Organ. 2012;90:332–40.
6. Neupane S, Odendaal W, Friedman I, Jassat W, Schneider H, Doherty T. Comparing a paper based monitoring and evaluation system to a mHealth system to support the national community health worker programme, South Africa: an evaluation. BMC Med Inform Decis Mak. 2014 Dec 9;14(1):69.
7. Tamrat T, Kachnowski S. Special Delivery: An Analysis of mHealth in Maternal and Newborn Health Programs and Their Outcomes Around the World. Matern Child Health J. 2012 Jul 19;16(5):1092–101.
8. Charanthimath U, Katageri G, Kinshella M-LW, Mallapur A, Goudar S, Ramadurg U, et al. Community Health Worker Evaluation of Implementing an mHealth Application to Support Maternal Health Care in Rural India. Front Glob Women's Heal 2021 Sep 1;2.
9. Källander K, Tibenderana JK, Akpogheneta OJ, Strachan DL, Hill Z, Asbroek A, et al. Mobile health (mhealth) approaches and lessons for increased performance and retention of community health workers in lowand middle-income countries: A review. J Med Internet Res. 2013;15(1).
10. Chamberlain S, Dutt P, Godfrey A, Mitra R, LeFevre AE, Scott K, et al. Ten lessons learnt: scaling and transitioning one of the largest mobile health communication programmes in the world to a national government. BMJ Glob Heal. 2021 Jul 26;6(Suppl 5):e005341.
11. Scott K, Ummer O, Shinde A, Sharma M, Yadav S, Jairath A, et al. Another voice in the crowd: The challenge of changing family planning and child feeding practices through mHealth messaging in rural central India. BMJ Glob Heal. 2021;6.
12. Bashingwa JJH, Mohan D, Chamberlain S, Arora S, Mendiratta J, Rahul S, et al.

For peer review only - http://bmjopen.bmj.com/site/about/guidelines.xhtml

Assessing exposure to Kilkari: A big data analysis of a large maternal mobile messaging service across 13 states in India. BMJ Glob Heal. 2021;6.

13. Gera R, Muthusamy N, Bahulekar A, Sharma A, Singh P, Sekhar A. An in-depth assessment of India's Mother and Child Tracking System (MCTS) in Rajasthan and Uttar Pradesh. BMC Health Serv Res. 2015;1–14.
14. Balakrishnan R, Gopichandran V, Chaturvedi S, Chatterjee R, Mahapatra T, Chaudhuri I. Continuum of Care Services for Maternal and Child Health using mobile technology – a health system strengthening strategy in low and middle income countries. BMC Med Inform Decis Mak. 2016;16(84):1–8.
15. SRS. Special Bulletin on Maternal Mortality In India 2016-18. New Delhi; 2020.
16. MoHFW India. National Family Health Survey 4 (2015-2016): State Fact Sheet Rajasthan. Mumbai; 2017.
17. MoHFW India. National Family Health Survey - 4 (2015-16); State Fact Sheet Madhya Pradesh. 2017.
18. GoI. RCH Application and ANMOL: Data Elements, Validation, Logic and Tool Tip Document, V 2.0. 2016.
19. Attride-Stirling J. Thematic Networks: an analytic tool for qualitative research. Qual Res. 2001;1(3):385–405.
20. Mukherjee A. Who's data is this anyway? Understanding relationship between power, empowerment and technology: Case from Mother and Child Tracking System (MCTS) in India. Proceedings of the 9 th Health Informatics in Africa Conference HELINA'14 PART 2. Hamburg, Germany; 2014.
21. NHSRC and Taurus Glocal Consult. Study of Public Health IT Systems in India. New Delhi, India; 2011.
22. Gera R, Muthusamy N, Bahulekar A, Sharma A, Singh P, Sekhar A. An in-depth assessment of India's Mother and Child Tracking System (MCTS) in Rajasthan and Uttar Pradesh. BMC Health Serv Res. 2015;15:315.
23. Mphatswe W, Mate K, Bennett B, Ngidi H, Reddy J, Barker P, et al. Improving public health information: a data quality intervention in KwaZulu-Natal, South Africa. Bull World Health Organ. 2012 Mar 1;90(3):176–82.
24. The Earth Institute. Pregnancy Child Tracking and Health Services Management System (PCTS) Gap Analysis -- Rajasthan. Mumbai; 2014.
25. Levy AG, Scherer AM, Zikmund-Fisher BJ, Larkin K, Barnes GD, Fagerlin A. Prevalence of and Factors Associated With Patient Nondisclosure of Medically Relevant Information to Clinicians. JAMA Netw Open. 2018 Nov 30;1(7):e185293.
26. Agaku IT, Adisa AO, Ayo-Yusuf OA, Connolly GN. Concern about security and privacy, and perceived control over collection and use of health information are related to withholding of health information from healthcare providers. J Am Med Informatics Assoc. 2014 Mar 1;21(2):374–8.
27. Dickinson FM, McCauley M, Madaj B, Van Den Broek N. Using electronic tablets for data collection for healthcare service and maternal health assessments in low resource settings: Lessons learnt. BMC Health Serv Res. 2019;19(1).
28. Gopalakrishnan L, Diamond-Smith N, Avula R, Menon P, Fernald L, Walker D, et al. Association between supportive supervision and performance of community health workers in India: a longitudinal multi-level analysis. Hum Resour Health. 2021 Dec 27;19(1):145.
29. Sadiq Umar A, Mohammed Bello I, Chukwudi Okeibunor J, Mkanda P, Ubong Akpan G,

For peer review only

Manyanya D, et al. The Effect of Real Time Integrated Supportive Supervision Visits on the Performance of Health Workers in Zambia. J Immunol Sci. 2021;Special Issue(2):120–6.
30. WHO. Training for mid-level managers (MLM). Module 4. Supportive Supervision. Geneva, Switzerland; 2008.

For peer review only

For peer review only

For peer review only - http://bmjopen.bmj.com/site/about/guidelines.xhtml

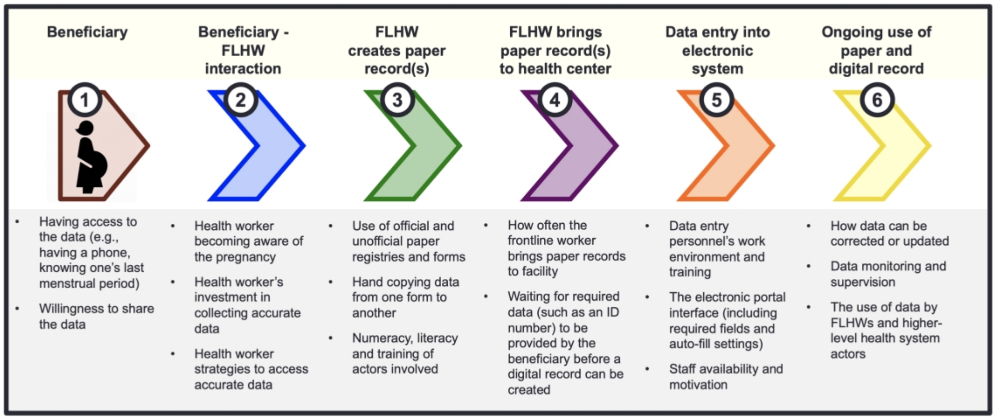

Figure 1. Data flow framework for electronic health record systems

492x206mm (144 x 144 DPI)

Annex 1. Acronyms and key terms used

ASHA	Accredited Social Health Activist (ASHA Sahyogini in Rajasthan), a female community health worker
AWW or Anganwadi	Anganwadi Worker, a female community nutrition and preschool functionary
ANM	Auxiliary nurse midwife, a female frontline health worker who provides outreach and some clinical maternal and child health care
DEO	Data entry operator
FGD	Focus group discussion
FLHW	Frontline health workers
HIS	Health information systems
ICT	Information communication technologies
IDI	In-depth interview
MCP card	Mother Child Protection card, a health tracking and information card filled out by the frontline worker and kept by the pregnant woman
MCTS	Maternal and Child Health Tracking System / Reproductive and Child Health system. MCTS is an initiative of Ministry of Health & Family Welfare to leverage information technology for ensuring delivery of full spectrum of healthcare and immunization services to pregnant women and children up to 5 years of age. It facilitates and monitors service delivery and also establishes a two way communication between the service providers and beneficiaries (Ref: Digital India, MoHFW, http://nrhm-mcts.nic.in/Home.aspx [accessed 3 Dec 2021])
mHealth	Mobile health, use of mobile and wireless technologies to support the achievement of health objectives
MO	Medical Officer (a doctor)
PCTS	Pregnancy, Child Tracking, and Health Services Management System (Rajasthan's version of MCTS, which also syncs with MCTS)
RCH portal	Reproductive and Child Health portal. RCH (an upgraded version of MCTS) has been designed for early identification and tracking of the individual beneficiary throughout the reproductive lifecycle. While MCTS tracks every pregnancy, RCH portal tracks a woman throughout her reproductive life cycle, thus making available information on obstetric history. (Ref: RCH Portal & Allied Initiatives of MoHFW, 2018, https://icds-wcd.nic.in/nnm/Events/TechThon/ExistingTechnologyPlatform_for_HealthSchemes_MoHFW-28-06-2018.pdf [accessed 3 Dec 2021])

For peer review only

COREQ (COnsolidated criteria for REporting Qualitative research) Checklist

A checklist of items that should be included in reports of qualitative research. You must report the page number in your manuscript where you consider each of the items listed in this checklist. If you have not included this information, either revise your manuscript accordingly before submitting or note N/A.

Topic	Item No.	Guide Questions/Description	Reported on Page No.
**Domain 1: Research team and reflexivity**			
Personal characteristics			
Interviewer/facilitator	1	Which author/s conducted the interview or focus group?	
Credentials	2	What were the researcher's credentials? E.g. PhD, MD	
Occupation	3	What was their occupation at the time of the study?	
Gender	4	Was the researcher male or female?	
Experience and training	5	What experience or training did the researcher have?	
Relationship with participants			
Relationship established	6	Was a relationship established prior to study commencement?	
Participant knowledge of the interviewer	7	What did the participants know about the researcher? e.g. personal goals, reasons for doing the research	
Interviewer characteristics	8	What characteristics were reported about the inter viewer/facilitator? e.g. Bias, assumptions, reasons and interests in the research topic	
**Domain 2: Study design**			
Theoretical framework			
Methodological orientation and Theory	9	What methodological orientation was stated to underpin the study? e.g. grounded theory, discourse analysis, ethnography, phenomenology, content analysis	
Participant selection			
Sampling	10	How were participants selected? e.g. purposive, convenience, consecutive, snowball	
Method of approach	11	How were participants approached? e.g. face-to-face, telephone, mail, email	
Sample size	12	How many participants were in the study?	
Non-participation	13	How many people refused to participate or dropped out? Reasons?	
Setting			
Setting of data collection	14	Where was the data collected? e.g. home, clinic, workplace	
Presence of non-participants	15	Was anyone else present besides the participants and researchers?	
Description of sample	16	What are the important characteristics of the sample? e.g. demographic data, date	
Data collection			
Interview guide	17	Were questions, prompts, guides provided by the authors? Was it pilot tested?	
Repeat interviews	18	Were repeat inter views carried out? If yes, how many?	
Audio/visual recording	19	Did the research use audio or visual recording to collect the data?	
Field notes	20	Were field notes made during and/or after the inter view or focus group?	
Duration	21	What was the duration of the inter views or focus group?	
Data saturation	22	Was data saturation discussed?	
Transcripts returned	23	Were transcripts returned to participants for comment and/or	

For peer review only

Topic	Item No.	Guide Questions/Description	Reported on Page No.
		correction?	
**Domain 3: analysis and findings**			
Data analysis			
Number of data coders	24	How many data coders coded the data?	
Description of the coding tree	25	Did authors provide a description of the coding tree?	
Derivation of themes	26	Were themes identified in advance or derived from the data?	
Software	27	What software, if applicable, was used to manage the data?	
Participant checking	28	Did participants provide feedback on the findings?	
Reporting			
Quotations presented	29	Were participant quotations presented to illustrate the themes/findings? Was each quotation identified? e.g. participant number	
Data and findings consistent	30	Was there consistency between the data presented and the findings?	
Clarity of major themes	31	Were major themes clearly presented in the findings?	
Clarity of minor themes	32	Is there a description of diverse cases or discussion of minor themes?	

Developed from: Tong A, Sainsbury P, Craig J. Consolidated criteria for reporting qualitative research (COREQ): a 32-item checklist for interviews and focus groups. *International Journal for Quality in Health Care*. 2007. Volume 19, Number 6: pp. 349 – 357

Once you have completed this checklist, please save a copy and upload it as part of your submission. DO NOT include this checklist as part of the main manuscript document. It must be uploaded as a separate file.

For peer review only

BMJ Open

At the frontlines of digitization: a qualitative study on the challenges and opportunities in maintaining accurate, complete, and timely digital health records in India's government health system

Journal:	*BMJ Open*
Manuscript ID	bmjopen-2021-051193.R2
Article Type:	Original research
Date Submitted by the Author:	13-Jan-2022
Complete List of Authors:	Scott, Kerry; Johns Hopkins University Bloomberg School of Public Health<br>Ummer, Osama; Oxford Policy Management; BBC Media Action, Research and Learning<br>Chamberlain, Sara; BBC Media Action, BBC Media Action, India; BBC Media Action, Asia<br>Sharma, Manjula; Independent researcher<br>Gharai, Dipanwita; Oxford Policy Management<br>Mishra, Bibha; Oxford Policy Management<br>Choudhury, Namrata; Jawaharlal Nehru University<br>Mohan, Diwakar; Johns Hopkins University Bloomberg School of Public Health<br>LeFevre, Amnesty; University of Cape Town
**Primary Subject Heading**:	Health services research
Secondary Subject Heading:	Health informatics, Global health, Qualitative research
Keywords:	Public health < INFECTIOUS DISEASES, Health informatics < BIOTECHNOLOGY & BIOINFORMATICS, Information management < BIOTECHNOLOGY & BIOINFORMATICS, Information technology < BIOTECHNOLOGY & BIOINFORMATICS, HEALTH SERVICES ADMINISTRATION & MANAGEMENT

SCHOLARONE™
Manuscripts

I, the Submitting Author has the right to grant and does grant on behalf of all authors of the Work (as defined in the below author licence), an exclusive licence and/or a non-exclusive licence for contributions from authors who are: i) UK Crown employees; ii) where BMJ has agreed a CC-BY licence shall apply, and/or iii) in accordance with the terms applicable for US Federal Government officers or employees acting as part of their official duties; on a worldwide, perpetual, irrevocable, royalty-free basis to BMJ Publishing Group Ltd ("BMJ") its licensees and where the relevant Journal is co-owned by BMJ to the co-owners of the Journal, to publish the Work in this journal and any other BMJ products and to exploit all rights, as set out in our licence.

The Submitting Author accepts and understands that any supply made under these terms is made by BMJ to the Submitting Author unless you are acting as an employee on behalf of your employer or a postgraduate student of an affiliated institution which is paying any applicable article publishing charge ("APC") for Open Access articles. Where the Submitting Author wishes to make the Work available on an Open Access basis (and intends to pay the relevant APC), the terms of reuse of such Open Access shall be governed by a Creative Commons licence – details of these licences and which Creative Commons licence will apply to this Work are set out in our licence referred to above.

Other than as permitted in any relevant BMJ Author's Self Archiving Policies, I confirm this Work has not been accepted for publication elsewhere, is not being considered for publication elsewhere and does not duplicate material already published. I confirm all authors consent to publication of this Work and authorise the granting of this licence.

For peer review only

At the frontlines of digitization: a qualitative study on the challenges and opportunities in maintaining accurate, complete, and timely digital health records in India's government health system

Authors: Kerry Scott[1], Osama Ummer[2], Sara Chamberlain[3], Manjula Sharma[4], Dipanwita Gharai[2], Bibha Mishra[2], Namrata Choudhury[5], Diwakar Mohan[1] and Amnesty LeFevre[1,6]

[1] Department of International Health, Johns Hopkins Bloomberg School of Public Health, 615 N. Wolfe St, Baltimore, Maryland, USA
[2] Oxford Policy Management-Delhi, 4/6 First Floor, Siri Fort Institutional Area, New Delhi, Delhi 110049, India
[3] BBC Media Action, India, Old Fort Saket District Mall, Saket District Centre, Sector 6, Pushp Vihar, New Delhi, Delhi 110017, India
[4] Independent researcher, New Delhi, India
[5] Centre for the Study of Law and Governance, Jawaharlal Nehru University
[6] School of Public Health and Family Medicine, University of Cape Town, Cape Town, South Africa

Corresponding author: Kerry Scott, 615 N. Wolfe St, Baltimore, MD, 21205, kscott26@jhu.edu, 647-675-7956

For peer review only

Abstract

Objectives: To understand factors underpinning the accuracy and timeliness of mobile phone numbers and other health information captured in India's government registry for pregnant and postpartum women. Accurate and timely registration of mobile phone numbers is necessary for beneficiaries to receive mHealth services.

Setting: Madhya Pradesh and Rajasthan states in India at the community, clinical, and administrative levels of the health system.

Participants: Interviews (n=59) with frontline health workers (FLHWs), data entry operators, and higher level officials. Focus group discussions (n=12) with pregnant women to discuss experiences with sharing data in the health system. Observations (n=9) of interactions between stakeholders for data collection and the process of data digitization.

Primary and secondary outcome measures: Thematic analysis identified how key actors experienced the data collection and digitization process, reasons for late or inaccurate data, and mechanisms that can bolster timeliness and accuracy.

For peer review only - http://bmjopen.bmj.com/site/about/guidelines.xhtml

Results: Pregnant women were comfortable sharing mobile numbers with health workers but many were unaware that their data moved beyond their FLHW. FLHWs valued knowing up-to-date beneficiary mobile numbers but felt little incentive to ensure accuracy in the digital records system. Delays in registering pregnant women in the online portal were attributed to slow movement of paper records into the digital system and difficulties in gathering required documents from beneficiaries. Data, including women's phone numbers, were hand written and copied multiple times by beneficiaries and health workers with variable literacy. Supervision tended to focus on completeness rather than accuracy. Health system actors noted challenges with the digital system but valued the broader project of digitization.

Conclusions: Increased focus on training, supportive supervision, and user-friendly data processes that prioritize accuracy and timeliness should be considered. These inputs can build upon existing positive patient-provider relationships and health system actors' enthusiasm for digitization.

Strengths and limitations of this study

- This study was strengthened by the use of multiple research methods (interviews, focus groups and observation) and engagement of multiple frontline stakeholders: the women whose data is entered into the system, the frontline health workers who care for them and create the initial (paper) records, the data entry operators who digitize these records, and the managers, administrators and leaders who supervise these processes.

- Frontline health workers and data entry operators were aware that their work was being observed and thus may have minimized or hidden unauthorized behavior, such as shortcuts to speed up data entry or unsanctioned task-shifting.

- Data collection occurred just prior to the launch of a new digitization strategy in some (but not all) regions, wherein frontline health workers would be given tablets and asked to directly digitize patient health data; thus some of the challenges identified in this study may have self-resolved due to this new (shorter) dataflow pathway, and new challenges to accuracy, timeliness and completeness may have arisen.

Introduction

Health information systems (HIS) capture data on patients and their contacts with routine health services. Data collected about patients includes clinical content, such as weight and

For peer review only - http://bmjopen.bmj.com/site/about/guidelines.xhtml

blood pressure, and non-clinical content, such as their address, patient identification code, and phone number. HIS support responsive policymaking, resource allocation, routine service delivery, and health systems accountability.[1] The achievement of these functions is largely dependent on the accuracy, completeness and timeliness of the data in the health information system.[2] Information communication technologies (ICTs) are increasingly being used to improve the core functions of HIS through digitization, wherein patient data is entered into a computer system rather than maintained in handwritten records.[3–5] ICTs for health information systems have great potential to strengthen health systems by streamlining data collection and entry, accelerating the transmission and analysis of data, instituting validity checks, and increasing frontline worker access to a range of clinical and administrative support services. [6,7] [8]

The digitization of health information systems has occurred concurrently with increasing mobile phone penetration at a population level and, in turn, an increase in mobile health (mHealth) initiatives. mHealth programs can transmit health-related information, send reminders, or solicit patient feedback on services.[9] To attain high population level coverage at scale, mHealth programs often rely on beneficiary mobile numbers and other clinical and non-clinical data that have been collected through government health information systems. The accuracy, completeness, and timeliness of records in government systems can directly affect the capacity of mHealth programs to reach target populations at the right timepoints.

The Government of India's Kilkari program is one example of an mHealth service that relies on the quality and timeliness of mobile phone numbers and other data collected in the government's health registries in order to reach beneficiaries. Developed and scaled by BBC Media Action in collaboration with the Indian Ministry of Health and Family Welfare, Kilkari is the world's largest maternal mobile messaging initiative, having reached 10 million subscribers in 13 states by December 2018.[10] It delivers maternal, child, and reproductive health information content through up to 72 once-weekly outgoing pre-recorded calls. Beneficiaries (pregnant or postpartum women and their husbands)[11] are subscribed to Kilkari based on the mobile number captured in governmental HIS registries called, depending on the state, the Maternal and Child Health Tracking System (MCTS) or Reproductive and Child Health (RCH) system.[10] The gestational age estimate or child's date of birth is used to determine the appropriate Kilkari messages to send based on the stage in pregnancy or child's age.

Missing and incorrect data in MCTS/RCH reduces the number of beneficiaries who can be exposed to Kilkari. In 2018, one quarter of the unique mobile numbers registered for Kilkari were never reached[12] due to a number of factors including inaccurate or out-of-date mobile numbers and the phone being switched off or out of network. A 2015 study[13] found that one-third of women's profiles in the government registry in Rajasthan and two-thirds in Uttar Pradesh were incomplete. Data delays and late care-seeking impede overall exposure to Kilkari. Kilkari exposure can begin in second trimester of pregnancy, however only 31% of subscribers answered their first call during this period.[12] Over half (58%) answered their first call after they give birth, which means they missed at least one-third of Kilkari's content.[12] Research from Bihar documented an average delay of 72 days between service delivery and being registered in MCTS.[14]

For peer review only

For peer review only - http://bmjopen.bmj.com/site/about/guidelines.xhtml

In order to understand why Kilkari was not reaching every pregnant woman as soon as possible (e.g., in the second trimester) we conducted a qualitative examination of frontline barriers to the accurate, complete, and timely capture of mobile phone numbers and other data in MCTS/RCH. We explored beneficiary perceptions of providing mobile phone numbers to health care providers, and frontline health worker (FLHW) experiences with the digital health record system broadly, and capturing mobile numbers specifically. Study findings will inform health systems in India and beyond as they move towards ICT-enabled strategies to bolster the quality of health information systems.

Methods

Study setting

This qualitative study took place in Madhya Pradesh and Rajasthan, two large Hindi speaking states in central and western India, respectively. These states have high burdens of maternal and child mortality, significant gender gaps around technology access and literacy, and sub-optimal maternal healthcare services (table 1).

Table 1. Social and health indicators, Rajasthan and Madhya Pradesh

Indicator	Rajasthan	Madhya Pradesh
Population	77 million	82 million
Maternal mortality ratio (deaths per 100,000)[15]	164	173
Under five mortality (deaths per 1000 live births) [16,17]	51	65
Literacy[16,17]		
• Female	57%	59%
• Male	85%	82%
Mobile phone access[16,17]		
• Household ownership	94%	84%
• Female access	41%	29%
Maternal health care[16,17]		
• Pregnant women attended antenatal care in first trimester	63%	53%
• Received recommended four antenatal care visits	39%	36%
• Gave birth in a health facility	84%	81%
• Received postnatal health check within 2 days of birth	64%	55%
• Registered pregnancies for which the mother received Mother and Child Protection (MCP) card	92%	92%

Both Madhya Pradesh and Rajasthan moved to digital health records in the late 2000s, however there are important differences between the states, in terms of the digital programs and processes implemented (Table 2). In 2016, Madhya Pradesh transitioned from MCTS to RCH. RCH added urban coverage, an initial registration of all “eligible couples” (married couples of reproductive age) who would then be linked to pregnancy tracking when a pregnancy occurred, and the creation of village profiles.[18] Furthermore, RCH expanded the data elements collected from 111 (in MCTS) to 247 to include abortion

For peer review only - http://bmjopen.bmj.com/site/about/guidelines.xhtml

tracking, beneficiary bank account and identification details (including the Aadhaar national identification number), additional details about the pregnant woman's antenatal care, infant feeding practice, and the child's immunization records for his or her first five years.[18] Mobile phone numbers were collected in MCTS and continued to be in RCH. In contrast to Madhya Pradesh, the Rajasthan state government did not adopt RCH but instead retained its state-level version of MCTS, called the Pregnancy, Child Tracking, and Health Services Management System (PCTS), which syncs with MCTS. Annexure 1 contains an explanation of key acronyms and terms.

Table 2. Comparing Rajasthan and Madhya Pradesh's digital HIS

Parameter	Rajasthan	Madhya Pradesh
HIS system currently in use	Pregnancy Child Tracking and Health Services Management System (PCTS), a state-specific system that syncs with MCTS	Reproductive and Child Health (RCH), an expanded and upgraded version of MCTS
Timeline	Adopted PCTS in 2008 (first state in India to launch an electronic health records system) and has not changed to RCH	Adopted MCTS in 2009 and changed to RCH in 2016
Who collects HIS data at the frontline?	Three frontline workers: community health worker called the Accredited Social Health Activist (ASHA Sahyogini in Rajasthan), community nutrition and preschool worker called anganwadi & auxiliary nurse midwife (ANM)	Two frontline workers: ASHA & ANM. The anganwadi worker is not involved.
Feedback mechanisms built into the digital record system	Creates a workplan for the ANM telling her who is due for antenatal care, delivery, postnatal care as well as listing drop outs; sends SMS alerts to beneficiaries (program called Swasthya Sandesh Sewa)	Creates a workplan for the ANM telling her who is due for antenatal care, delivery, and postnatal care, as well as listing drop outs; sends SMS alerts to beneficiaries and also to health functionaries at different levels
Paper forms involved in data collection for pregnancies	***1) Pregnant women's antenatal care registry form***: Paper where up to five new pregnancies being managed by the frontline worker can be listed with details of first antnatal care visit. This form is filled in by the ASHA/ANM and passed to the data entry operator for entry into PCTS. ***2) Mamta card***: Card filled by ANM/ASHA; stays with the pregnant woman ***3) Reproductive and Child Health (RCH) register***: Book kept with the ANM or at the health center; all pregnancies under the ANM are in this book. It is passed to the data entry operator for data entry. ***4) Personal ANM diary***: Unofficial paper book that ANMs fill (because RCH register is sometimes too big to	***1) RCH form***: One form per woman, filled by ASHA/ANM and given to the data entry operator. Meant to travel back and forth between ANM and data entry operator as the woman proceeds through her antenatal care visits and delivery. ***2) Mother Child Protection (MCP) card***: Card filled by ANM/ASHA; stays with the pregnant woman ***3) RCH register:*** Book kept with the ANM or at the health center; all pregnancies under the ANM are in this book. It is passed to the data entry operator for data entry. ***4) Personal ANM diary***: Unofficial paper book that ANMs fill (because RCH register is sometimes too big to bring to the village or is with the data entry operator) ***5) Personal ASHA diary***: Official paper book that the ASHA fills

For peer review only - http://bmjopen.bmj.com/site/about/guidelines.xhtml

Parameter	Rajasthan	Madhya Pradesh
	bring to the village or is with the data entry operator)<br>***5) Personal ASHA diary***: Official paper book that the ASHA fills	

The states thus enable us to examine data systems in a more typical case (Madhya Pradesh), which, like most large Indian states, recently moved from MCTS to RCH, and an outlier case (Rajasthan) which has retained a tailor-made electronic record system since the beginning of digitization.

Data collection

Five experienced qualitative researchers with master's level social science degrees (authors OU (male) MS, DG, BM and NC (female)) conducted in depth interviews (n=59), focus groups (n=12) and observation of data collection and entry (n=9) in Rajasthan and Madhya Pradesh in September and October 2018 (table 3). All researchers were trained over a one-week period, which included pilot testing the FLHW interview guide. In each state, we selected one district and two blocks within that district with high levels of female phone ownership to explore barriers to capturing women's mobile phone numbers in MCTS/RCH. We sampled government health system actors who were involved in MCTS/RCH at the state, district, block and frontline levels, including medical officers, data entry officers and frontline providers, as well as with women who recently interacted with government healthcare providers in situations where they were asked to register their mobile numbers in MCTS/RCH (table 3).

Table 3. Respondent sample

Respondent type	Respondent profile	MP	RJN	N
In depth interviews (IDIs)		Number of IDIs		
State level stakeholders	Senior government employees of the department of health and family welfare who are in charge of data	3	1	4
District stakeholders	Community mobilizers, District Programme Manager, District Community Mobilizer, District Monitoring and Evaluation Officer, District Nodal Officer	3	4	7
Medical officers (MOs) at primary health centers	Doctors (allopathic, homeopathic or ayurvedic), 5.5 years training	2	4	6
Data entry operators (DEOs)	Information technologists with undergraduate level education	4	6	10
Auxiliary nurse midwives (ANMs)	Female maternal and child health worker with 1.5 years training (6 months midwifery focused)	10	9	19
Accredited Social Health Activist (ASHA) community health worker	Female volunteer community health worker, received incentive based remuneration, initial 24 days training and periodic week-long additional training	3	5	8
Other stakeholders at the PHC and block level	Block Programme Manager, Multipurpose Health Supervisor, Primary Health Centre Supervisor	2	3	5
Total IDIs		27	32	59
Focus Group Discussions (FGDs)		Number of FGDs		
Beneficiaries	Pregnant and post-partum women with mobile phones who recently interacted with health system actors for MCTS/RCH registration	6	6	12

For peer review only - http://bmjopen.bmj.com/site/about/guidelines.xhtml

Observation		Number of observations		
Beneficiary – FLHW interaction	Observation of beneficiary – FLHW interaction (antenatal or post-partum) where MCTS/RCH data recorded	1	1	2
Data entry into electronic system	Observation of data entry into electronic system	3	4	7
Total observations		4	5	9

The respondents were approached through their government health facility. A research team member contacted potential respondents by phone or face-to-face and explained the study, and that the team was from a Delhi-based company and had governmental approval, then invited them for a face to face meeting to learn more and, if they agreed, to participate. The study information and informed consent was read to each potential participant and then summarized in conversational language to ensure comprehension. While all the health facility staff approached for the study agreed to participate while three women invited to attend focus groups declined, siting responsibilities at home. The interviews took about an hour and the focus group discussions took about an hour and a half; all were conducted in health faculties and audio recorded and detailed notes were taken. When curious onlookers came over during focus groups and interviews, another researcher politely asked them to move on. If any supervisors, patients or family members stopped by to speak to the respondents during the interviews or focus groups the research paused until privacy was restored.

FGDs ranged from four to 10 participants (mean 7.6 participants). The focus group compositions broadly reflected local demographics. They included women with a wide range of education levels (from no education to master's degrees), castes (most included a mix of women from marginalized schedule caste and schedule tribal groups as well as women from "other backwards castes" and general caste groups), and religions (three included some Hindu and some Muslim women, while the remainder were all Hindu). Most women were homemakers, while a sizable minority worked as agricultural farmers and labourers, and also included students, tailors, shopkeepers and bangle/jewelry saleswomen.

Interviews and FGDs were conducted using semi structured guides that explored a range of domains around sharing, documenting, inputting, and using data, with a focus on mobile numbers (see supplementary file 1 for examples of the guides). We explored potential drivers of inaccuracies and delays by asking about late pregnancy identification, FLHW work environment, and the relationship between beneficiaries and FLHWs. In the interviews with health system actors we also explored each step of a detailed description of data flow (Fig 1) from when a pregnant woman first interacts with a FLHW until her health information is entered into the online portal, and beyond to understand perceptions on the use of this data.

Ethics approval

This study was approved by the institutional ethics review boards at Sigma, Delhi, India (10041/IRB/D/17-18) and JHU, Baltimore, USA (00008360). All respondents provided informed oral consent.

For peer review only - http://bmjopen.bmj.com/site/about/guidelines.xhtml

Data analysis

Daily debriefs enabled the team to share emergent findings, refine the focus of their probing for the next day's data collection, and identify areas of saturation. The audio files were transcribed and translated into English. Data were coded in Dedoose by OU and KS, using principles of thematic network analysis.[19] A coding framework was developed that consisted of emergent codes on specific reasons for inaccurate and delayed data, that were then grouped according to an overarching data flow framework (Fig 1). For instance, we created a code cluster for late antenatal care registration, which included codes to be applied to text describing when and how pregnancies come to the FLHW's attention, when pregnancies were entered into the online portal, reasons FLHWs become aware of pregnancies late (after the first trimester), and the implications of a woman's choice of the public or private sector for antenatal care on timeliness of registration. After coding, we read the text excerpts that had been tagged for specific codes to identify how key actors experience of the data collection and digitization process, reasons for late or inaccurate data, and mechanisms that can bolster timeliness and accuracy.

Conceptual framework

While we initially set out to understand inaccuracies and delays in entering mobile phone numbers into the pregnancy registry, it became clear that this one piece of data could not be separated from the broader data collection process at the frontlines of government health service provision. We thus examined mobile phone number data collection and digitization within the context of an overarching health system data flow framework (Fig 1). This framework identifies six components that enable the creation and movement of health data, including mobile phone numbers, from beneficiaries through FLHWs to electronic data entry and onward.

Figure 1. Data flow framework for electronic health record systems

The first component, "beneficiary," describes women's access to the data required for documentation (e.g., whether they have a mobile phone number to provide), as well as their attitudes towards sharing this data with government health system functionaries (e.g., their willingness to provide their mobile numbers when asked). The second component considers the beneficiary-FLHW interactions that initiate the government health system's awareness of a health event. In this section we consider how and when FLHWs become aware of a new pregnancy, the value that FLHWs place on collecting beneficiary data, and the strategies used to collect data. The third component involves the creation of initial paper records. In this section we consider the various official and unofficial forms and registers where data is recorded, the health worker's literacy and numeracy if data is copied from paper form to paper form, and potential delays between a health worker interacting with a beneficiary and a paper record being created. The fourth component considers the process through which a paper health record reaches the site of digitization. We consider how often the FLHW visits the digitization site and whether the FLWH waits to bring paper records for digitization until all mandatory fields have been filled out. While steps three and

For peer review only - http://bmjopen.bmj.com/site/about/guidelines.xhtml

four may be dropped as FLHWs directly create digital records themselves, in many health systems in lower resource settings, paper records are still created during outreach service provision. The fifth component is the time when data is digitized and considers the data entry personnel's work environment, training, the electronic portal interface, and staffing considerations. The final component examines the ongoing use of data through paper forms and online systems. Here we consider how data can be corrected or updated, data monitoring and supervision, and the use of data by FLHWs and higher level health system actors.

Patient and Public Involvement

The research was shaped by health system actor priorities, experiences and preferences through iterative probing and flexibility within our research domains. Results were disseminated to Government of India stakeholders but not to research participants due to the policy-level implications of our findings.

Findings

Our findings are organized conceptually according to the six components in the general data flow framework. For each step, we provide a description of how this aspect of the data system is experienced by the actors involved, and identify barriers and facilitators to timeliness and accuracy.

Level 1. Beneficiary

Women faced barriers to accessing phones and services that generate the data required by MCTS/RCH

Women who did not have their own personal mobile phones could not provide their number. Many women lived in households with phones so could provide their husband's phone number or the number for a shared phone. Women living in households without a phone could provide a neighbor's phone number or a FLHW's number.

> R: Some women have their husband's mobile number. In some cases both husband and wife don't have mobile phones. Some are from poor families where both of them don't have mobile phones.
> I : So, what do you do in such situation?
> R: Either we take a relative's phone number or a neighbor's. […] If any laborer is there who does labor work how will he have mobile phone? Even if it's there then her husband must have taken it while going for labor work.
> (MP_IDI_FLHW_06, ANM)

Additional data required from beneficiaries included their bank account numbers and branch names (for both the beneficiary and her husband), national identification (Aadhaar), ration card number, and state identification numbers (Bhamashah state health insurance in

For peer review only - http://bmjopen.bmj.com/site/about/guidelines.xhtml

Rajasthan and Samagra in Madhya Pradesh). FLHWs explained that women often did not have all of these items or did not know this information. ASHAs were tasked with following up with women to encourage them – or at times personally help them – open bank accounts, register for identification numbers, and provide photocopies of this information.

> Like the [bank] account number, Aadhaar number we get them very rarely. We have to with the ASHA then go again to get information. We fill the form and submit it and it's fed in. If half the information remains incomplete then we can't do the RCH. (MP_IDI_FLHW_01, ANM)

Women were generally comfortable sharing data with frontline health workers

Women and FLHWs overwhelmingly reported that beneficiaries with access to a mobile phone shared their mobile number with their ASHA or ANM without hesitation and without questioning. They explained that women trusted FLHWs and felt that the number would only be used to help them, such as to convey important information about health services or financial benefits through government programs.

> They are observing me from 20 years so they are aware that madam will not misuse the number so they never deny giving number (RJN_IDI_FLHW_25, ANM)

> [Frontline health workers take our mobile number] so that they can give us a phone call next time to call us whenever vaccination is to be given. That's why. (RJN_FGD_BENEF_07)

> They are aware that the mobile number is essential as when the money will come in the bank account then they will get the message on mobile. We take the mobile number for their comfort only as we can call them as required. […] And calls also come on their mobile who tells them about the health. (RJN_IDI_FLHW_27, ASHA)

There were exceptions to this predominantly positive view of sharing mobile numbers with health workers. FLHWs and beneficiaries reported that a very small minority of women resisted sharing their numbers with them because of concern about receiving calls from strangers or because they had hidden their mobile phone.

In Madhya Pradesh, most women thought that only the ASHA had their number. When told that their numbers were passed onward from the ASHA to the ANM, women were unconcerned. In Rajasthan, women were generally aware that their mobile numbers were collected for use beyond beneficiary-ASHA communication. They clearly explained that their mobile number was linked to their receipt of financial benefits. While most women in Rajasthan said the only calls they received from government actors were from their ASHA, calling them for checkups, a few received informational calls from the government of India, which were likely Kilkari messages, and one said that the government would use their mobile numbers to call them and check "whether the facilities which government is providing are reaching us or not" (RJN_FGD_BENEF_09).

For peer review only - http://bmjopen.bmj.com/site/about/guidelines.xhtml

Level 2. FLHW-beneficiary interaction

Early identification of most pregnancies – with notable exceptions

Digital records of pregnancies can only be created after the government health system functionaries identify a pregnant woman. ASHAs are a pregnant women's first point of contact with the government health system. ANMs rely on ASHAs to identify pregnant women and encourage them to meet the ANM for antenatal care. Beneficiaries and FLHWs reported that ASHAs were notified within the first four months about almost all pregnancies.

However, FLHWs noted that women living in remote communities, migrants and less educated women, may delay seeking antenatal care due to "carelessness" (RJN_IDI_FLHW_27, ASHA), superstition, and not knowing that they were pregnant. Wealthier women who received antenatal care in private facilities would also be missed by the government health system or registered only after giving birth.

FLHWs place high value on collecting and digitizing beneficiary data

FLHWs in Rajasthan and Madhya Pradesh articulated a clear focus on accurately collecting and digitizing women's bank account details and identification numbers, since this data enabled women to receive financial support from government programs. While phone numbers were considered important for ASHA-beneficiary communication, the value of digitizing this information (rather than just ensuring it was collected in the ASHA's paper records) was not always clear. Some health workers in Madhya Pradesh could only articulate a vague sense that entering mobile numbers in RCH was useful. Most FLHWs in Madhya Pradesh reported that mobile numbers were used by the state or central government to call beneficiaries and check whether they are receiving appropriate health services, suggesting that FLHWs saw the digitization of women's mobile numbers as a means for the government to keep tabs on FLHW performance. In Rajasthan, FLHWs were clear about the value of mobile numbers in PCTS, and explained that the numbers were not only used for checking that women were receiving health services, but also for conveying health information, advertising government programs, and notifying women about financial transfers.

FLHWs describe several strategies for trying to collect accurate data

In Rajasthan and Madhya Pradesh, ASHAs and ANMs explained that many women could not accurately recall their mobile numbers at antenatal care visits. For cases when women did not have mobile phones with them, ASHAs would visit their homes and ask other family members to provide a mobile number or ASHAs and ANMs would ask women to bring a mobile number written on a piece of paper to the next antenatal care checkup.

> Like, they don't know, some of them.. they don't know the mobile number. So, we tell them to ask the people in her house who can write, to note it down on a piece of paper, and bring it to us. Tell them the sister [nurse] needs it, for your information. So they do that. (MP_IDI_FLHW_01, ANM)

For peer review only - http://bmjopen.bmj.com/site/about/guidelines.xhtml

ASHAs were confident that they received accurate numbers because they reported frequently calling women using the number provided. In cases when women had mobile phones with them, some ANMs or ASHAs reported giving themselves a missed call from beneficiary mobile phones to identify the number; however, the use of missed calls was not a universal practice, as observed during beneficiary – FLHW interactions.

FLHWs said that pregnant women sometimes asked FLHWs to record their husband's mobile number rather than their own, even when she had her own phone. Women in one FGD (RJN_FGD_BENEF_10) explained that the husband's mobile number was often provided so that he would receive the SMS notification of a financial transfer into the beneficiary's bank account. While some FLHWs complied in taking the husband's number, others tried to convince woman to provide her own mobile number since her husband will frequently be out of the house with the mobile.

FLHWs reported asking women to provide photocopies of bank account information and government identification cards, in order to reduce errors that could be introduced by women conveying this information on scraps of paper. When assessing last menstrual period in order to estimate gestational age, two FLHWs noted that many women struggled to report exact dates. FLHWs estimated last menstrual period based on women's recall of the moon cycle, harvest, or festivals, which would introduce inaccuracy around pregnancy stage.

Level 3. Creation of paper records

Beneficiary data copied by ASHAs and ANMs across multiple paper forms and registers

In Madhya Pradesh and Rajasthan, when an ASHA identified a pregnancy in the village she would write key information about the woman in her official ASHA diary. In cases where ASHAs were considered sufficiently literate by the local ANM, the ASHA was also expected to start a paper RCH form (in Madhya Pradesh) or ANC form (in Rajasthan) by filling in some initial fields. In Madhya Pradesh we found that ASHAs in one block reported being paid Rs. 50 ($0.80) for each form they created, while in a nearby block ASHAs received no payment. In both states, some ASHAs first wrote the pregnant woman's details in an unofficial notebook and then copied this information into the official ASHA diary and the RCH/ANC form. While some ASHAs were clear and confident about their role filling in the RCH/ANC form, others were unsure about which documents they completed.

When the woman comes the village health and nutrition day (an outreach event where antenatal care is provided by ANMs), the ASHA will pass the partially-filled RCH/ANC form to the ANM. The ANM will fill in additional details from the woman's first antenatal care visit, often with the ASHA's help. ANMs explained that they were overwhelmed by documentation requirements and thus had to take assistance from ASHAs, even when ASHAs struggled with the literacy and numeracy requirements of this work.

> We have to fill a big register, we make sehyogini [ASHA] fill it that is the reason it gets wrong. Now they [ASHAs] don't understand. They themselves have filled

For peer review only - http://bmjopen.bmj.com/site/about/guidelines.xhtml

> the card. Now, there was so much crowd that time, that I was writing something.. and if they say, the weight is 53 and she says its 61, so then there's confusion like that. So sometimes they write it wrong. When there's a crowd sometimes, we take help from them only, and it becomes wrong. (MP_IDI_FLHW_07, ANM)

In addition to the RCH/ANC form, ANMs record data about pregnant women in the ANM RCH register (a book) and create a Mother and Child Protection (MCP) card (Madhya Pradesh) or Mamta card (Rajasthan). MCP/Mamta card is given to the pregnant woman to keep. Similar to our observations of ASHAs, ANMs in Madhya Pradesh also sometimes wrote down beneficiary information in unofficial notebooks and then copied this information into their official RCH register and the RCH forms once they returned home; they explained that the RCH register is heavy and they wanted to keep it neat and clean. In Rajasthan the official register was smaller and more portable so ANMs tended to carry it with them.

Delays in antenatal care have implications for the creation of health records

There could be several weeks or even months between the ASHA becoming aware of a pregnancy and the ANM meeting the woman for first antenatal care. No paper record could be completed until the woman interacted with an ANM for antenatal care, thus introducing delay in the production of official health records.

FLHWs found data collection under RCH to be too time-intensive

ANMs in Madhya Pradesh said that they found data collection to be highly burdensome. They felt that their work had shifted from healthcare provision to finding documents and filling in forms. FLHWs described facing pressure to complete the digital records from senior actors in the health system hierarchy as well as from beneficiaries who were worried about delays in receiving their financial incentives. They described having to fill up to 70 data fields in various paper forms and registers, and having to track down beneficiary banking and identification details, without adequate time or support.

> We have to do this also and that also. In this what happens is, like this “Prashuti Sahayata Yojana” has come [i.e., yet another scheme has been introduced]. So, we keep on calling them the whole day that “Get this paper” “This is not enough” “This paper is short.” Just now also madam [a supervisor] called. And sir [another supervisor] also called saying that high risk pregnancy too needs to be checked too. (MP_IDI_FLHW_06, ANM)

> They've increased our work a lot, a lot. … We need to visit each beneficiary 10 times… “Give the Aadhar card, the Samagrah ID.” The beneficiary isn't able to submit, isn't able to give it to us, and above that they reach here and start complaining, that, madam isn't filling our form so we aren't getting our money when they themselves aren't giving us the documents all together. We used to go to each house, of the beneficiary, give them the vaccinations, meet each one. And the bai [women] that were there, they had very good behaviour, were respectful, and would make us sit inside, that come, madam, sit inside. Would treat us very well. And, these days I don't know what is happening, so the beneficiaries also snap at us, for a little bit. If they say, madam we need the money, and we tell them to submit the documents, and they'll get the money... Oh oh, but you need this paper, and that paper, and we've given it to the

For peer review only - http://bmjopen.bmj.com/site/about/guidelines.xhtml

> Anganwadi, to you, and to the ASHA, what is this nonsense. (MP_IDI_FLHW_07, ANM)

ANMs who worked with low-literacy ASHAs noted that they faced an additional struggle because they could not rely on the ASHAs to complete any paper forms.

Level 4. Paper records brought to health centre

Delay between creation of paper records and records reaching the health center with a computer

The ANM becomes aware of a pregnancy during the woman's first antenatal care visit and considers the woman's pregnancy to have been registered at this time. However, the pregnancy is not entered into the online portal until the ANM brings the RCH/ANC forms and RCH registers to the health center for digitization. This occurs only once per month. In addition, if an ANM is waiting for a beneficiary to provide documents required to complete the RCH/ANC form, she will hold on the form even longer, until a future visit to the health center.

> There are some women in the village, even after their delivery, their Samagra ID isn't able to be made..[…] [So] It's not fed [entered into RCH] here because the Samagra ID is incomplete. (MP_IDI_FLHW_01, ANM)

Level 5. Data entry

DEOs spoke of high workloads and some technical challenges but noted recent improvements and supportive peers

DEOs in both states said there was excess workload, which could lead to data entry backlog. They explained that in addition to entering maternal and child health data they were also responsible for entering data from the pharmacy, ASHA records, birth and death registration, HMIS, and health facility forms. In some areas, one DEO was covering the workload of two or three because of vacancies. ANMs suggested that DEOs may make data entry errors, because they were rushing or careless.

In both states the DEOs mentioned that the infrastructure to support them was improving, such as the provision of newer computers, faster internet, and upgraded servers. In Rajasthan the DEOs had battery backup for power outages and a dedicated broadband internet connection; even so, during observation, opening the PCTS portal took up to 10 minutes because of slow internet connection or server capacity. In Madhya Pradesh, many DEOs did not have functioning battery backup, leaving them unable to work when the electricity went off. Some relied on their own portable WIFI device or their mobile phone's hotspot, both of which were often slow.

For peer review only - http://bmjopen.bmj.com/site/about/guidelines.xhtml

DEOs in Madhya Pradesh reported having received only one training on RCH, while DEOs in Rajasthan received frequent trainings and felt reasonably well supported by the supervisory structure as well as their peers. The one-time training in Madhya Pradesh occurred years ago, and many months before RCH was introduced so recipients forgot much of it. It consisted of a presentation without any hands-on learning. DEOs reported learning how to use RCH on the job, through trial and error and from other DEOs, who provided ongoing peer support.

Required fields and auto-fill invite errors

The digital portals had numerous required fields, validation checks, and autofill features, including that 10 digits be entered in the mobile number field. In many cases these features supported accurate and complete data entry. However, DEOs recalled instances where these features forced them to introduce errors into the system. For instance, when DEOs encountered forms that did not have a beneficiary mobile number, DEOs reported using the ASHA's number or their own number because the mobile number field is mandatory. In Madhya Pradesh, DEOs reported that RCH initially required all mobile numbers to start with the digit 9. When a new phone company began issuing mobile numbers beginning with 6 or 7, DEOs could not get the system to accept these numbers until the RCH portal was updated.

In RCH, pregnancy records are linked to eligible couple record. The pre-existing eligible couple registry auto-completes fields in the new pregnancy registry, including for the mobile number. Thus the phone number provided by a family at time of marriage becomes the default current phone number at the time of pregnancy.

DEO and ANM absences can lead to data entry delays and may compromise quality

When DEOs took time off, there were not always additional trained personnel to fill in. Other health system actors (such as a PHC health supervisor or ASHA supervisor) would take over data entry, even without training. In cases of longer-term absence, such as when there was no DEO appointed to a PHC at all, a DEO at a neighbouring facility would be assigned to cover both catchment areas. ANMs then had to bring their paper records to a more distant health facility to have them digitized and DEOs had to cover double the caseload.

There are gaps in data collection at the frontlines when ANMs take time off, during transition from an older ANM to a new recruit, or when the ANM position is vacant. During these periods no health records are created or digitized but the district health system actors demand that data continues to be entered. This leaves the DEO in a difficult situation wherein no paper records are being passed on to them but they are expected to create digital records.

Level 6. Ongoing use of paper and online records

For peer review only - http://bmjopen.bmj.com/site/about/guidelines.xhtml

For peer review only

Mobile numbers are not corrected or updated in the online system

Beneficiaries explained that they updated their ASHA whenever they changed mobile numbers and ASHAs were confident that they retained up to date mobile numbers for the pregnant women in their villages. However, ASHAs, ANMs and DEOs reported that they do not update or change mobile numbers in the online portals. Some DEOs were unsure if it is even possible to do so. Checking and correcting bank account details and government identification details was common, since this ensured financial transfers proceeded. Ensuring correct and up-to-date mobile numbers was not a priority. Even among women and FLHWs who identified benefits for women who shared their mobile number with the government, the value of receiving government information and notifications was overshadowed by the importance of receiving financial incentives.

Supervision focuses on completeness not accuracy

DEOs as well as block, district and state level actors focused on data entry completion and timeliness but not on accuracy. DEOs were encouraged to complete their data entry activities each month and clear backlog. However attention to data accuracy only arose when mistakes came to light through irregular means, such as a financial transfer going into the wrong bank account or a polio outbreak alert being triggered by a DEO incorrectly entering eight polio cases rather than zero.

In Madhya Pradesh, some health facilities had multi-purpose supervisors and ASHA support staff who checked the forms and registers provided by ANMs and ASHAs to the DEO and who oversaw the DEOs work. These supervisors could flag potential inaccuracies for follow up. However in other facilities these positions were vacant. The Medical Officer was expected to sign off on the DEO’s work but was so busy with patient care that they often gave an automatic approval or even provided their login and password to the DEO so that the DEO could approve their own work.

In both Madhya Pradesh and Rajasthan, DEO work at the health centers was overseen by block level supervisors. In both states these actors reported being overwhelmed by having to oversee five to seven software programs and more than 50 reporting processes and could only focus on issues of incomplete data.

At the frontlines, ANMs and ASHAs in Madhya Pradesh and RJN had no way of knowing whether the mobile number entered in PCTS or RCH was correct and had never been asked to cross check these digital records with beneficiary numbers.

Health system actors have an overall positive view of the value of digitization

Despite noting hiccups and increased documentation burden, ANMs and ASHAs were generally positive about the move towards digital health records. They framed the use of digital systems as a better way for them to showcase their hard work and to receive recognition (and, for ASHAs, performance based remuneration), rather than a way to benefit from the digital system’s ability to generate ANM workplans or other synthesized data. Block, district and state level actors described benefiting enormously from the digital system. The explained that it enabled them to see the progress of programs, monitor

For peer review only - http://bmjopen.bmj.com/site/about/guidelines.xhtml

service provision by FLHWs, conduct yearly planning to address gaps, and prioritize resources: “In one click the full report can be retrieved” (RJN_IDI_MO_04, doctor).

Discussion

This study explored how beneficiaries, frontline health workers, data entry operators and higher level actors in the government health system experienced HIS digitization to understand barriers to accurate, complete, and timely capture of mobile phone numbers and other data (Table 4). We found that frontline workers and their supervisors prioritized the accuracy of administrative data elements that enabled pregnant clients to receive government financial entitlements (e.g., bank account information, identification numbers) over mobile phone numbers. Providers reported that it often took months – and significant effort that detracted from healthcare provision – to gather the documents and information from pregnant clients required to create a digital record; frontline workers relied on their existing paper systems to provide ongoing healthcare and keep track of up-to-date beneficiary mobile numbers.

For peer review only

Table 4. Summary of barriers to the creation of timely, accurate and complete mobile phone records in MCTS/RCH data

	Barriers to timely data	Barriers to accurate and complete data
1. Beneficiary	• Beneficiaries must open bank accounts and attain government identification before an electronic record can be created.	• Duplicate mobile phone entries are created when one number is provided by multiple pregnant women, such as when multiple women in a joint family with one phone become pregnant, or when multiple women provide a neighbour's number or the ASHA's number.<br>• Non-beneficiary numbers are entered when women without personal mobile phones provide their husband's number, a joint family phone number, a neighbour's number, or the ASHA's number.
2. Beneficiary-FLHW	• While ASHAs learn of most pregnancies within the first trimester, pregnancies among migrants, women living in remote communities, very poor women who did not see the value in seeking early antenatal care, and wealthy women who received antenatal care in the private sector were all detected late – sometimes even after the baby was born.	• FLHWs value collecting correct mobile numbers so that ASHAs can keep in touch with beneficiaries. The value of accurately digitizing these numbers is not always clear to FLHWs in Madhya Pradesh. In both Rajasthan and Madhya Pradesh accurate mobile phone number digitization does not have immediate or direct influence on health care or financial transfers.<br>• While ASHAs and ANMs have a number of strategies to seek correct mobile numbers, beneficiaries may incorrectly recall their mobile numbers; checking the beneficiary's number through a missed call is common but not universal and is not an official requirement.<br>• Accurate gestational age estimation (which is linked to the provision of stage-based information in Kilkari) is a challenge since women cannot always report the exact date of their last menstrual period.
3. FLHW creates paper records	• There can be a delay of weeks or even months between an ASHA learning of a new pregnancy and the woman receiving her first antenatal care from the ANM at village health and nutrition days.<br>• It is only after the woman meets the ANM that her (paper) antenatal care / RCH form can be filled in with details of the first antenatal care visit.	• Copying errors can occur when ASHAs and ANMs write women's information in multiple places for different programs, often with slightly different data fields; beneficiaries may also provide their mobile phone numbers on scraps of paper.<br>• ASHAs are often expected to complete many fields in the RCH/ANC form before the ANM fills in additional details and passes it to the DEO; ASHAs with lower literacy struggled with this responsibility.<br>• FLWHs in Madhya Pradesh found data collection for RCH to be highly burdensome and described high pressure from above to fill forms and registries without adequate time or support.<br>• Some ASHAs were confused about the names and purposes of various paper forms and some hand made their own additional registries that they use in the field.
4. FLHW brings paper records to health centre	• Many ANMs bring paper antenatal care / RCH forms and registers to the health center for data entry only once a month.<br>• A woman's paper form cannot be passed to the health center until she	• Respondents did not note any risks to data accuracy or completeness while transporting paper forms and registers from the field to the data digitization facility.

For peer review only - http://bmjopen.bmj.com/site/about/guidelines.xhtml

	Barriers to timely data	Barriers to accurate and complete data
	has provided all required fields (such as a bank account number), which may introduce further delays.	
5. Data entry into online portal	• Server errors, internet and electricity issues may delay DEO in creating electronic records.<br>• If DEOs are overburdened with data entry activities, they can fall behind.	• When entering a pregnancy into the online portal, RCH auto-fills the mobile number provided at the time of "eligible couple" registration; this number may be outdated.<br>• DEOs in Madhya Pradesh found their training on RCH to be insufficient; peer support enabled DEOs to navigate challenges. DEOs in Rajasthan were confident in PCTS and received frequent training.<br>• When DEOs are on leave or when DEO positions are vacant result in other health facility employees, who have not been trained, perform data entry.
6. Ongoing use of paper and online records	None identified	• Although the ASHA is generally notified if a pregnant woman's contact number changes over the course of her pregnancy or postpartum, this new number will not be updated in the PCTS/RHC portals.<br>• Supervisor positions remain vacant in some health facilities and higher level supervisors (block and district level) oversee numerous databases and reporting systems.<br>• Supervisors tend to focus on completeness and timeliness rather than accuracy.

For peer review only

Scholars have critiqued India's health system digitization as disproportionately focused on the collection of data rather than its use.[20,21] However the health system actors that we interviewed conveyed a clear understanding of ways in which the digital HIS supports work planning and monitoring. Nonetheless, the overarching logic behind digitization articulated by frontline workers remained as a mechanism to feed information up the health system hierarchy rather than to improve their work planning or to access information about their clients.

While pregnant women were willing to share their or their family's mobile numbers with ASHAs and ANMs, many were unaware that their mobile numbers were passed onward and entered into computers. Although women trusted the health workers and associated the provision of requested data with receiving benefits from the government, strengthening consent processes can protect beneficiaries and safeguard relationships at the frontlines. These processes can inform beneficiaries and frontline providers about the benefits of ensuring accurate mobile numbers are registered in the digital system (including that beneficiaries can be subscribed to mHealth programs such as Kilkari), potentially increasing both parties' motivation to keep the registry up-to-date.

As identified earlier by other researchers,[22] we found poor standardization across multiple data systems (paper based and digital) and identified opportunities for streamlining records and entry options to become more intuitive and user friendly. Echoing findings from other settings,[23] supervision focused on completeness rather than accuracy, highlighted by the fact that there were no mechanisms to check whether mobile numbers were correct. Workers at the frontlines require clear guidelines on how to handle common challenges in accessing data, particularly mobile phone numbers, including how to handle data changes (such as new phone numbers) and unavailable data (such as no phone number). Health workers require training on the value of maintaining an up-to-date mobile number in their data registries, so that they are motivated to ensure these numbers are correct. Furthermore, in light of research findings that "SIM churn" (changing SIM cards, and as a result getting a new mobile number) is as high as 44% per year among rural families,[10] frontline workers need to regularly confirm beneficiary mobile numbers and have access to user-friendly mechanisms to update numbers whenever they change.

Required fields, auto-fill features and validation checks can force DEOs to adjust the data provided to them according to their judgement in order to proceed with data entry. Another study on PCTS in Rajasthan found that the portal would not accept delivery dates that appeared to occur after more than 9 months gestation, a situation created by incorrect gestational age estimation based on faulty last menstrual period estimates.[24] Finally, the consolidation and standardization of multiple records may reduce errors introduced by manual copying from form to form and reduce the work burden on frontline actors.

Three core determinants of accurate, complete and timely administrative data emerged from our research and are likely applicable to health registries for pregnant and postpartum women in other lower resource settings. First, beneficiaries who trust their health workers and the government more broadly are willing to provide accurate data.[25,26] Second, data entry systems that are easy to use, appropriate to the local context, and useful to the

For peer review only - http://bmjopen.bmj.com/site/about/guidelines.xhtml

frontline worker facilitate data accuracy and timeliness.[8,27] And third, supportive supervision and adequate resources are vital to building frontline health worker skills and knowledge across clinical and administrative functions.[28–30]

This study has some limitations, which point towards areas for future research. The information presented here was either gathered through interviews and focus groups and thus self-reported, or was gathered through observation, wherein data entry operators were aware that their work was being observed. Thus respondents may have minimized or hidden unauthorized behavior, such as shortcuts to speed up data entry or unsanctioned task-shifting. Future quantitative research could more objectively measure data delays (e.g., time elapsed from first antenatal interaction with an actor from the government health system to the creation of a digital record) and data accuracy (e.g., systematically validating key data fields, including mobile phone numbers, for a sample of records). For this study, we spoke to pregnant and post-partum women with mobile phones who recently interacted with health system actors: this sample enabled us to understand reasons for delays and inaccuracies among beneficiaries whose data should have been easy to digitize. Future research among women without mobile phones and women who interacted with the government health system many months after childbirth would triangulate understanding about the data sharing practices of these additional populations. Furthermore, research with additional beneficiaries could provide richer insight into equity dimensions related to different caste, religion, socioeconomic status or educational levels. Such insights would best be gleaned through targeted in-depth interviews with specific marginalized populations and FGDs that separated women by caste, religion, socioeconomic status or educational levels.

Conclusion

Frontline health workers are broadly supportive of the move towards digitization despite implementation challenge. Beneficiary willingness to share their information with health workers – including their mobile phone numbers – highlights their trust in health workers and the value they place on government financial incentives. This positive engagement bodes well for derivative uses of HIS, such as for mHealth programs and surveys. However the underlying causes of inaccuracies and severe delays must be addressed before the true benefit of these uses will be realised. In addition to data system and supervisory supports, frontline workers and beneficiaries themselves must understand and experience the benefits of accurate, complete and timely digital health information systems.

Acknowledgements

The authors are grateful to the health system actors who generously provided their time and insights to make this research possible. This work was made possible by the Bill and Melinda Gates Foundation. We thank Diva Dhar, Suhel Bidani, Rahul Mullick, Dr. Suneeta Krishnan, Dr. Neeta Goel and Dr. Priya Nanda for giving us this opportunity. We additionally thank Vinit Pattnaik at OPM and Erica Crawford at Johns Hopkins for their support to the financial management of our work.

For peer review only - http://bmjopen.bmj.com/site/about/guidelines.xhtml

Competing interests

All authors have completed the Unified Competing Interest form (available on request from the corresponding author) and declare that the research reported was funded by the Bill and Melinda Gates Foundation. The authors do not have any relationships or engagement in activities that could appear to have influenced the submitted work.

Data sharing

Data for this study consist of qualitative interview and focus group discussion transcripts. Uploading all transcripts for open availability would compromise our ability to fully mask participant details. However, we are happy to share anonymized portions of these transcripts upon reasonable request.

Contributor statement

KS, SC , OU, DM and AEL conceptualized and designed the study. MS, DG, BM and NC conducted the data collection and preliminary data analysis through daily analytic debriefs. KS and OU led and managed the data collection and analysis, including the coding and thematic synthesis. KS drafted the manuscript and revised it based on critical and substantive input from SC, OU, AEL, MS, BM, DM and NC. All authors agree to be accountable for all aspects of the work related to accuracy and integrity.

Funding statement

This work was supported by the Bill and Melinda Gates Foundation grant number OPP1179252.

Works Cited

1. WHO. Framework and Standards for Country Health Information Systems, 2nd Edition. Geneva, Switzerland; 2012.
2. WHO. Health Metrics Network Framework and Standards for Country Health Information Systems. 2008.
3. Al-Shorbaji N, Geissbuhler A. Establishing an evidence base for e-health: the proof is in the pudding. Bull World Health Organ. 2012;90:322.
4. WHO. Resolution WHA58.28. eHealth. WHA58/2005/REC/1 Fifty-eighth World Health Assembly, Geneva, 16–25 May 2005. Annex. Resolutions and decisions. Geneva; 2005.
5. Lewis T, Synowiec C, Lagomarsino G, Schweitzer J. E-health in low- and middle-income countries: findings from the Center for Health Market Innovations. Bull World Heal Organ. 2012;90:332–40.

For peer review only - http://bmjopen.bmj.com/site/about/guidelines.xhtml

6. Neupane S, Odendaal W, Friedman I, Jassat W, Schneider H, Doherty T. Comparing a paper based monitoring and evaluation system to a mHealth system to support the national community health worker programme, South Africa: an evaluation. BMC Med Inform Decis Mak. 2014 Dec 9;14(1):69.
7. Tamrat T, Kachnowski S. Special Delivery: An Analysis of mHealth in Maternal and Newborn Health Programs and Their Outcomes Around the World. Matern Child Health J. 2012 Jul 19;16(5):1092–101.
8. Charanthimath U, Katageri G, Kinshella M-LW, Mallapur A, Goudar S, Ramadurg U, et al. Community Health Worker Evaluation of Implementing an mHealth Application to Support Maternal Health Care in Rural India. Front Glob Women's Heal 2021 Sep 1;2.
9. Källander K, Tibenderana JK, Akpogheneta OJ, Strachan DL, Hill Z, Asbroek A, et al. Mobile health (mhealth) approaches and lessons for increased performance and retention of community health workers in lowand middle-income countries: A review. J Med Internet Res. 2013;15(1).
10. Chamberlain S, Dutt P, Godfrey A, Mitra R, LeFevre AE, Scott K, et al. Ten lessons learnt: scaling and transitioning one of the largest mobile health communication programmes in the world to a national government. BMJ Glob Heal. 2021 Jul 26;6(Suppl 5):e005341.
11. Scott K, Ummer O, Shinde A, Sharma M, Yadav S, Jairath A, et al. Another voice in the crowd: The challenge of changing family planning and child feeding practices through mHealth messaging in rural central India. BMJ Glob Heal. 2021;6.
12. Bashingwa JJH, Mohan D, Chamberlain S, Arora S, Mendiratta J, Rahul S, et al. Assessing exposure to Kilkari: A big data analysis of a large maternal mobile messaging service across 13 states in India. BMJ Glob Heal. 2021;6.
13. Gera R, Muthusamy N, Bahulekar A, Sharma A, Singh P, Sekhar A. An in-depth assessment of India's Mother and Child Tracking System (MCTS) in Rajasthan and Uttar Pradesh. BMC Health Serv Res. 2015;1–14.
14. Balakrishnan R, Gopichandran V, Chaturvedi S, Chatterjee R, Mahapatra T, Chaudhuri I. Continuum of Care Services for Maternal and Child Health using mobile technology – a health system strengthening strategy in low and middle income countries. BMC Med Inform Decis Mak. 2016;16(84):1–8.
15. SRS. Special Bulletin on Maternal Mortality In India 2016-18. New Delhi; 2020.
16. MoHFW India. National Family Health Survey 4 (2015-2016): State Fact Sheet Rajasthan. Mumbai; 2017.
17. MoHFW India. National Family Health Survey - 4 (2015-16); State Fact Sheet Madhya Pradesh. 2017.
18. GoI. RCH Application and ANMOL: Data Elements, Validation, Logic and Tool Tip Document, V 2.0. 2016.
19. Attride-Stirling J. Thematic Networks: an analytic tool for qualitative research. Qual Res. 2001;1(3):385–405.
20. Mukherjee A. Who's data is this anyway? Understanding relationship between power, empowerment and technology: Case from Mother and Child Tracking System (MCTS) in India. Proceedings of the 9 th Health Informatics in Africa Conference HELINA'14 PART 2. Hamburg, Germany; 2014.
21. NHSRC and Taurus Glocal Consult. Study of Public Health IT Systems in India. New Delhi, India; 2011.
22. Gera R, Muthusamy N, Bahulekar A, Sharma A, Singh P, Sekhar A. An in-depth

For peer review only

assessment of India's Mother and Child Tracking System (MCTS) in Rajasthan and Uttar Pradesh. BMC Health Serv Res. 2015;15:315.
23. Mphatswe W, Mate K, Bennett B, Ngidi H, Reddy J, Barker P, et al. Improving public health information: a data quality intervention in KwaZulu-Natal, South Africa. Bull World Health Organ. 2012 Mar 1;90(3):176–82.
24. The Earth Institute. Pregnancy Child Tracking and Health Services Management System (PCTS) Gap Analysis -- Rajasthan. Mumbai; 2014.
25. Levy AG, Scherer AM, Zikmund-Fisher BJ, Larkin K, Barnes GD, Fagerlin A. Prevalence of and Factors Associated With Patient Nondisclosure of Medically Relevant Information to Clinicians. JAMA Netw Open. 2018 Nov 30;1(7):e185293.
26. Agaku IT, Adisa AO, Ayo-Yusuf OA, Connolly GN. Concern about security and privacy, and perceived control over collection and use of health information are related to withholding of health information from healthcare providers. J Am Med Informatics Assoc. 2014 Mar 1;21(2):374–8.
27. Dickinson FM, McCauley M, Madaj B, Van Den Broek N. Using electronic tablets for data collection for healthcare service and maternal health assessments in low resource settings: Lessons learnt. BMC Health Serv Res. 2019;19(1).
28. Gopalakrishnan L, Diamond-Smith N, Avula R, Menon P, Fernald L, Walker D, et al. Association between supportive supervision and performance of community health workers in India: a longitudinal multi-level analysis. Hum Resour Health. 2021 Dec 27;19(1):145.
29. Sadiq Umar A, Mohammed Bello I, Chukwudi Okeibunor J, Mkanda P, Ubong Akpan G, Manyanya D, et al. The Effect of Real Time Integrated Supportive Supervision Visits on the Performance of Health Workers in Zambia. J Immunol Sci. 2021;Special Issue(2):120–6.
30. WHO. Training for mid-level managers (MLM). Module 4. Supportive Supervision. Geneva, Switzerland; 2008.

For peer review only

For peer review only - http://bmjopen.bmj.com/site/about/guidelines.xhtml

For peer review only

For peer review only - http://bmjopen.bmj.com/site/about/guidelines.xhtml

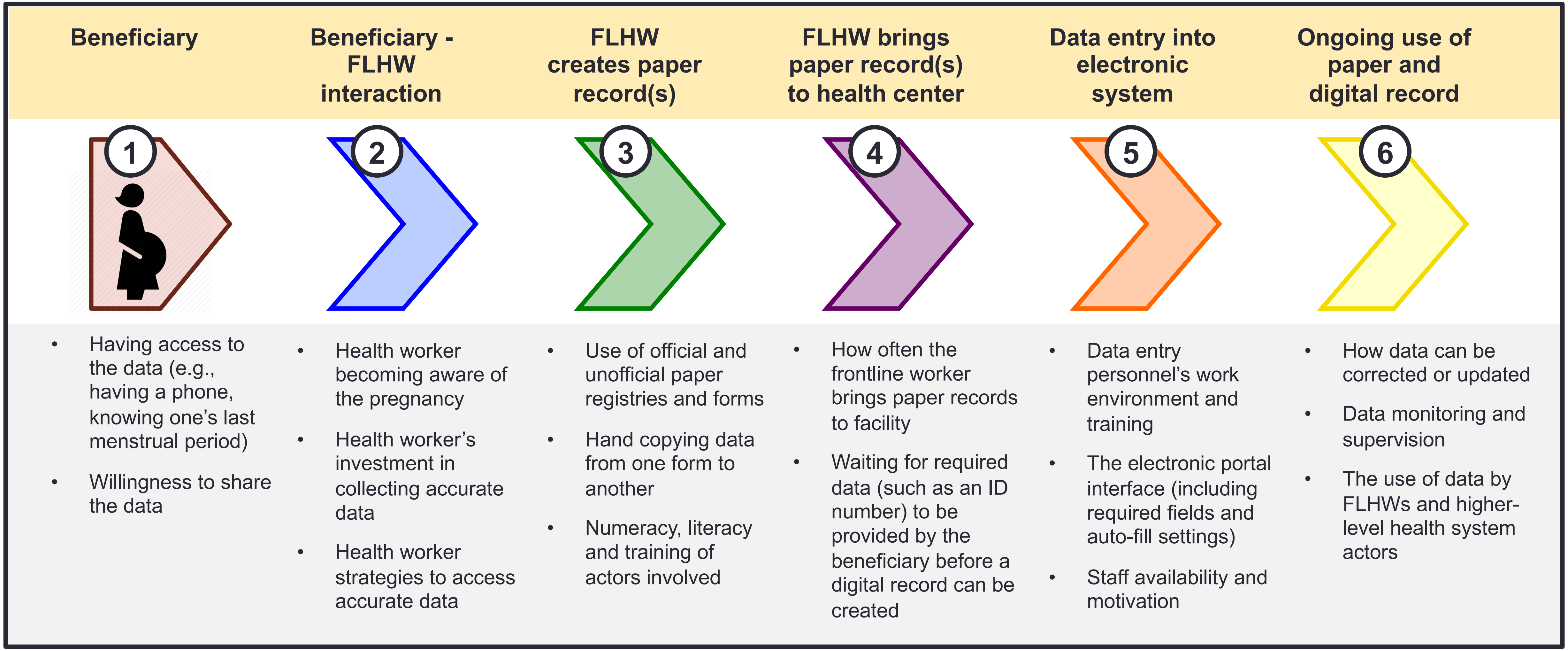
Beneficiary
Beneficiary - FLHW interaction
FLHW creates paper record(s)
FLHW brings paper record(s) to health center
Data entry into electronic system
Ongoing use of paper and digital record
1
2
3
4
5
6
• Having access to the data (e.g., having a phone, knowing one's last menstrual period)
• Willingness to share the data
• Health worker becoming aware of the pregnancy
• Health worker's investment in collecting accurate data
• Health worker strategies to access accurate data
• Use of official and unofficial paper registries and forms
• Hand copying data from one form to another
• Numeracy, literacy and training of actors involved
• How often the frontline worker brings paper records to facility
• Waiting for required data (such as an ID number) to be provided by the beneficiary before a digital record can be created
• Data entry personnel's work environment and training
• The electronic portal interface (including required fields and auto-fill settings)
• Staff availability and motivation
• How data can be corrected or updated
• Data monitoring and supervision
• The use of data by FLHWs and higher-level health system actors

Annex 1. Acronyms and key terms used

ASHA	Accredited Social Health Activist (ASHA Sahyogini in Rajasthan), a female community health worker
AWW or Anganwadi	Anganwadi Worker, a female community nutrition and preschool functionary
ANM	Auxiliary nurse midwife, a female frontline health worker who provides outreach and some clinical maternal and child health care
DEO	Data entry operator
FGD	Focus group discussion
FLHW	Frontline health workers
HIS	Health information systems
ICT	Information communication technologies
IDI	In-depth interview
MCP card	Mother Child Protection card, a health tracking and information card filled out by the frontline worker and kept by the pregnant woman
MCTS	Maternal and Child Health Tracking System / Reproductive and Child Health system. MCTS is an initiative of Ministry of Health & Family Welfare to leverage information technology for ensuring delivery of full spectrum of healthcare and immunization services to pregnant women and children up to 5 years of age. It facilitates and monitors service delivery and also establishes a two way communication between the service providers and beneficiaries (Ref: Digital India, MoHFW, http://nrhm-mcts.nic.in/Home.aspx [accessed 3 Dec 2021])
mHealth	Mobile health, use of mobile and wireless technologies to support the achievement of health objectives
MO	Medical Officer (a doctor)
PCTS	Pregnancy, Child Tracking, and Health Services Management System (Rajasthan's version of MCTS, which also syncs with MCTS)
RCH portal	Reproductive and Child Health portal. RCH (an upgraded version of MCTS) has been designed for early identification and tracking of the individual beneficiary throughout the reproductive lifecycle. While MCTS tracks every pregnancy, RCH portal tracks a woman throughout her reproductive life cycle, thus making available information on obstetric history. (Ref: RCH Portal & Allied Initiatives of MoHFW, 2018, https://icds-wcd.nic.in/nnm/Events/TechThon/ExistingTechnologyPlatform_for_HealthSchemes_MoHFW-28-06-2018.pdf [accessed 3 Dec 2021])

For peer review only

At the frontlines of digitization: a qualitative study on the challenges and opportunities in maintaining accurate, complete, and timely digital health records in India's government health system

Supplementary file 1: Sample IDI guide and FGD guide

INTERVIEW GUIDE WITH FRONTLINE PROVIDERS
साक्षात्कार निर्देशिका

Respondent criteria:
जिनका साक्षात्कार लेना है, उनके मानदंड:

- Frontline provider (ANM, ASHA, AWW, nurse, medical officer)
  अगुआ प्रदाता (एएनएम, आशा, एडब्लूडब्लू, नर्स, मेडिकल ऑफिसर)
- Collects MCTS/RCH data from pregnant or newly delivered women
  गर्भवती और नयी प्रसूति महिलाओं से एमसीटीएस/आरसीएच डेटा जमा करें
- Has been in the position for over five years
  अगुआ प्रदाता को कम से कम पांच साल का अनुभव हो

Preamble
प्रस्तावना
Thank you for agreeing to this interview. We're interested in understanding more about the maternal child health tracking system / reproductive child health tracking system, particularly the completeness of mobile phone registration. Please feel free to speak openly about how you feel, in terms of both good things and areas for improvement. There are no right or wrong answers.

इस बातचीत के लिए आप तैयार हुए/हुयीं, इसके लिए आपको धन्यवाद. मैटरनल चाइल्ड हेल्थ ट्रैकिंग सिस्टम/ रिप्रोडक्टिव चाइल्ड हेल्थ ट्रैकिंग सिस्टम को हम ज्यादा से ज्यादा समझने के इच्छुक हैं, विशेषकर मोबाइल फ़ोन पंजीकरण की पूर्णता (कम्पलीटनेस) के बारे में. इस संबंध में आपकी राय क्या है, इसे खुले तरीके से रखने के लिए आप स्वतंत्र हैं. हम दोनों तरह की जानकारियों की आपसे उम्मीद रखती/रखते हैं, वे चीजें जो अच्छी हैं और वैसे हिस्से भी जहाँ सुधार की गुंजाइश है. कोई भी उत्तर सही या गलत नहीं होता है.

1. **Introduction**
   परिचय

Before we discuss the main topics, I would like to learn a bit about you.
मुख्य विषय पर आने से पहले, मैं आपके बारे में कुछ जानना चाहती/चाहता हूँ.

- Could you tell me about yourself – where you're from and how you have come to this position?
  क्या आप अपने बारे में मुझे कुछ बता सकते/सकती हैं? जैसे, आप कहाँ से हैं और इस पद तक पहुँचने की यात्रा कैसे संभव हुयी?

2. **Late ANC registration**
   प्रसवपूर्व जांच के लिए देर से पंजीकरण

For peer review only

One issue we're interested in is late ANC registration.

लेट एएनसी रजिस्ट्रेशन (प्रसवपूर्व जांच के लिए देर से पंजीकरण) एक ऐसा मुद्दा है, जिसके बारे में हम जानने को इच्छुक हैं.

- When do women here typically register their pregnancies?
  यहाँ की महिलाएं अपने गर्भवस्था को आमतौर पर कब पंजीकृत करवाती हैं?
- Could you tell me about women's ANC options in terms of private or public care?
  क्या आप महिलाओं के एएनसी के विकल्पों, जैसे निजी या सरकारी, के बारे में मुझे बता सकते/सकती हैं?
- Which government health care providers might women interact with for their pregnancy registration?
  वे कौन-सी सरकारी स्वास्थ्य सेवा संस्थाएं हो सकती हैं, जहाँ महिलाएं अपने गर्भधारण के पंजीकरण के लिए संपर्क कर सकती हैं.
- What are some reasons for late ANC presentation?
  लेट एएनसी प्रेजेंटेशन के लिए कौन-से कारण जिम्मेवार हैं?

3. **Asking women from their mobile phone numbers**
   महिलाओं से उनके मोबाइल फोन नंबर पूछना

During ANC or the registration of a newborn, you often collect the woman's mobile phone number.

एएनसी या नवजात शिशु के पंजीकरण के दौरान, कर्मचारी महिला का मोबाइल फोन नंबर लेते हैं.

- Could you tell me about how this data is collected?
  ये आंकड़े कैसे जमा किये जाते हैं, क्या इसके बारे में आप मुझे बता सकते हैं?
- What questions do you ask women? How many times do you ask them?
  आप किस तरह के सवाल महिलायों से पूछते हैं? कितनी बार पूछती हैं?
- What are some challenges you have faced in registering a mobile number for each pregnant woman?
  हर गर्भवती महिला का मोबाइल फोन नंबर पंजीकृत करने में आपको कौन-सी चुनौतियों का सामना करना पड़ता है.

[If the respondent says that many women do not have mobiles, probe for other reasons]

जिनसे आप साक्षात्कार कर रहे हैं और वे कहते/कहती हैं कि बहुत सारी महिलायों के पास मोबाइल फ़ोन नहीं है, तब अन्य कारणों की जांच करें

- Among women who do have access to a mobile phone, what are some reasons they may not register it?

वैसी महिलाएं जिनके पास मोबाइल फ़ोन है, फिर भी शायद वे अपने मोबाइल फोन नंबर को पंजीकृत नहीं करवाती हैं, इसके क्या कारण हो सकते हैं?

- What about women not knowing their mobile number? How do health workers manage this?
  कुछ महिलायें ऐसी भी हैं जिनके पास मोबाइल फ़ोन नंबर तो है लेकिन उन्हें नंबर याद नहीं रहता, ऐसी स्थिति में स्वास्थ्यकर्मी इसे कैसे मैनेज करते/करती हैं?
- What about women not wanting to give their mobile numbers? What could be some reasons for this? How do health workers manage it?
  वैसी महिलाएं जो अपना मोबाइल फ़ोन नंबर देना नहीं चाहती हैं, इसके क्या कारण हो सकते हैं? ऐसी स्थिति में स्वास्थ्यकर्मी इसे कैसे मैनेज करते/करती हैं?

- Which women might be resistant or unable to register their mobile number?

  वे कौन-सी महिलाएं हैं जिन्हें अपने मोबाइल नंबर को पंजीकृत करने बाधाएं आती हैं या पंजीकृत करवा पाने में अक्षम हैं?

4. Gender considerations
लिंग आधारित सोच

In a family, men and women often have different access to and use of mobile phones.
एक परिवार में स्त्री और पुरुष के मोबाइल रखने और उसे इस्तेमाल करने के मामले में भेद-भाव अक्सर ही दीखता है.

- Could you tell me about any differences you have noticed between pregnant women versus the men in their families in terms of access to mobile phones?
  मोबाइल फ़ोन इस्तेमाल करने के मामले में परिवार के भीतर गर्भवती महिला और पुरुष के बीच किसी तरह के अंतर को आपने किया है?
- How about in terms of knowledge of and comfort with using mobile phones – how is this different between pregnant women versus men in their families?
  परिवार के भीतर गर्भवती महिला और पुरुष के बीच मोबाइल फोन इस्तेमाल करने की सहजता और जानकारी अलग-अलग कैसे है?
- Which families or communities might have more differences between male and female access to phones? Which families might have less of a difference? Why?

For peer review only

मोबाइल फ़ोन इस्तेमाल करने के मामले में स्त्री-पुरुष के बीच सबसे ज्यादा अंतर किन परिवारों और समुदायों में है? किन परिवारों/समुदायों में यह अंतर कम है? ऐसा क्यों?

- When seeking mobile phone numbers in order to register women in MCTS/RCH, in what ways do you involve male family members? [E.g. the ANM might send the woman to ask her husband for the mobile number in order to use it]
  एमसीटीएस/आरसीएच में महिलायों के पंजीकरण के दौरान मोबाइल फ़ोन नंबर जानने के लिए स्वास्थ्य कर्मचारी परिवार के पुरुष सदस्यों को किस तरह शामिल (इन्वॉल्व) करते हैं? [जैसे, एएनएम किसी महिला को अपने पति के पास भेज यह पूछने के लिए भेज सकती है कि मोबाइल फ़ोन नंबर क्या है, जिसका इस्तेमाल किया जा सके]

5. Data flow
आंकड़ों की यात्रा

Could you walk me through the process from when a woman gives her mobile number until it is registered in the electronic record system?
जब कोई महिला अपना मोबाइल नंबर देती है, और वह नंबर इलेक्ट्रॉनिक रिकॉर्ड सिस्टम में पंजीकृत होता है, तब तक की पूरी प्रक्रिया पर क्या आप कुछ रौशनी डाल सकते/सकती हैं?
Probe on each step:
प्रत्येक चरण की जांच करें:

- E.g. So first you write the number down.
  जैसे, सबसे पहले जब एएनएम लिखा जाता है.
  - Where is it written? Is it written in more than one place?
    यह कहाँ लिखा जाता है?
  - How do you feel about this process?
    इस प्रक्रिया के बारे में आप क्या सोचते/सोचती हैं?
  - What are some things that may go wrong at this stage?
    वे कौन-सी चीजें हैं जो शायद इस चरण में गलत हो सकती हैं?
  - How could this process be improved?
    इस प्रक्रिया में सुधार कैसे लाया जा सकता है?

- Then what do you do with the record?
  तब एएनएम रिकॉर्ड के साथ क्या करता है?
  - How do you feel about this process?
    इस प्रक्रिया के बारे में आप क्या सोचते/सोचती हैं?
  - What are some things that may go wrong at this stage?
    वैसी कौन-सी चीजें हैं जो इस चरण में गलत हो सकती हैं?
  - How could this process be improved?

For peer review only

इस प्रक्रिया में सुधार कैसे लाया जा सकता है?

- Then how is the record is entered into the electronic system?
  रिकॉर्ड को इलेक्ट्रॉनिक सिस्टम में फीड कैसे किया जाता है?
  - What is the procedure?
    इसकी प्रक्रिया क्या है?
  - Who is involved?
    इस प्रक्रिया में कौन लोग शामिल होते/होती हैं?
  - How do you feel about this process?
    इस प्रक्रिया के बारे में आप क्या सोचते/सोचती हैं?
  - What are some things that may go wrong at this stage?
    वे कौन-सी चीजें हैं जो इस चरण में गलत हो सकती हैं?
  - How could this process be improved?
    इस प्रक्रिया में सुधार कैसे लाया जा सकता है?

6. How MCTS/RCH has changed the work environment
एमसीटीएस/आरसीएच ने काम के माहौल को किस तरह/कैसे परिवर्तित किया है

MCTS/RCH are new systems and there are often challenges associated with introducing new systems.
एमसीटीएस/आरसीएच नयी प्रणाली है और नयी प्रणालियों के सामने आने से जुडी चुनौतियां भी वहां होती हैं.

- Can you tell me about how MCTS/RCH has changed pregnancy and birth documentation and registration?
  क्या आप मुझे बता सकते हैं कि एमसीटीएस/आरसीएच ने गर्भधारण और जन्म से सम्बंधित दस्तावेजीकरण और पंजीकरण को कैसे परिवर्तित कर दिया है?
- How has MCTS/RCH changed your work?
  मसीटीएस/आरसीएच ने आप के कामकाज को कैसे परिवर्तित किया है?
- How has MCTS/RCH changed the work done my others in the health system?
  स्वास्थ्य विभाग के अन्दर कार्यरत अन्य बहुत सारे लोगों के कामकाज को एमसीटीएस/आरसीएच को कैसे परिवर्तित किया है?
- How were things before?
  इसके पहले चीजें कैसी थीं?
- How do you feel about these new systems?
  इस नयी व्यवस्था-प्रणाली के बारे में आप क्या सोचते/सोचती हैं?

7. Training and supervision for data quality
प्रशिक्षण और आंकड़े की गुणवत्ता की देख-रेख

- What kind of training or information have you received about MCTS/RCH?

For peer review only - http://bmjopen.bmj.com/site/about/guidelines.xhtml

एमसीटीएस/आरसीएच संबंधी किस तरह की सूचना और प्रशिक्षण आपने पायी?

- How do you feel about the training and information given to you and others? What could make it better?
  एमसीटीएस/आरसीएच संबंधी किस तरह की सूचना और प्रशिक्षण अन्य लोगों ने पायी? इसे और बेहतर कैसे बनाया जा सकता है?
- What are some ways that the accuracy and completeness of data in MCTS/RCH, including mobile phone numbers, can be assured?
  वे कौन से तरीके हैं जिससे मोबाइल नंबर सहित एमसीटीएस /आरसीएच के डेटा को सटीक और पूर्ण रूप से बताया जा सकता है.
  - How do you feel about the quality assurance processes?
    गुणवत्ता जांचने वाली प्रक्रियाओं के बारे में आप की क्या राय है?
  - In an ideal world, what would happen to ensure accurate and complete data is registered in MCTS/RCH?
    एक आदर्श वातावरण में, एमसीटीएस/आरसीएच में सटीक और पूर्ण डेटा पंजीकृत करने के लिए क्या-क्या करना होगा?

8. Use of mobile phone number
मोबाइल फ़ोन नंबर का इस्तेमाल

So now I'd like to ask you about the benefit of MCTS/RCH.
अब मैं आपसे एमसीटीएस/आरसीएच के फायदे के बारे में पूछना चाहती/चाहता हूँ.

- What are some reasons why the government wants to register women's phone numbers?
  वे कौन-से कारण हैं कि सरकार महिलायों के फ़ोन नंबर को पंजीकृत करना चाहती है?
- What are some benefits for you if the woman's phone number is registered?
  यदि महिलायों के फ़ोन नंबर पंजीकृत होते हैं तो इसमें आपके लिए फायदेमंद क्या है?
- What are some benefits for the woman?
  पंजीकृत होने वाली महिलायों के लिए कौन-से फायदे हैं?

9. Corrections and updates to registered mobile numbers
पंजीकृत मोबाइल नंबरों को संशोधित कर उसे अपडेट करना/करवाना

Women may change their numbers, may acquire a phone, or may find out that their mobile number has been entered incorrectly in the system.
महिलाएं शायद अपना नंबर बदल ली हों, शायद एक मोबाइल ली हो या उसे पता चला हो कि सिस्टम में जो नंबर उसके नाम के साथ दर्ज है, वह सही नहीं है.

For peer review only

- What is the procedure for updating or correcting records? Probe on each step and what the challenges might be.
  रिकॉर्ड को अपडेट करने या सही करने की प्रक्रिया क्या है? हर चरण की जांच करें कि और क्या परेशानियां हो सकती हैं.
- How do you feel about this process?
  आप इस प्रक्रिया के बारे में क्या सोचते हैं?
- What are some reasons women may not correct or update the record?
  वे कौन-से कारण हैं शायद जिसके चलते महिलाएं रिकॉर्ड को सही या अपडेट नहीं करती हैं?
- What could make the process easier for women?
  वे कौन-से उपाय हैं जो महिलायों के लिए इस प्रक्रिया को आसान बना सकते हैं?
- Why do you think the process isn't like this now?
  आप ऐसा क्यों सोचते हैं कि पुराना वाला तरीका अब बदल गया है?

10. Closing
समाप्ति

Thank you for your time and for telling me so much about MCTS/RCH.
आपने मुझे अपना बहुमूल्य समय दिया और एमसीटीएस/आरसीएच के बारे में इतनी बातें बताई, इसके लिए आपके आभारी हैं.

- Would you like to add anything else about the accuracy and completeness of mobile phone registration in MCTS/RCH?
  एमसीटीएस / आरसीएच में मोबाइल फोन के पंजीकरण की सटीकता और पूर्णता के सन्दर्भ में कुछ और जोड़ना चाहेंगे/चाहेंगी?

For peer review only - http://bmjopen.bmj.com/site/about/guidelines.xhtml

FOCUS GROUP DISCUSSION (BENEFICIARIES) GUIDE

समूह केन्द्रित बातचीत - निर्देशिका

Participants:
भागीदार:

- Have a mobile phone number

जिनके पास मोबाइल फ़ोन नंबर हो

- Are pregnant or recently delivered

जो गर्भवती हों या जिन्हें अभी-अभी बच्चा हुआ है

- Recently interacted with an ANM, government nurse or doctor

जिन्होंने अभी-अभी एएनएम, सरकारी नर्स या डॉक्टर से संपर्क किया हो

Thank you for coming to this discussion. We're interested in understanding more about how pregnant women interact with government health care workers. This means mostly ANM and ASHA but also maybe the doctor, nurse and anganwadi worker. Please feel free to speak openly about how you feel
आप इस बातचीत के लिए आयीं, इसके लिए आपको बहुत-बहुत धन्यवाद. हम इस बात को अच्छे तरीके से समझना चाहते हैं कि गर्भवती महिलाएं सरकारी स्वास्थ्य-सेवा कर्मियों से किस तरह संपर्क करती हैं. स्वास्थ्य-सेवा कर्मियों से यहाँ मतलब, ज्यादातर, एएनएम और आशा कर्मियों से है, लेकिन शायद वे डॉक्टर, नर्स और आंगनवाड़ी कर्मी भी हो सकती हैं. आप अपनी बात खुल कर कहने के लिए आज़ाद हैं कि आपका अनुभव कैसा रहा.

11. Introduction

परिचय

क्या सभी लोग अपनी उम्र बता सकती हैं और यह भी कि आपको खाने में क्या पसंद है!

12. Late ANC presentation

लेट एएनसी प्रेजेंटेशन

- Around here, when do women generally first meet with ANM didi or ASHA didi about their pregnancy?

अपने गर्भवती होने के सिलसिले में यहाँ आस-पास की महिलाएं पहली बार साधारणतया एएनएम दीदी और आशा दीदी से कब मिलती हैं?

- How do they decide when to meet with ASHA didi? With ANM didi?

एएनएम दीदी और आशा दीदी से मिलना है, इस निश्चय का फैसला वे कैसे करती हैं?

Some women may come at the very beginning of their pregnancy, right when they think they are pregnant. Maybe you did this or someone you know.

For peer review only - http://bmjopen.bmj.com/site/about/guidelines.xhtml

कुछ औरतें शायद अपने गर्भधारण के शुरुआत में ही, जैसे ही वे सोचती हैं कि वे गर्भवती हैं, एएनएम दीदी और आशा दीदी से मिलने चली आती हैं. शायद आपने ऐसा किया है या किसी को जानती हैं, जिसने ऐसा किया है.

- What are some reasons that women might come early? Which women might meet ANM didi early?

वैसे कौन-से कारण हैं जिनके चलते महिलाएं जल्द ही एएनएम दीदी से मिलने आ सकती हैं? वे कौन-सी महिलाएं हैं जो एएनएम दीदी से जल्द ही मिल सकती हैं?

Some women may come later, after they have been pregnant for a while.
कुछ महिलाएं शायद बाद में एएनएम दीदी से मिलने आती हैं, जब गर्भधारण किये उन्हें कुछ समय बीत जाता है.

- What are some reasons that women might come late? Which women might meet ANM didi late?

वैसे कौन-से कारण हैं जिनके चलते महिलाएं बाद में एएनएम दीदी से मिलने आ सकती हैं? वे कौन-सी महिलाएं हैं जो एएनएम दीदी से बाद में मिल सकती हैं?

Some women might not meet ANM didi at all.
कुछ महिलाएं एएनएम दीदी से एकदम ही नहीं मिलती हैं.

- What are some reasons that women might not ever meet ANM did? Which women might not meet ANM didi at all?

वैसे कौन-से कारण हैं जिनके चलते महिलाएं एएनएम दीदी से मिलने एकदम ही नहीं आ सकती हैं? वे कौन-सी महिलाएं हैं जो एएनएम दीदी से मिलने एकदम ही नहीं आ सकती हैं?

In some places they say it's better not to tell people about a pregnancy, to avoid bad influences coming.
कुछ जगहों पर लोग कहते हैं कि गर्भवती होने की सूचना लोगों को नहीं देना अच्छा है, ऐसा करके बुरे प्रभावों से बचा जा सकता है.

- Can you tell me about what people say around here? How do people keep safe during pregnancy?

क्या आप मुझे बता सकती हैं कि यहाँ आस-पास के लोग क्या कहते हैं? लोग गर्भावस्था दौरान खुद को कैसे सुरक्षित रखती हैं?

13. Written records
लिखित दस्तावेज

When you visit with ASHA or ANM didi, or even when you visit with the nurse or doctor at the health centre, they ask some questions and write down some things. जब आप आशा दीदी या एएनएम दीदी से मिलने जाती हैं, यहाँ तक कि जब स्वास्थ्य-केंद्र के नर्स या डॉक्टर से मिलने जाती हैं, तब वे कुछ सवाल पूछते हैं और उत्तर सुनते हुए कुछ लिखते रहते हैं.

- Can you recall about what things they ask you?

For peer review only

क्या आप याद करके पता सकती हैं कि उन्होंने ने क्या-क्या पूछा?

If they can't recall, probe or jog memory: Sometimes women might be asked about their last menstrual period or their age. [Show the state's ANC registration card, e.g. the Rajasthan Mamta Card]
यदि वे याद नहीं कर सकती हैं, तब उनके दिमाग की थाह लेने या दिमाग पर जोर डालने की कोशिश करें: कभी-कभी महिलाओं से उनकी उम्र या अंतिम माहवारी कब आई जैसे सवाल पूछे जाते हैं. [राज्य का एएनसी पंजीकरण कार्ड दिखायें. जैसे राजस्थान ममता कार्ड]

- How do women feel when ANM didi/doctor sir asks questions?

एएनएम दीदी/डॉक्टर जब पूछते हैं, तब महिलाएं कैसा महसूस करती हैं?

Sometimes questions can be asked in a nice way and other times they are asked in a way that makes women feel bad, rushed, or unsure.
कभी-कभी सवाल पूछने का उनका तरिका बहुत अच्छा होता लेकिन कभी ऐसा भी होता है कि सवाल पूछने का उनका तरीका महिलाओं पसंद नहीं आता, या ऐसा लगता है कि सवाल पूछने में जल्दबाजी की जा रही, या सवाल स्पष्ट नहीं हो पाता है.

- Can you pretend to be a nurse, ANM or doctor and show me how they ask questions nicely?

क्या आप थोड़े समय के लिए एएनएम दीदी या डॉक्टर बनकर यह बता सकती हैं कि सवाल पूछने का अच्छा तरीका क्या होता है?

- Can you pretend to be a nurse, ANM or doctor and show me how they can ask questions in a bad or not nice way?

क्या आप थोड़े समय के लिए एएनएम दीदी या डॉक्टर बनकर यह बता सकती हैं कि सवाल पूछने का तरीका बुरा है या यह ढंग अच्छा नहीं है.

- Which questions are nice and easy to answer? What are some reasons for this?

वे कौन-से सवाल हैं जो अच्छे हैं और जिसका उत्तर देना आसान है? इसके कारण क्या हैं?

- Which questions might some women find difficult to answer or not nice?

वैसे कौन-से सवाल हैं जिसके उत्तर देने में कुछ महिलाओं को दिक्कत हो सकती है या उत्तर देना अच्छा नहीं है?

- What are some reasons for this?

इसके क्या कारण हैं?

- Which women might struggle a lot to answer questions?

For peer review only - http://bmjopen.bmj.com/site/about/guidelines.xhtml

वे कौन-सी महिलाएं जिन्हें सवालों के उत्तर देने में बहुत संघर्ष करना पड़ सकता है?

14. Mobile phone number

मोबाइल फ़ोन नंबर

One thing that women might be asked is for their mobile phone number.
एक बात यह भी है कि महिलाओं से उनके मोबाइल फ़ोन नंबर के बारे पूछा जा सकता है.

- How might women feel when they are asked by ANM didi for their mobile numbers?

एएनएम दीदी द्वारा जब उनके मोबाइल फ़ोन नंबर पूछे जाते हैं तब महिलायों को कैसा लगता है?

- What are some reasons why ANM didi might ask for a mobile number?

वे कौन-से कारण हैं, जिसके चलते एएनएम दीदी मोबाइल फ़ोन नंबर पूछ सकती हैं?

- If she did not have a mobile number, how might she feel? What might she do?

यदि उनके पास मोबाइल फ़ोन नंबर नहीं है तब उन्हें कैसा महसूस होता है? ऐसी स्थिति में वे क्या कर सकती हैं?

Some women might feel happy to give their mobile number, but others might not want to give it.
कुछ महिलाएं एएनएम दीदी को मोबाइल फ़ोन नंबर देने में ख़ुशी महसूस कर सकती हैं, लेकिन कुछ ऐसी भी हो सकती हैं जो मोबाइल फ़ोन नंबर देना नहीं चाहती हों.

- What might be some reasons why a woman would not want to give her mobile number?

वैसे कौन-से कारण हो सकते हैं कि एक महिला मोबाइल फ़ोन नंबर देना नहीं चाहती.

Sometimes a woman wants to give her mobile number but she does not recall what it is.
कभी-कभी कोई महिला अपना मोबाइल फ़ोन नंबर एएनएम दीदी को देना चाहती है लेकिन वह इस बारे में स्पष्ट नहीं होती है कि इससे क्या होगा.

- Could this happen? Tell me about how she would feel. Tell me about what might happen next.

क्या ऐसा हुआ है? यदि हाँ, तो वह यह जानकार कैसा महसूस करेगी, इसके बारे में बताएं. इसके बारे में भी बताएं कि इसके बाद क्या हो सकता है.

15. Corrections and updates to registered mobile numbers

पंजीकृत मोबाइल नंबरों को संशोधित कर उसे अपडेट करना/करवाना

Sometimes a person will get a new SIM so the mobile number will change. Sometimes they realize the mobile number recorded with the government is not correct. Either way, this means that the government has got the wrong number.

कभी-कभी कोई नया सिम लेगा, ऐसी स्थिति में मोबाइल नंबर चेंज हो जाएगा. कभी-कभी उन्हें लगता है कि सरकारी दस्तावेजों में दर्ज उनका मोबाइल नंबर सही नहीं है. अन्य तरीके से कहें तो इसका मतलब यह है कि सरकार को जो नंबर उपलब्ध है, वह गलत है.

- How might a woman feel if this happened? E.g. if she realized the government recorded a different number - an old number or a wrong number?

यदि ऐसा हुआ है, तब एक महिला के लिया यह अनुभव कैसा होता है? जैसे, यदि उसे इसका भान हो गया कि सरकार के यहाँ दर्ज उसका मोबाइल नंबर पुराना या गलत है?

- What could she do? Who might she speak to? What might happen if she tried to change it? Where would she go?

ऐसी स्थिति में वह क्या कर सकती है? वह कौन हो सकता है जिससे वह इसके सम्बन्ध में बात कर सकती है? क्या हो सकता है, यदि उसने मोबाइल नंबर चेंज करवाने की कोशिश की? वह कहाँ जायेगी?

- Some women might not try to change or add their number, if they realized it was wrong. Why might this be?

कुछ औरतें अपने मोबाइल नंबर को बदलवाने या जोड़वाने की कोशिश नहीं कर सकती हैं, बाद उन्हें पता लगा कि यह गलत था. ऐसा क्यों हुआ?

For peer review only

COREQ (COnsolidated criteria for REporting Qualitative research) Checklist

A checklist of items that should be included in reports of qualitative research. You must report the page number in your manuscript where you consider each of the items listed in this checklist. If you have not included this information, either revise your manuscript accordingly before submitting or note N/A.

Topic	Item No.	Guide Questions/Description	Reported on Page No.
**Domain 1: Research team and reflexivity**			
Personal characteristics			
Interviewer/facilitator	1	Which author/s conducted the interview or focus group?	
Credentials	2	What were the researcher's credentials? E.g. PhD, MD	
Occupation	3	What was their occupation at the time of the study?	
Gender	4	Was the researcher male or female?	
Experience and training	5	What experience or training did the researcher have?	
Relationship with participants			
Relationship established	6	Was a relationship established prior to study commencement?	
Participant knowledge of the interviewer	7	What did the participants know about the researcher? e.g. personal goals, reasons for doing the research	
Interviewer characteristics	8	What characteristics were reported about the inter viewer/facilitator? e.g. Bias, assumptions, reasons and interests in the research topic	
**Domain 2: Study design**			
Theoretical framework			
Methodological orientation and Theory	9	What methodological orientation was stated to underpin the study? e.g. grounded theory, discourse analysis, ethnography, phenomenology, content analysis	
Participant selection			
Sampling	10	How were participants selected? e.g. purposive, convenience, consecutive, snowball	
Method of approach	11	How were participants approached? e.g. face-to-face, telephone, mail, email	
Sample size	12	How many participants were in the study?	
Non-participation	13	How many people refused to participate or dropped out? Reasons?	
Setting			
Setting of data collection	14	Where was the data collected? e.g. home, clinic, workplace	
Presence of non-participants	15	Was anyone else present besides the participants and researchers?	
Description of sample	16	What are the important characteristics of the sample? e.g. demographic data, date	
Data collection			
Interview guide	17	Were questions, prompts, guides provided by the authors? Was it pilot tested?	
Repeat interviews	18	Were repeat inter views carried out? If yes, how many?	
Audio/visual recording	19	Did the research use audio or visual recording to collect the data?	
Field notes	20	Were field notes made during and/or after the inter view or focus group?	
Duration	21	What was the duration of the inter views or focus group?	
Data saturation	22	Was data saturation discussed?	
Transcripts returned	23	Were transcripts returned to participants for comment and/or	

For peer review only

Topic	Item No.	Guide Questions/Description	Reported on Page No.
		correction?	
**Domain 3: analysis and findings**			
Data analysis			
Number of data coders	24	How many data coders coded the data?	
Description of the coding tree	25	Did authors provide a description of the coding tree?	
Derivation of themes	26	Were themes identified in advance or derived from the data?	
Software	27	What software, if applicable, was used to manage the data?	
Participant checking	28	Did participants provide feedback on the findings?	
Reporting			
Quotations presented	29	Were participant quotations presented to illustrate the themes/findings? Was each quotation identified? e.g. participant number	
Data and findings consistent	30	Was there consistency between the data presented and the findings?	
Clarity of major themes	31	Were major themes clearly presented in the findings?	
Clarity of minor themes	32	Is there a description of diverse cases or discussion of minor themes?	

Developed from: Tong A, Sainsbury P, Craig J. Consolidated criteria for reporting qualitative research (COREQ): a 32-item checklist for interviews and focus groups. *International Journal for Quality in Health Care*. 2007. Volume 19, Number 6: pp. 349 – 357

Once you have completed this checklist, please save a copy and upload it as part of your submission. DO NOT include this checklist as part of the main manuscript document. It must be uploaded as a separate file.

For peer review only
